# Mental disorder, psychological problems and terrorist behaviour: A systematic review and meta‐analysis

**DOI:** 10.1002/cl2.1268

**Published:** 2022-08-10

**Authors:** Kiran M. Sarma, Sarah L. Carthy, Katie M. Cox

**Affiliations:** ^1^ School of Psychology National University of Ireland Galway (University of Galway) Galway Ireland; ^2^ Institute of Security and Global Affairs Leiden University Leiden The Netherlands

## Abstract

**Background:**

The link between mental health difficulties and terrorist behaviour has been the subject of debate for the last 50 years. Studies that report prevalence rates of mental health difficulties in terrorist samples or compare rates for those involved and not involved in terrorism, can inform this debate and the work of those responsible for countering violent extremism.

**Objectives:**

To synthesise the prevalence rates of mental health difficulties in terrorist samples (Objective 1—Prevalence) and prevalence of mental health disorders pre‐dating involvement in terrorism (Objective 2—Temporality). The review also synthesises the extent to which mental health difficulties are associated with terrorist involvement compared to non‐terrorist samples (Objective 3—Risk Factor).

**Search Methods:**

Searches were conducted between April and June 2022, capturing research until December 2021. We contacted expert networks, hand‐searched specialist journals, harvested records from published reviews, and examined references lists for included papers to identify additional studies.

**Selection Criteria:**

Studies needed to empirically examine mental health difficulties and terrorism. To be included under Objective 1 (Prevalence) and Objective 2 (Temporality), studies had to adopt cross‐sectional, cohort, or case‐control design and report prevalence rates of mental health difficulties in terrorist samples, with studies under Objective 2 also needing to report prevalence of difficulties before detection or involvement in terrorism. For Objective 3 (Risk Factor) studies where there was variability in terrorist behaviour (involved vs. not involved) were included.

**Data Collection and Analysis:**

Captured records were screened in *DisillterSR* by two authors. Risk of bias was assessed using *Joanna Briggs Institute* checklists, and random‐effects meta‐analysis conducted in Comprehensive Meta‐Analysis software.

**Results:**

Fifty‐six papers reporting on 73 different terrorist samples (i.e., studies) (*n* = 13,648) were identified. All were eligible for Objective 1. Of the 73 studies, 10 were eligible for Objective 2 (Temporality) and nine were eligible for Objective 3 (Risk Factor). For Objective 1, the life‐time prevalence rate of diagnosed mental disorder in terrorist samples (*k* = 18) was 17.4% [95% confidence interval (CI) = 11.1%–26.3%]. When collapsing all studies reporting psychological problems, disorder, and suspected disorder into one meta‐analyses (*k* = 37), the pooled prevalence rate was 25.5% (95% CI = 20.2%–31.6%). When isolating studies reporting data for any mental health difficulty that emerged before either engagement in terrorism or detection for terrorist offences (Objective 2: Temporality), the life‐time prevalence rate was 27.8% (95% CI = 20.9%–35.9%). For Objective 3 (Risk Factor), it was not appropriate to calculate a pooled effect size due the differences in comparison samples. Odds ratios for these studies ranged from 0.68 (95% CI = 0.38–1.22) to 3.13 (95% CI = 1.87–5.23). All studies were assessed as having high‐risk of bias which, in part, reflects challenges conducting terrorism research.

**Author's Conclusions:**

This review does not support the assertion that terrorist samples are characterised by higher rates of mental health difficulties than would be expected in the general population. Findings have implications for future research in terms of design and reporting. There are also implications for practice with regards the inclusion of mental health difficulties as indicators of risk.

## 1 PLAIN LANGUAGE SUMMARY

### 1.1 Inconsistent findings on mental health difficulties and risk of involvement in terrorism

There has been an increasing focus on the potential role of mental health difficulties in the process of violent radicalisation into terrorism. In part, this has been fuelled by studies appearing to show high prevalence rates in some samples of terrorists. However, findings are inconsistent, with some studies reporting higher rates than those observed in the general population, some lower, and others that are comparable to those observed in the general population.

### 1.2 What is this review about?

This review synthesises the prevalence rates of mental health difficulties in terrorist samples (Objective 1—Prevalence) and prevalence of mental health disorders pre‐dating involvement in terrorism (Objective 2—Temporality). The review also synthesises the extent to which mental health difficulties are associated with terrorist involvement compared to non‐terrorist samples (Objective 3—Risk Factor).

In addressing these objectives, the review offers an initial assessment of what we refer to as the mental health‐terrorism hypothesis (that mental health difficulties are a risk factor for terrorist involvement). Tentative support for the hypothesis would be provided where studies, when collated, suggest higher rates of difficulties in terrorist samples than those expected in the community.

The review distinguishes between mental disorders, suspected mental disorders and psychological problems. These are collectively termed mental health difficulties.

**What is the aim of this review?**

This Campbell Collaboration systematic review focuses on mental health and its association with terrorist involvement. The review examines evidence based on 56 papers reporting on 73 terrorist samples.

### 1.3 What studies are included?

For Objective 1, studies that report rates of mental health difficulties in terrorist samples are included. Studies were eligible even if the period after the terrorists became involved in terrorism is included. We identified 56 papers reporting on 73 terrorist samples that met this criterion.

For Objective 2, studies from Objective 1 were included where they specifically reported rates of difficulties and where those difficulties emerged before the terrorist became involved in terrorism (or was first detected as being involved). Ten studies were included in this component of the review.

Finally, for Objective 3, we included studies that compared rates of mental health difficulties in terrorist samples with non‐terrorist samples. Nine eligible studies were included here.

### 1.4 What are the findings of the review?

Our findings do not support the assertion that there are remarkably high rates of mental health difficulties in the terrorist population. As a benchmark, we estimate that the lifetime prevalence rate of diagnosed mental disorder in the general population is 29%. For Objective 1 (Prevalence) we report that the lifetime prevalence rate of diagnosed mental disorder in terrorist samples was 17.4%. This increased to 23.2% for the studies reporting lifetime prevalence rates of suspected disorder, and 28.5% for studies reporting any psychological problems.

At any one time, 14.4% of those involved in terrorism may have a disorder or suspected disorder (as opposed to a psychological problem). When we isolated studies that reported rates prior to either engagement in terrorism or detection for terrorist offences (Objective 2, Temporality), the lifetime prevalence rate for any psychological problem was 27.8%.

Finally, based on single study estimates, the odds of having a lifetime history of mental health difficulties between those involved in terrorist behaviour and non‐terrorist offending varied depending on the comparison group (Objective 3, Risk Factor).

### 1.5 What do the findings of the review mean?

The findings do not offer support for the mental health‐terrorism hypothesis. Trends in the data, however, may point to higher rates among some terrorist samples than others, and in particular among lone‐actor terrorists.

The synthesis could reflect methodological limitations in the studies included. Many studies rely on the media and court reporting, with researchers wholly reliant on these sources to determine whether or not mental health difficulties are present. This could lead to under‐reporting (where such difficulties are not viewed as relevant to a criminal case for example) and thus deflate estimates reported in papers.

That said, even among those papers that have not relied on open‐source information, the evidence does not conclusively demonstrate that terrorist samples are characterised by higher rates of mental disorders or psychological problems than those expected in the community.

### 1.6 How up‐to‐date is this review?

The review authors searched for sources in April and May 2022, covering research up to December 2021.

## 2 BACKGROUND

### 2.1 The problem, condition or issue

In the period 1970–1990 there was extensive debate on the potential link between mental disorder and terrorism (Cooper, 1978; Ferracuti & Bruno, 1981; McCauley & Segal, 1987; Rasch, 1979; Shaw, 1986; Silke, 1998; Smith & Morgan, 1994; Wardlaw, 1982). Commentators proposed that some of those who became involved in terrorism had an underlying mental disorder that was causally linked to their violence propensity, including, for example, a range of personality disorders (Cooper, 1978; Lasch, 1979; Pearce, 1977). However, successive studies failed to support this link (e.g., Elliot & Lockhart, 1980; Ferracuti & Bruno, 1981; Lyons and Harbinson, 1986) leaving many convinced that those involved in groups like the Provisional IRA and Euskadi Ta Askatasuna (ETA) were no different to the general population in terms of mental health difficulties and were, instead, attracted to terrorism by virtue of a multitude of interacting psychosocial processes (e.g., Crenshaw, 1981; Heskin, 1984; Taylor & Horgan, 2006). The assertion that terrorism was the product of abnormality was ultimately deemed ‘unfair’ to the terrorist and abandoned by researchers and policy makers (e.g., Silke, 1998).

In recent times the debate has re‐emerged, fuelled in part by a more nuanced approach where the focus has been on specific forms of terrorism (e.g., lone actor terrorist or right‐wing terrorism) rather than terrorism in general. For example, those interested in so‐called lone actor violence raised concerns about ‘fundamental errors’ in past research (Corner et al., 2016, p. 561) and presented plausible case formulations linking experiential stressors such as social isolation with mental disorder and violence (e.g., Corner & Gill, 2015). Cohort studies of lone actors emerged that appeared to show high rates of mental disorder, with 37% of Liem and colleagues' sample of European lone actor terrorists having ‘some indication of mental illness’, and 25% being clinically diagnosed with ‘a particular mental disorder’ (Liem et al., 2018, p. 60). Such findings were not limited to lone‐actor terrorism (e.g., Weenink, 2015).

However, those who have looked more closely at this evidence base have acknowledged that the picture emerging is far from clear, with the lack of clarity attributable, in part at least, to methodological limitations in that literature (Gill et al., 2021; Jensen et al., 2020). To some extent these limitations centre on one core problem–the difficulty in determining to what extent, if any, the presence of a mental disorder *confers* risk of terrorist involvement (as opposed to being associated with increased risk). To credibly conclude that disorder (or, more broadly, psychological problems) can increase the risk of becoming involved in terrorism, we suggest that the existing literature needs to demonstrate that certain criteria are met.

The first criterion relates to the *prevalence* of disorder among terrorist samples, and is termed here ‘the prevalence problem’. Assessing prevalence of mental disorder among terrorist samples using appropriate comparators sheds light on the magnitude of the relationship (if any) between the risk and outcome. It is an important criterion for determining causality and one of several Bradford‐Hill criteria for causation (Hill, 1965). Reporting and interpretating prevalence rates requires careful consideration of the distinction between point and period prevalence, and use of appropriate comparison groups. If mental disorder confers risk of terrorism, then the prevalence rate amongst terrorist samples should be higher than that expected in the general population (matched on key demographics such as age, gender and geographic location). A prerequisite for making such comparisons is synthesising the prevalence rates of mental disorders, and more broadly psychological problems, in terrorist samples.

The second criterion is that of *temporality* (see Lucas & McMichael, 2005), another Bradford‐Hill criterion (and termed here ‘the temporality problem’). To demonstrate that mental disorder confers risk of involvement in terrorism, then studies must demonstrate that the disorder emerges before involvement in terrorism. If we cannot demonstrate temporality, then differentiating cause and effect is problematic—disorder could be the consequence of terrorist involvement rather than a cause of involvement. The question that must be asked of the literature, then, is: To what extent do findings in the literature support the assertion that earlier mental disorder confers risk of terrorist involvement later in life?

The third criterion that must be met to infer a causal link exists between mental disorder and terrorism is that of *plausibility*; offering plausible explanations for just how the disorder (or disorders) confers risk of terrorism involvement (the Bradford‐Hill ‘plausibility problem’). Addressing the plausibility problem is hampered by the heterogenous nature of terrorism (the outcome of interest), controversy around the concept of ‘mental disorder’ (the potential risk factor of interest) and, finally, the complexity of the relationship that may exist between terrorism and mental health difficulties.

A final consideration for those conducting research on terrorism and mental disorder relates to the concept of ‘mental disorder’ itself. To illustrate the difficulty with the concept, it is worth referring to the recent contribution of Bakker (2019). Bakker's thesis, like many other clinical psychologists, is that the concept of ‘mental disorder’ is fundamentally flawed, a ‘medical nosology of diseases’ that does little to aid our understanding of clients or how best to intervene to alleviate their distress (p. 1). What is required, they argue, is a paradigm shift towards a focus on ‘psychological problems’ which Bakker defines as ‘a negative psychological‐level state of affairs’ (p. 10). These psychological problems are transdiagnostic (appear across diagnoses and capture the common difficulties reported by patients) and may or may not require intervention. Bakker's thesis draws attention to two types of mental health difficulties—mental disorders and psychological problems.

The difficulty for the terrorism literature is that it is not always clear what the outcome of interest is, particularly when terms like disorder, mental health difficulties, psychological distress, etc. are used interchangeably. Yet, where a formal diagnosis is not made by a mental health professional, then the presence of a mental disorder cannot be inferred. At best, we can conclude that the individual has or had what Bakker refers to as a psychological problem.

This review seeks to contribute to our understanding of the potential link between terrorism and mental health difficulties by focusing on the problems of prevalence, temporality and plausibility, while being sensitive to the distinction between mental disorder and psychological problems. Specifically, the review will present a synthesis of the evidence on prevalence, temporality and plausibility drawing on the best evidence available. In reviewing prevalence, temporality and plausibility, we are presenting an initial test of what we refer to as the mental health—terrorism hypothesis: That mental health difficulties confer risk of involvement in terrorist behaviour.

### 2.2 Outcome—Terrorist behaviour

The outcome of interest in this systematic review is terrorist behaviour. While there is no universally accepted definition of terrorist behaviour (Ganor, 2002; Saul, 2019; Silke, 1996), there is at least some consensus that it refers to: *(a)* an act or campaign of actual or threatened violence that seeks to elicit the terror emotion in a target audience and; *(b)* with the intention of bringing about change in line with the world‐view of the terrorist (e.g., Kruglanski & Fishman, 2006; Moghaddam, 2007). Terrorist behaviour intends to cause harm, physical or otherwise (Van Der Does et al., 2021).

One complexity encountered by those conducting research in the area of terrorism is the heterogeneity of the phenomenon itself (Herrington & Roberts, 2012; Monahan, 2012; Roberts & Horgan, 2008). For example, one study of 176 terrorist organisations identified 33 different ideologies as well as diversity in terms of size, organisational structure, geographic location, and lethality (Cook & Lounsbery, 2011). It cannot be assumed that individuals who become involved in different forms of terrorism do so through the same processes (Change Institute, 2008). The implications of this heterogeneity, for the proposed review, are that, when we refer to terrorism, we specify this behaviour in terms of the ‘type’ of terrorism waged.

The review excludes violent radicalisation as an outcome, understood within this study to refer to a process of growing acceptance of the legitimacy of violence to bring about societal change (McCormick, 2003). We justify this exclusion on the following basis:

1. Violent radicalisation and terrorism are conceptually non‐synonymous. Terrorist behaviour is *action* orientated whilst violent radicalisation is a process whereby individuals become increasingly committed to the use of violence, yet may not necessarily perpetrate violence (i.e., it can be cognitively orientated) (Sarma, 2017).
2. Of the significant minority of those who accept the legitimacy of terrorist violence (e.g., PEW, 2013), a small proportion transition into terrorism (Christmann, 2012). Those who transition into terrorism may differ from those who do not on both dispositional (e.g., morality, aptitude, motivation, etc.) or situational (e.g., opportunity) levels. If so, the findings from the literature on violent radicalisation will lack external validity when applied to terrorist behaviour.

### 2.3 Potential risk factor—Mental health difficulties

The review informs our understanding of the potential link between mental disorder and terrorist involvement. However, we are acutely aware of the limitations of the largely disease‐orientated focus of ‘mental disorders’, and for the reasons set out below extend our focus to include a more transdiagnostic lens by considering, broadly, psychological problems.

The American Psychological Association defines mental disorder as any condition ‘characterized by cognitive and emotional disturbances, abnormal behaviours, impaired functioning, or any combination of these’ (American Psychological Association, 2020). Diagnostic manuals, such as iterations of the Diagnostic and Statistical Manual of Mental Disorders (DSM; American Psychiatric Association, 2013) and International Classification of Diseases (ICD; World Health Organization WHO, 2019), present diagnostic criteria for a range of mental disorders, and diagnoses can be made based on the combination of symptoms and their severity.

While diagnostic systems convey a sense that mental disorders are discrete psychological experiences, Allsopp and colleagues note that diagnostic systems are characterised by*: (a)* varying diagnostic rules across presentations; *(b)* overlap in symptoms across diagnoses and; *(c)* a tendency for diagnostic labels to mask the root causes of distress and problematic behaviours (Allsopp et al., 2019). They also argue that diagnoses can distract from the real‐world work of reaching an in‐depth understanding of the individual (see also Galatzer‐Levy & Bryant, 2013; Olbert et al., 2014).

The use of diagnostic systems in terrorism research is particularly problematic. Mental disorders can only be said to be present where they have been diagnosed by an appropriately trained mental health professional. The presence of a disorder, as characterised in DSM or ICD, cannot be reliably inferred from reports of symptoms present in open‐source data alone (e.g., press coverage of trials of suspected terrorist offenders). It requires careful assessment by a professional, often in collaboration with the individual being assessed, of the presence or absence of various criteria. Where the results of such assessment are made available for the purposes of research, then we can have at least some confidence in the diagnostic process.

However, the data on terrorist offenders does not always provide access to data gathered through structured clinical assessment by a suitably trained professional. Some of the most widely cited studies in the area, for example, are based on open‐source information derived from the media coverage, etc., of terrorists, and where inferences are made as to the presence/absence of disorder based on difficulties experienced by the terrorist and publicly reported (e.g., Gill et al., 2014). Yet it is not always clear if these difficulties are any different to those experienced in the normal course of one's life.

Bakker (2019) has discussed this in the context of differentiating between ‘psychological problems’ that ‘do not self‐perpetuate… and tend to ease without interventive therapy’ (p.11) and ‘clinical psychological problems’. The former, Bakker argues, are normal adaptive processes that might include an experience of depression after a bereavement, but which follows the normal course of recovery. The latter, however, might take the form of avoidance, be pervasive and enduring, impair one's quality of life, and require intervention to resolve.

In the review we attend to the literature on mental disorder and psychological problems. While our initial intention was to differentiate between psychological problems that are ‘clinical’ (required intervention) and those that were not clearly clinical (no evidence of a requirement of intervention), no papers that met our eligibility criteria made such a distinction. Synthesising the literature on ‘mental disorder’ provides coverage of the diagnostic literature. Attending to the broader literature on psychological problems captures a wider body of literature and means that our review is not constrained by the limitations of the psychiatric model (see ‘*Inclusion Criteria*’ for additional detail).

### 2.4 How mental disorder and psychological problems may be linked to terrorist behaviour

The link between mental disorder and forensic risk has been the subject of research for decades. Findings are unclear and inconsistent, with some studies appearing to link disorder to violence risk, while others have reported no link (e.g., Augestad Knudsen, 2020; Bhui & Jones, 2017). There appears to be three core complexities in this area that are directly relevant to an assessment of the link between terrorism and disorder.

The first is to do with the prevalence problem, as discussed earlier in this protocol, and which requires an assessment of the rates of mental disorders, and more broadly psychological problems, in terrorist samples. The second is *temporality*, initially discussed by Bradford Hill in their consideration of association and causality (i.e., one of the Bradford Hill criteria for causal inferences; see Lucas & McMichael, 2005). To argue that mental disorder confers risk of violence, research must demonstrate that the onset of psychological problems (Factor A) pre‐dates involvement in violence, or at the very least detection for terrorist offences (Factor B). If temporality cannot be established, there is a risk of misinterpreting correlation (where Factor A is associated with Factor B) as causation (Factor A *causes* Factor B).

For example, high rates of mental disorder are often observed among incarcerated violent offenders (O'Sullivan et al., 2018). A typical study examining the link between mental disorder and violent offending in this population will involve those incarcerated completing a battery of measures that assess psychological wellbeing and severity of offence (‘index offence’). Where a relationship emerges, the temptation is to conclude that higher levels of psychological distress confer risk of serious offending (Factor A causes Factor B). The problem here, however, is that the research design deployed cannot test causal relationships and is limited to measuring associations. Because we do not have longitudinal data showing levels of psychological symptoms before involvement in violence, we cannot exclude the possibility that the distress arose due to the index offence, or during incarceration for that offence. In such a case, incarceration/offending (Factor B) could cause distress (Factor A). This resonates with the conceptual difficulties surrounding the suggestion that pre‐existing Post‐traumatic Stress Disorder (PTSD) increases risk of terrorist involvement among so‐called ‘foreign fighters’ and where PTSD is assessed after they have returned from conflict (e.g., Al‐Attar, 2019).

Even where temporality is determined, we also need to consider the ‘Third Variable Problem’. Here an apparent causal relationship between disorder and violence may be explained by a lurking third variable (Variable X) that influences both factors and causes them to co‐vary. This might arise, for example, where experiences of discrimination and isolation drive both mental disorder and violent radicalisation. Here the apparent relationship between mental disorder and violent radicalisation may be wholly attributable to situational stressors and intervening to manage disorder may not reduce risk.

In reality, there is unlikely to be a large body of scientifically robust longitudinal evidence that addresses both temporality and the ‘Third Variable Problem’. This is particularly the case in the area of terrorism studies where the problem of concern is so difficult to expose to academic enquiry due to its low base rate in the population (Sarma, 2017). In such a situation, another of Hill's criteria becomes important—that of *plausibility*. That is, in assessing the relationship between mental disorder and violence risk, we must be able to present a plausible theoretical argument as to just how disorder confers risk. In clinical forensic practice, plausibility is addressed through careful assessment of forensic risk and the presenting of a theoretical argument (or formulation) that explains how the presence of risk factors may confer risk (Davies et al., 2013).

One way of presenting a formulation of risk is through the ‘4Ps’ Framework. The 4Ps Framework places a risk factor in a temporal space or chronology and proposes the nature of the relationship between the factor, other factors, and the outcome of interest. In doing so it differentiates between predisposing, precipitating, protective and perpetuating factors. It is widely used in both clinical and forensic psychology case formulation (e.g., Macneil et al., 2012).

The 4Ps framework can encourage a more nuanced consideration of the link(s) between terrorism and mental disorder. A predisposing risk factor is one that places the individual at risk of becoming involved in terrorism later in life. In the broader literature on clinical and clinical forensic psychology mental disorder is viewed as primarily a non‐causative background predisposition for becoming involved in violent behaviour and which is part of a complex set of interacting risk factors that together lead to a scaffolding of risk (e.g., Van Dorn et al., 2012). For example, Markowitz (2011) adopts a Social Disorganisation Theory approach to formulating risk in proposing that people with long‐term mental disorders are more likely to reside in disadvantaged communities characterised by socially disorganised neighbourhoods with a lack of health care, limited job opportunities, racial diversity and fragmented families. Crime flourishes in such criminogenic contexts, they argue, because there is a culture of acceptance of violent crime and poor social control over offending. This resonates with the terrorism literature, with some arguing that vulnerability to violent radicalisation is due, in part, to radicalising settings where some sections of the community endorse beliefs that justify terrorism (e.g., Schils & Pauwels, 2016).

A precipitating risk factor is one that apparently triggers a ‘crisis’. In the mental health literature, extreme situational stressors such as relationship break‐down, bereavement or other acute trauma can result in a cascade of events leading to the negative outcome (e.g., Barber et al., 2014). For mental disorder to precipitate involvement in terrorism, research would need to demonstrate that involvement in terrorism was immediately preceded by the onset of an episode of psychological distress that causally led to involvement in violence. In the broader clinical forensic literature, this has typically been associated with the presence of ‘positive’ psychotic symptoms, such as irrational (delusional) beliefs about others who subsequently become the target of violent intent (Markowitz, 2011). Of course, it could be reasonably argued that in some cases where terrorist behaviour is precipitated by a mental disorder, and derives from disorder (e.g., persecutory beliefs), that the issue of concern is clinical forensic risk rather than terrorism.

Some forms of mental disorder may actually preclude an individual from becoming involved in terrorism (i.e., it is a ‘protective factor’). For example, there is evidence that organisations like the Provisional IRA sought to recruit the most psychologically robust individuals into their ranks as a way of reducing the potential for members to be compromised and turn informer, or to provide information while being questioned by the police and security services (Sarma, 2005). Here mental disorder actually protects against involvement.

Finally, perpetuating factors, in the context of terrorism, serve to maintain the problematic behaviour, and thus hamper the ability of the individual to disengage from terrorism. In their review of push and pull factors that influence the ability of extremists to disengage from terrorism, Jensen and colleagues (2020) noted that increased social mobility, onset of new intimate romantic relationships, children, and access to rehabilitation services can support disengagement. Conversely, mental health difficulties can impede the ability of individuals to develop relationships, access services, and be more socially mobile, hampering disengagement. They conclude that ‘[e]specially when co‐occurring with substance abuse, mental illness can act as a strong barrier to disengagement, especially if it counteracts the feelings of disillusionment that otherwise may prompt one's exit’ (Jensen et al., 2020, p. 8).

### 2.5 Why it is important to do the review

As noted earlier, findings in the literature examining the link between terrorism and mental disorder are inconsistent (Ho et al., 2019). Where such inconsistencies are a feature of an evidence base, the cherry‐picking of results to suit a specific position can impede a nuanced understanding. Systematic reviews provide a synthesis of the available literature in one accessible paper and in doing so reduce bias (White & Waddington, 2012).

Gill and colleagues (2021) provide a valuable review of the literature exploring the link between mental disorder and terrorism. They note the heterogeneity in prevalence rates in the literature and provide some plausible explanations for this heterogeneity. Our review, however, we will attend in particular to point and period prevalence rates and include studies published since 2020. We will also present comprehensive data syntheses for both mental disorder and psychological problems and where appropriate present sub‐group analyses. Moreover, we will critically evaluate the appropriateness of the comparator populations being used as benchmarks for prevalence. This can help us more carefully consider the causal link, if any, between disorder/difficulties and terrorist behaviour.

We also considered Misiak et al. (2019) systematic review of the link between mental health and radicalisation and mass violence. While the review also presents a valuable contribution to the literature, it focuses primarily on the risk of radicalisation, with nine of the 12 studies included in their review using community samples and self‐reported radical beliefs (i.e., not terrorist samples). As noted above, we cannot assume that the literature on violent radical beliefs is valid for our understanding of terrorist behaviour.

Findings from the current systematic review will support a more informed debate on the link between mental health difficulties and terrorist behaviour. For each paper included in the review, we isolate and specify the prevalence being reported (e.g., present at time of assessment (point); childhood etc.). We will synthesise studies that are sensitive to temporal sequencing (temporality), where difficulties are onset before involvement in terrorism, and studies that examine the extent to which difficulties are associated with involvement (i.e., compare rates across two groups).

Apart from supporting debate in the area, the findings will be of value to a range of professionals who are responsible for risk assessment, risk mitigation and psychological intervention. In relation to risk assessment, for example, two popular risk assessment tools, the VERA 2R (Pressman et al., 2016) and ERG 22+ (Lloyd & Dean, 2015), both contain items relating to psychopathology despite concerns that (a) the evidence supporting their inclusion is contested and (b) there is a need to disaggregate disorders into various forms or problem clusters to determine which, if any, may be linked to risk of terrorist behaviour (see Herzog‐Evans, 2018). In supporting a more nuanced understanding of the link between disorder, psychological problems and terrorist behaviour our review will help guide the use of such tools and in doing so support decision making around psychological support, appropriate detention settings, and release from detention.

In relation to psychological intervention, there are multi‐disciplinary teams working in most countries tasked with supporting individuals who may at risk of transitioning into terrorism to redirect their lives towards non‐violence. In many cases these teams include health workers who are sensitive to psychological problems that are believed to exacerbate risk of becoming involved in terrorism—teams comprised of professionals who would benefit from a systematic review of the relevant literature. The work of such teams has been reviewed and discussed elsewhere (see Sarma, 2018, 2019a, 2019b).

## 3 OBJECTIVES

The first objective of the review (Objective 1—Prevalence) is to present a synthesis of the reported prevalence rates of mental health difficulties in terrorist samples. Where sufficient data is available, we also aimed to be sensitive to the heterogeneity of the terrorism phenomenon by exploring the rates of mental health difficulties for different forms of terrorism and for different terrorist roles (e.g., bombing, logistics, finance, etc.). The second objective (Objective 2—Temporality) is to synthesise the relevant literature where mental health difficulties pre‐date involvement in terrorism, again focusing on prevalence rates. Finally, the third objective (Objective 3—Mental health as a risk factor) examines the extent to which the presence of mental health difficulties confers risk of terrorist involvement by comparing those involved, and not involved, in terrorism.

## 4 METHODS

### 4.1 Criteria for considering studies for this review

#### 4.1.1 Types of studies

##### Objective 1: Prevalence

For our synthesis of prevalence rates of disorder and psychological problems the following types of studies were eligible:

1. Cross‐sectional studies reporting the prevalence of mental health difficulties as they exist in the population of interest (i.e., terrorist samples) at a particular time.
2. Cohort studies, both prospective and retrospective. In the prospective cohort study design, a cohort of individuals is identified and followed‐up over time to determine who did and did not become involved in terrorism. Within this analysis, the presence of mental health difficulties in the cohort will have been recorded at the initial screen. In a retrospective cohort study, the past incidence of disorder or problems in a group of individuals who became involved in terrorism will have been evaluated post‐hoc.
3. Case‐control studies in which individuals from the population of interest (i.e., those who engaged in terrorist behaviour) are compared to a group who have not perpetrated the behaviour (i.e., ‘controls’) and then concurrently (at time of study) or retrospectively assessed for mental health difficulties. The groups will have been compared with respect to the prevalence of mental health difficulties. For these studies, we intended to extract data from the terrorist subgroup to estimate prevalence.

To appropriately assess whether the prevalence of mental disorder and/or psychological problems are higher or lower in terrorist samples, we compared the prevalence rates of mental disorders and psychological problems to rates for the general population reported in national/global mental health estimates. Our approach for this is detailed in the ‘Data Synthesis’ section.

##### Objective 2: Temporality

If a study included under Objective 1 also presents data where inferences can be drawn as to the temporal onset of difficulties relative to involvement in terrorism, and where difficulties pre‐dates involvement, it is eligible for synthesis under Objective 2 (e.g., Bakker, 2006). We include here studies that report rates of mental health difficulties before being arrested for a detected terrorist offence. As mental health difficulties may have emerged in the period between first involvement in terrorism (often unknown) and the first time of arrest, we cannot always definitively establish that mental health difficulties precede terrorist behaviour in these studies. However, they provide preliminary indicators of temporality in the absence of more rigorous risk/predictive studies.

##### Objective 3: Risk factor

Objective 3 involved synthesising studies where there is variability in terrorist behaviour (i.e., some individuals engaged in terrorist behaviour and other individuals did not) and variability in mental health issues. Including case‐control designs where comparison groups are carefully matched to terrorist samples enables us to assess the extent to which mental health difficulties are a risk factor for terrorist involvement. To be eligible studies had to adopt prospective or retrospective cohort study designs or case‐control/cross‐sectional designs. Where inferences can be drawn about the temporal onset of the disorder relative to involvement in terrorism (i.e., if the problem or diagnosed disorder pre‐dates involvement in terrorism), this will augment our understanding of temporality. Where such inferences cannot be drawn, then studies will still inform our understanding of relative risk.

For all objectives, eligible studies had to provide details of the approach to data collection and the sampling strategy. Papers that reported such detail, and aligned with our other inclusion and exclusion criteria, were included. This included those published in journal articles, book chapters, books, conference presentations, conference publications, and unpublished reports.

We excluded qualitative papers from the synthesis unless they reported quantitative data on prevalence or relative risk. However, such studies were retained in a separate folder in the bibliographic database and used to aid our interpretation of the findings from the review. Similarly, we excluded studies using a case study design (e.g., Faccini & Allely, 2017), but draw on this literature for context.

We also excluded discussion papers, theoretical contributions, newspaper articles, blogs and any paper that did not detail a sampling strategy, approach to data collection, or empirical findings. Finally, literature reviews and systematic reviews were excluded from the synthesis but retained for the purpose of reverse searching for relevant publications (i.e., to harvest potentially relevant papers).

#### 4.1.2 Types of participants

For all three objectives, we included studies that contained at least some participants who are, or have been, involved in terrorist behaviour. As widely acknowledged in the literature, there have been different conceptualisations of terrorism and terrorist behaviour across studies and this has been identified as one of the primary impediments to primary research, synthesis and generalisability (e.g., McCann, 2020; Perliger et al., 2016). As a synthesis of the primary literature, the proposed review cannot overcome this limitation. However, it is critically important that the synthesis is sensitive to it. To that end, the review adopted the following approach:

1. We considered the process of being involved in terrorism as commencing when the individual *acts* to become involved. For example, an individual who attempts to travel abroad to become involved in terrorism (e.g., by booking flights), but is prevented from doing so by the authorities, meets this conceptualisation of terrorist behaviour (e.g., Weenink, 2015). However, someone who expresses an intention to travel abroad but has not taken to steps to travel, has not acted and thus is not conceptualised here as being involved in terrorist behaviour.
2. Participants in the studies included met at least one of the following criteria: (a) been convicted of a terrorist offence; (b) died in the perpetration or attempted perpetration of an attack; (c) been identified by the authorities as having been involved in terrorist behaviour or attempting to become involved (e.g., attempting to travel to join a terrorist organisation); (d) self‐report as being members/former members of a terrorist movement. We acknowledge that there are problems with these parameters, including that what constitutes a terrorist offence can vary from one jurisdiction to another (and even within jurisdictions; Schmid, 2004). We also acknowledge that terrorist behaviour is diverse, and may include the perpetration of violence, but also many other actions in support of terrorism (Altier et al., 2013). These may include the design and dissemination of propaganda, financing terrorism, recruitment, logistics and training.
3. Definitions of terrorism and terrorist behaviour were extracted from eligible papers, as were the forms of terrorism being studied and roles of participants, and we sought to be sensitive to this complexity in our aggregation (or disaggregation) and synthesis of the literature.
4. Participants could be of any age, gender or ethnicity.

#### 4.1.3 Types of outcome measures

For all three objectives the ‘measurement’ of terrorist involvement could include studies where participants are identified through:

1. Self‐report—Participants report that they are or were involved in terrorism.
2. Official sources—For example, law enforcement or security services identify participants who are involved (e.g., Weenink, 2019).
3. Arrest/prosecution/conviction—Participants are known to have been arrested, prosecuted or convicted of terrorist offences, potentially giving researchers an opportunity to conduct research during incarceration (e.g., Dhumad et al., 2020) or through a retrospective review of their lives (e.g., through open‐source research; e.g., Liem et al., 2018).

#### 4.1.4 Predictive/risk factor: Mental disorders and psychological problems

For all three objectives, the predictive/risk factor of interest needed to be mental disorder or psychological problems, collectively referred to here as ‘mental health difficulties’. Mental disorders are typically diagnosed by mental health professionals, such as psychiatrists and psychologists, following careful clinical assessment (e.g., structured clinical interviews) and formulation. It may also be diagnosed, for research purposes at least, through psychological testing, with either formal diagnostic tests (e.g., Millon Clinical Multiaxial Inventory III) or screening tools with established clinical cut‐offs. In our review, our search terms relating to mental disorder were based on the core categories of disorder listed in the DSM (version III (1980)–V (2013) and ICD (v. 10 & 11., World Health Organization WHO, 1992, 2019), as informed by an earlier review (Hossain et al., 2020), and listed in Table 1.

**Table 1:** Terms sensitive to disorders listed in DSM and ICD

Disorders		
ADHD	Depression	Obsessive
Alcohol	Depressive	Post‐traumatic Stress
Anorexia	Dissociative	PTSD
Antisocial	Drug	Schizophrenia
Anxiety	Dysphoria	Schizotypal
ASD	Eating	Panic
Attachment	Intellectual	Personality
Attention	Insomnia	Phobia
Autism	Learning	Psychotic
Bipolar	Motor	Trauma
Cognitive	Neurocognitive	Schizoaffective
Communication	Neurodevelopmental	Sleep
Compulsive	Oppositional	Somatic
Conduct	OCD	Stress
		Substance

There is no established taxonomy of psychological problems available. Bakker (2019) makes some suggestions as to the structure, and indicative content, of a taxonomy but does not present one. An alternative taxonomy, Hopwood et al. (2020) Hierarchical Taxonomy of Psychopathology, was considered but that taxonomy does not list symptoms or symptom components, leaving this to clinicians to identify based on a person‐centred assessment of their clients. While our review did not benefit from a transdiagnostic taxonomy, we are confident that the search terms used to identify mental disorders contained the core transdiagnostic features of psychological problems, thus allowing us to capture both disorder and problems in the same search syntax (e.g., ‘anxiety’ captured both anxiety disorder and anxiety as a transdiagnostic symptom). For additional cover, however, we included the following terms, and which were based on our review of some of the transdiagnostic features of mental health difficulties present in public health guidance (e.g., Health Direct Australia; 2021):

- Worried/Afraid
- Unhappy/Sad
- Emotional
- Quiet/Withdrawn
- Guilty/Worthless
- Suicide
- Suicidal Behaviour
- Self‐harm
- Mood
- Affective
- Addiction

Given that the presence of disorder or problems may be assessed at different phases of terrorist involvement (e.g., before involvement, during involvement or after exiting), we grouped data as follows:

1. Where the presence‐absence of disorder or problems was assessed while the individual is involved in terrorism, or incarcerated for terrorism offences, these studies were grouped together and referred to as ‘studies of those involved’ (point‐prevalence—now).
2. Where the presence‐absence was assessed after the individual has exited from terrorism, the studies were grouped together and referred to as ‘studies of those who have exited’ (period‐prevalence—after).
3. Where the presence‐absence of disorder or problems was assessed as being present before the individual becoming involved in terrorism, or before detection, then all studies were be grouped together and referred to as ‘disorder and problems prior to engagement or detection (period‐prevalence—before)’. For studies based on open‐source data, and where individuals in the data set came to the attention of researchers through their arrest, detention and prosecution, then the date of the index offence (i.e., detection) was taken as the point at which the individual become involved in terrorism. Where multiple pre‐involvement time points are taken in a study, then the team will decide on the most appropriate time point (or time points) to be include in the data syntheses, with justifications provided for all decisions made on a study‐by‐study basis.

To provide further context for our synthesis and discussion of findings, we refer to global estimates of point and period prevalence rates of disorder and psychological problems in the synthesis and discussion of findings.

In our protocol we had envisaged that there may be studies reporting levels of psychological problems based on validated measures of distress (e.g., the Depression, Anxiety and Stress Scale (DASS‐21); Lovibond & Lovibond, 1995). Had this been the case, our intention had been to meta‐analyse those studies where dimensional data was reported for those above a clinical cut‐off score on the measures (i.e. scores are suggestive of a clinical problem). However, there were insufficient eligible studies reporting this type of data to enable a data synthesis.

### 4.2 Search methods for identification of studies

Our search strategy aligned with Cochrane Training and our past reviews for both the Cochrane Collaboration (Doody et al., 2019) and the Campbell Collaboration (Carthy et al., 2020). It was also informed by Kugley et al. (2017) guidance on information retrieval for Campbell systematic reviews.

#### 4.2.1 Electronic searches

The Campbell Collaboration Crime and Justice Coordinating Editor and Information Specialist (Elizabeth Eggins) executed a systematic search of electronic listed in Table 2 in April 2022. These platforms and databases were selected as they provide coverage of journal articles across a range of publishers and disciplines, as well as indexing unpublished grey literature and academic theses.

**Table 2:** Search platforms and databases

Platform	Database
ProQuest	International Bibliography of the Social Sciences
ProQuest	Dissertations and Theses Global
ProQuest	Social Sciences Premium
OVID	PsycArticles
OVID	PsycExtra (grey literature)
OVID	PsycInfo
Embase.com	Embase
ISI Web of Science	Web of Science Core Collection^a^
	Social Sciences Citation Index (SSCI)—1956‐present
	Conference Proceedings Citation Index‐ Social Science & Humanities (CPCI‐SSH)—1996‐present
	Book Citation Index—Social Sciences & Humanities (BKCI‐SSH)—2005‐present
Scopus	
PubMed	
EBSCO	Criminal Justice Abstracts

a Note that Chemical Indexes, Current Chemical Reactions (CCR‐Expanded), Index Chemicus (IC) and Emerging Sources Citation Index (ESCI) are included in the institutional WoS Core Collection but were excluded from our search.

The search syntax was tailored for each search source, with a preference for searches based on the title, abstract keywords, and subject indexing fields. The full search record is provided in Supporting Information: Appendix A and captures research published until 31 December 2021. Titles and abstracts for all records captured in our search were be exported into EndNote for de‐duplication and the imported into *DistillerSR* reference management software for screening.

#### 4.2.2 Searching other resources

We anticipated that some relevant studies may be published as government reports or outputs from think‐tanks or other non‐governmental organisations. As such, they may not be indexed on electronic databases. To ensure these studies are identified, we (KS) searched the websites set out in Table 3 in April 2022. The Titles and Abstracts/Executive Summaries for all papers identified from this search were identified as ‘Grey Literature’ for reporting in our PRISMA chart.

**Table 3:** Grey literature searching

Organisation	Website
Radicalisation Awareness Network (EU)	https://ec.europa.eu/home-affairs/what-we-do/networks/radicalisation_awareness_network_en
Department of Homeland Security (US)	https://www.dhs.gov
Centre for Counter‐Terrorism Coordination (Australia)	https://www.homeaffairs.gov.au/about-us/our-portfolios/national-security/countering-extremism-and-terrorism/centre-for-counter-terrorism-coordination
Public Safety Canada	https://www.publicsafety.gc.ca/index-en.aspx
Home Office (UK)	https://www.gov.uk/government/organisations/home-office
Global Terrorism Research Centre	https://www.monash.edu/arts/social-sciences/gtrec
National Consortium for the Study of Terrorism and Responses to Terrorism (START)	https://www.start.umd.edu
Terrorism Research Centre	http://www.terrorism.org/
Hedayah	https://www.hedayahcenter.org

We (KS) directly contacted leading experts and expert networks (the Global Research Network on Terrorism and Technology, the European Expert Network on Terrorism issues (EENeT), VOX‐Pol Network of Excellence (NoE), the Radicalisation Awareness Network (RAN) and the Five Research and Development (5RD) Countering Violent Extremism Network.). Experts were advised of the review objectives as well as the specific type of literature sought for the synthesis. Titles, abstracts and full texts of papers identified from these sources were retrieved identified as ‘Experts’ for reporting in the PRISMA chart.

There can be a delay in indexing newly published journal articles. For this reason, we (KS) conducted a hand‐search in April 2022 of the following journals for papers published since January 2020:

- *Behavioural Sciences of Terrorism and Political Aggression*
- *Critical Studies on Terrorism*
- *Dynamics of Asymmetric Conflict*
- *Intelligence and Counter‐Intelligence*
- *International Journal of Conflict and Violence*
- *International Journal of Terrorism and Political Hotspots*
- *Journal of Deradicalization*
- *Journal of Policing, Intelligence and Counter‐Terrorism*
- *Journal of Terrorism Research*
- *Journal of Terrorism Studies*
- *Perspectives on Terrorism*
- *Science of Terrorism and Political Aggression*
- *Studies in Conflict and Terrorism*
- *Terrorism and Political Violence*

We (KS) also reviewed past systematic reviews in the area to identify papers relevant to our review (e.g., Gill et al., 2021). Citations and abstracts were identified as ‘Hand Search’ records for reporting in our PRISMA chart.

The bibliography sections of the papers included in the review were examined for literature that may meet our inclusion criteria (i.e., reverse citation chaining). We (KS, SC and KC) also searched for papers that cite these relevant articles and reports using the ‘citing articles’ function, where present, on search engines (i.e., forward citation chaining; Cribbin, 2011). For example, where a paper that met our inclusion criteria was indexed on SCOPUS, a ‘Citing articles’ ribbon on the website identified any publications indexed on the database that cited the target article and which subsequently screened. We also utilised Google Scholar for forward citation chaining. Articles identified through citation chaining were recorded as ‘Chaining’ records in our PRISMA chart. Finally, we completed a broad sweep of Google Scholar, focusing on titles, using search terms sensitive to our review objectives (i.e., intitle: Terrorism OR Extremism AND Mental).

### 4.3 Data collection and analysis

#### 4.3.1 Criteria for determination of independent findings

During the full‐text review process we identified a number of studies that shared the same datasets and, thus, raised concern in relation to the independence of the samples for the data synthesis. Where datasets are shared, we made decisions as to which studies to include in the data synthesis, with all studies being included in the narrative synthesis (see Sarma et al., 2022). Studies were flagged if:

1. One or more authors co‐authored multiple studies focusing on the same outcome (type of terrorism). In such instances, we contacted authors to determine whether the same samples were used in the studies.
2. It was clearly stated in a study that the sample was previously used in another included study.
3. It was clearly stated in a study that the study expanded upon a sample used in an earlier study.
4. A study used an open‐source publicly available data set (e.g., PRIUS or ECDB) which was potentially used by different author teams.

In prioritising studies for inclusion in the data synthesis, we adhered to a range of decision‐making rules

First, we prioritised larger samples on the basis that these contained the data in the studies with smaller samples. Typically, the larger datasets were also more recent publications, building on samples reported in earlier publications. For example, we excluded Simi et al. (2015) and Simi et al. (2016) from the data synthesis as the corresponding author confirmed that the data in those studies were expanded upon in their later paper (Bubolz & Simi, 2019) which was included. Similarly, three papers on lone actor terrorism (ideological mass shooters) by Capellan were eligible for inclusion (Capellan, 2015; Capellan & Anisin, 2018; Capellan et al., 2019). Capellan confirmed that 40 participants in the 2015 paper and 45 participants in the 2017 paper are included in their 2019 paper and the authors provided data for positive cases of diagnosed disorder and suspected problems based on the *n* = 47. We, therefore, excluded the two earlier papers from the data synthesis.

Second, where samples in the studies overlapped, but where there were unique samples in some studies, we included the unique samples as distinct studies and then prioritised the remaining samples based on sample size. As an example, in 2014, Gill et al. published their paper *Bombing alone* based on a data set of 119 lone‐actor terrorists from the USA and EU. This sample included lone‐actors who acted as individuals or as isolated dyads. In that paper, they report prevalence rates of mental illness for 11 isolated dyads, 87 individuals who acted alone without command and control links, and 21 individuals with command and control links. In 2015, Corner and Gill's paper, *A false dichotomy*, drew on the same sample and compared the rates of disorder to a sample of group‐based actors. The sample of group‐based actors is unique to papers from this team of researchers and so is included in the data synthesis.

The sample of lone‐actors is not unique as some are used in Gill's subsequent (2015) book (*Lone‐actor terrorist: A behavioural analysis*), and Corner et al.'s (2019) paper *The multi‐finality of vulnerability indicators in lone actor terrorism*. In their 2015 book, Gill drew on a sample of 111 lone‐actors and that included some of the 2014 lone‐actor sample but excluded ‘isolated dyads’ and individuals who acted alone ‘but in facilitative roles’ (p. 20). As such the 2014 *Bombing alone* paper and 2015 *Lone‐actor terrorist* lone‐actor samples are not unique. However, Gill is clear that they have omitted the ‘Isolated Dyads’ sample from the 2014 paper in this book (and thus also *A false dichotomy*) and that sub‐sample of 11 individuals is unique and is included in the data synthesis.

In 2019 Corner, Bouhana and Gill's paper *The multi‐finality of vulnerability indicators in lone actor terrorism*, took Gill's 111 lone‐actors and expanded this data set to 125 individuals. Clemmow et al. (2020) *Analysing person‐exposure patterns in lone‐actor terrorism* used the same 125 individuals. As the 125 individuals included in Corner et al. (2019) includes all of the Lone‐Actor Terrorist (2015) data, we excluded that book from the meta‐analysis. We also excluded Clemmow et al. (2020) in favour of the Corner et al. (2019) paper. The latter provided prevalence data for the full sample and the most complete data on mental health difficulties. We also excluded Gill et al. (2021) *Similar crimes, similar behaviours?* and Horgan and colleagues (2016) comparison of lone actors and mass murderers (*Across the Universe?*) from the data synthesis (though *Across the universe?* is considered under Objective 3 as it is a comparative study) as the data is included in *Bombing alone* and overlaps with the aforementioned included papers.

Third, where the same data set is used across multiple studies, we prioritised the study that most comprehensively dealt with mental health difficulties and terrorism. For example, Corner and Gill's 2020 paper, *Psychological distress, terrorist involvement and disengagement from terrorism*, is not focused on lone‐actor terrorism and presents a unique data set based on 91 autobiographies of terrorists or former terrorists. However, this is the same data set as their 2021 paper, *Psychological distress and terrorist engagement*. The 2020 paper is used in the data synthesis as it presented more comprehensive data on prevalence rates of mental illness/mental disorder. Similarly, Candilis et al. (2021) and Dhumad et al. (2020) used the same data set of 120 terrorist offenders (both lone‐actor and group‐based) who had been incarcerated in Bagdad, Iraq. We used the latter in the data synthesis as it contained more detail on the mental health difficulties of the sample.

Fourth, our results present separate analyses for diagnosed disorder, suspected disorder and psychological problems, as well as overall mental health difficulties. Where one study reports on psychological problems and another on diagnosed disorder, and drew on the same data set, then the issue of independence did not arise for the separate analyses (e.g., rates of disorder, rates of psychological problems, etc.). For example, Weenink produced two papers on Jihadists who travelled, or attempted to travel, to Syria. In their 2015 study of 140 Jihadists, the reported rate of a clinically diagnosed disorder in the sample is 6%. In their subsequent paper of 319 Jihadists, the reported rate of psychological problems is 28%. Here, Weenink notes that the increase from 6% in the earlier paper was due to changes in coding (i.e., for the latter, a formal diagnosis was not required). For this reason, we include the 2015 paper in our data synthesis of diagnosed disorder and the 2019 paper for data synthesis of psychological problems.

The issue of independence does arise where we analyse rates of any mental health difficulties in terrorist samples. Here, studies reporting rates of diagnosed disorder, suspected disorder or psychological problems were eligible for inclusion. To manage independence, we prioritised studies in the following order: (1) studies reporting psychological problems, (2) studies reporting suspected disorder, (3) studies reporting diagnosed disorder. This approach presents the most lenient assessment of mental health issues in terrorist samples.

Fifth, in our protocol we had envisaged that some studies may report on multiple measures of the link between any mental health difficulty and terrorism (e.g., multiple measurements of anxiety), which would have introduced an issue with the dependency of effect sizes within studies. This only arose where studies reported on both diagnosed disorder and levels of psychological problems, diagnosed disorder and suspected disorder, or suspected disorder and psychological problems (i.e., two measurements of mental health difficulties). Again, we prioritised measures in the following order: (1) psychological problems, (2) suspected disorder, (3) diagnosed disorder.

Sixth, four studies reporting on ten samples used the Profiles of Individual Radicalisation in the United States (PIRUS) data set, and multiple studies also used the US Extremist Crime Database (ECDB). In prioritising studies using PIRUS we used the decision‐making rules set out earlier (e.g., sample size, unique samples, comprehensiveness of the reporting, relevance to separate analyses). We applied the same rules of studies using the ECDB. We note that in the analyses that includes datasets from both PIRUS and ECDB, there is likely to be significant overlap in the samples. To be sensitive to non‐independence in these samples, we dropped one of the datasets, repeated the analyses and observed whether this influenced the pooled estimates (i.e., as part of our sensitivity analysis).

Two authors (KS and SC) assessed the independence of findings and sought the input of study authors where necessary. For example, decisions in relation to treatment of the Gill and colleagues' papers were recommended by KS and SC concurred. Paul Gill also concurred when asked to advise on treatment of the papers. We provide a detailed account of, and rationale for, our management of independence in Supporting Information: Appendix B, including handling of the large number of studies using the PIRUS and ECDB open‐source datasets.

#### 4.3.2 Selection of studies

##### Overview

The screening process for the review was common for all objectives, commencing with title and abstract screening and followed by a full‐text review for eligibility assessment for the syntheses relating to (a) prevalence, (b) temporality or (c) risk factors. Studies could be included in more than one of the syntheses. Details on the screening and study selection process are provided below.

We used *DistillerSR* with the support of the Campbell Collaboration Crime and Justice Coordination Group editorial team (Elizabeth Eggins), including automated de‐duplication, setting the parameters for *DistillerSR*, and importing the systematic search into *DistillerSR* for screening.

Two authors (KC and SC) conducted the screening and in making decisions as to which studies are relevant to each objective, we adopted the following rules:

1. For Objective 1 (Prevalence) we were interested in prevalence rates of mental health difficulties in terrorist populations. If the study measured prevalence of difficulties in a terrorist sample, and the design is cross‐sectional, cohort, or case‐control, and prevalence data can be drawn from the study, then we reviewed the paper for Objective 1.
2. For Objective 2 (Temporality) we were seeking studies that allowed us to establish the prevalence rates of difficulties and where these pre‐date involvement in terrorism or detection for the index offence.
3. For Objective 3 (Risk Factor) we were interested in studies that compared the mental health difficulties of those involved and not involved in terrorism, including studies that are and are not sensitive to temporal sequencing.

##### Title and abstract screening

We used the artificial intelligence (AI) active machine learning (AML) function embedded in *DistillerSR* for title/abstract screening. Each title/abstract was screened once by one of two review authors (KC and SC) and their screening decisions informed the *DistillerSR*'s reprioritisation of study records so they are presented in order based of most to least predicted relevance to the review. We also used a predictive reporting tool within *DistillerSR* that estimates the number of relevant records identified, and which allowed the team to set a ‘stopping point’ in the review process. We set this point at 95% based on advice from the Campbell Collaboration Crime and Justice Coordinating Group editorial team and based on previous reviews taking this approach (Eggins et al., in press, Windisch et al., 2022). Together the AI's prioritisation function and the target stopping point of 95% enabled the screeners to terminate the Title/Abstract screening process without having to review all records captured by the systematic search. At that point, iterative sets of 50 records were reviewed by the screeners until no new records were eligible for inclusion within those sets. All remaining unscreened records were excluded. SC and KC reached that termination point having screened 56,330 records.

Titles/abstracts were excluded based on the following criteria:

1. The record is not unique (i.e., not a duplicate of another paper already in the library that was not identified using the automated function) (Yes, No)
2. The record is an ineligible eligible document type (e.g., a book review) (Yes, No)
3. The study does not deal with mental health and terrorist behaviour (Yes, No, Unsure)

Records with an answer of ‘No’ to any of the above criteria were excluded. If the record was screened as ‘Yes’ or ‘Unsure’, it was included in our full‐text eligibility screening. Upon completion of this phase of the screening, the reviewers used the consistency checking function in *DistillerSR* to identify any false negative decisions. Any discrepancies were resolved through discussion and with the involvement of a third reviewer (KS) if necessary.

##### Full‐text screening

Records retained following title/abstract screening were subject to a full‐text review in *DistillerSR*. Two review authors, working independently, reviewed each document excluding documents based on the criteria listed in Table 4. Where a study was judged as unclear in terms of inclusion two reviewers (KC and SC) reached a final decision through discussion, involving a third reviewer (KS) when required. A third review author (KS) also searched for errata to included studies, and where present re‐assessed the eligibility of each study based on the inclusion/exclusion criteria.

**Table 4:** Full‐text eligibility screening

*Eligibility General*		
1	Is the record unique (i.e., not a duplicate)?	1‐ No (exclude)
	*Documents which match another title exactly (i.e., title, author, year, and document type) should be excluded*.	2‐ Yes
2	Is the document published in English?	1‐ No (excluded)
	*Document not published in English should be excluded*.	2‐ Yes
3	Does the document report the results of an empirical study?	1‐ No (excluded)
	*Documents that do not report empirical findings (e.g., conceptual papers, newspaper articles and minutes of meetings) should be excluded*.	2‐ Yes
4	Does the study include a sample (or subsample) of individuals involved in terrorism as defined in the protocol?	1‐ No (excluded)
		2‐ Yes (move to next question)
*Eligibility Objective 1 Prevalence*		
5	Does the study measure the presence of mental health difficulties (as defined in the protocol) in the terrorist sample or subsample?	1‐ No (excluded)
		2‐ Yes (included for Objective 1, move to next question to consider Objective 2 and 3)
*Eligibility Objective 2 Temporality*		
6	Does the study establish that the mental health difficulties preceded the terrorist behaviour?	1‐ No (excluded for Objective 2)
		2‐ Yes (included for Objective 2, move onto next question to consider eligibility for Objective 3)
*Eligibility Objective 3 Risk Factor*		
7	Does the study include a sample where some individuals were involved in terrorism and others were not?	1‐ No (excluded for Objective 3)
		2‐ Yes (move to next question)
8	Does the study measure the presence of mental health difficulties, for both groups, as defined in the protocol?	1‐ No (excluded for Objective 3)
		2‐ Yes (move to next question)
9	Does the study report the relationship between the absence/presence of mental health difficulties and involvement in terrorism (involved/not involved), or provide sufficient information to calculate that relationship?	1‐ No (excluded for Objective 3)
		2‐ Yes

#### 4.3.3 Data extraction and management

For each eligible study, one review author extracted key information including study authors, design, population (e.g., convicted offenders etc.), data source, data type, diagnostic approach, diagnoses, symptoms, ‘type’ of terrorism, ‘role’ of terrorist, comparison group, and summary results. This data was recorded in detailed data extraction tables (see Supporting Information: Appendix C: Full‐Text Coding Form).

#### 4.3.4 Assessment of risk of bias in included studies

We used the *Joanna Briggs Institute Checklist for Prevalence Data* for all studies under Objective 1 and 2, and then augmented the checklist with the *JBI Checklist* for *Case Control Studies* for studies included under Objective 3. Items are presented in the Full‐Text Coding Form at Supporting Information: Appendix C and assessments were informed by explanatory guides for the tools (e.g., Munn et al., 2014).

The risk of bias for each study was interpreted as follows:

- Low risk of bias: All items are rated as ‘Yes’
- High risk of bias: At least one item rated as ‘No’.
- Unclear risk of bias: One item is listed as ‘unclear’ and the remainder as ‘Yes’.

We have added an additional Risk of Bias item to the JBI checklists—one that is sensitive to plausibility as discussed earlier in this protocol. Where studies present a theoretical argument linking mental health difficulties with terrorist involvement the quality criterion will be indicated as having been met. Our Risk of Bias discussion will consider these theoretical arguments with regard to the 4Ps model.

The risk of bias assessment was conducted by two independent coders and reliability was tested using the Kappa statistic *κ*. In line with Campbell Collaboration practice and policies, studies were not excluded based on their risk of bias. Instead, all studies for which effect sizes were obtained were included in the meta‐analyses. In reporting the results of the risk of bias assessment, we did so by clustering studies by design and reporting the ratings for each domain across all studies assessed in tables, accompanied by a written summary and rationale for our ratings.

#### 4.3.5 Assessment of heterogeneity

Heterogeneity was explored statistically using the homogeneity Q‐statistic and the *I* ^2^ test. Tau square was also reported along with each mean effect size.

#### 4.3.6 Assessment of reporting biases

Non‐significant results are less likely to be published and/or made broadly available for syntheses of this nature (Joober et al., 2012). As such, there may be an increased risk of reporting biases in the identified literature. To determine if the synthesised data was subject to such a publication bias, a contour enhanced funnel plot (Palmer et al., 2008) Trim and Fill test (Duval, 2005) and Egger's regression test (Egger et al., 1997) were used.

#### 4.3.7 Dealing with missing data

Where data needed for our analysis are missing from a paper, we contacted the corresponding authors, or other authors where the corresponding author does not respond, seeking access to additional data. Where this was not available then that paper was excluded from the meta‐analysis but retained for the narrative summary of eligible studies.

In our data synthesis we used the positive cases as a proportion of all cases in the data set for each study. Where studies reported valid percent (i.e., positive cases as a proportion of known negative cases and known positive cases, and excluding unknowns/missing) we recalculated the proportion with the full sample in the denominator. We discuss this further in the ‘Discussion’ section.

#### 4.3.8 Effect size calculation and data synthesis

Data synthesis was be completed using Comprehensive Meta‐Analysis (CMA) software (Borenstein et al., 2013).

For Objective 1 (Prevalence), proportion data (i.e., proportion of the terrorist sample with a mental health difficulty) were transformed into a logit for performing the meta‐analysis. The meta‐analysis assumed a random‐effects model a priori and used the REML estimator of the random‐effects variance component. Mean effect sizes and associated 95% confidence intervals were back‐transformed into proportions (prevalence) for ease of interpretation. Where studies reported rates of mental health difficulties before involvement or detection alongside life‐time rates that included the period after engagement and detection, we included rates calculated before engagement/detection, considering these to be most salient to our exploration of the mental health—terrorism hypothesis.

For Objective 1 we also draw on published global point and life‐time prevalence of mental health difficulties to provide a benchmark against which the prevalence rates can be assessed. We use these benchmarks in discussing our findings in the Discussion section of the review.

For Objective 2 (Temporality) we replicated this analysis including only those papers where authors asserted that the mental health difficulties pre‐dated involvement in terrorism or the index offence.

Finally for Objective 3 (Risk Factor) our intention was to calculate odds ratio for the presence/absence of mental health difficulties between those involved and not involved in terrorism. However, for reasons explained later, the studies were not suitable for calculating pooled estimates and instead we present a narrative synthesis for this objective.

#### 4.3.9 Subgroup analysis and investigation of heterogeneity

In line with the Cochrane Handbook (Deeks et al., 2019) where the meta‐analysis contained at least 10 studies we conducted further sub‐group analysis (see also Richardson et al., 2019).

In our protocol we proposed that it may be possible to conduct moderator analysis based on ‘Type’ of terrorism (Lone actor; Islamist; Right‐Wing; Separatist; Mixed; Other), onset of disorder/problems relative to becoming involved in terrorism (pre, during, post, mixed, not‐clear), developmental age of onset of difficulties (e.g., child/adolescent <18 vs. adult 18+), developmental age of involvement in terrorism (e.g., e.g., child/adolescent <18 vs. adult 18+), type of design (retrospective vs. prospective), the time period of interest (e.g., last 5 years, 2 years, 12 months, etc.) and source of data (open source, closed source, interview etc.). However, having extracted data for the eligible studies it was clear that there were insufficient studies to complete sub‐group analysis by age of onset of difficulties and developmental age of involvement in terrorism, and that less granular sub‐group analysis was appropriate for data source and type of terrorism. In the review, then we report on two moderator analyses—(a) for type of terrorism, categorised as lone actor versus other and (b) data source, where we compare studies that used either closed sources or interviews and with those that used open sources.

#### 4.3.10 Sensitivity analysis

As mentioned, studies were not excluded based on their risk of bias. We ran a series of meta‐analyses to explore the effect of excluding studies, based on theoretical reasoning, and including where we assessed that specific studies may present a high risk of bias. The purpose of this additional step was to formally explore if the observed effect sizes were dependent on the inclusion of studies.

#### 4.3.11 Treatment of qualitative research

Qualitative research was excluded from the synthesis unless reported as part of a mixed‐methods study where quantitative data was also reported. In such cases, the quantitative data was synthesised and the qualitative content reviewed and used to contextualise the findings.

### 4.4 Deviations from the protocol

There were three deviations from the protocol. First, in our protocol we stressed the importance of distinguishing between point and period prevalence, and different points and periods, when reporting and pooling prevalence rates. For example, we envisaged that studies would report life‐time, 12‐ and 24‐month prevalence rates (i.e., different periods) and at different points (i.e., right now vs. at time of engagement vs. at time of disengagement, etc.). However, of those studies reporting period prevalence, all focused on life‐time prevalence rates of mental health difficulties. For point prevalence, eligible studies focused on difficulties present at the time of an attack (‘detection’), while being processed through the courts, or while incarcerated. Thus, the review reports on a more restricted set of points and periods than initially envisaged in the protocol.

Second, Objective 2 deals with temporal sequencing and we had initially envisaged isolating studies that reported on rates of difficulties that were onset before the involvement in terrorist behaviour. Such evidence, we stressed, places the hypothesised risk factor in the correct temporal sequence to the outcome and provided a purer test of the mental health‐terrorism hypothesis. However, studies that dealt with temporal sequencing, with one exception, focused on the presence of difficulties before detection rather than involvement—the limitation being that it is possible that such difficulties were onset between the point of engagement and their detection. The exception is Corner and Gill (2020), with Corner providing the authors with data on mental health difficulties before engagement.

Third, we had anticipated differentiating between diagnosed disorders and psychological problems. However, when we extracted the data, it became clear that studies had adopted a number of different approaches to coding for diagnosed disorders and which set more or less stringent thresholds for assessment and diagnosis. To be sensitive to this we differentiate between ‘diagnosed disorders’, ‘suspected disorders’ and ‘psychological problems’ as follows:

1. If a study referred to mental disorder or mental illness and where all individuals in the sample have a ‘confirmed diagnosis’, ‘clinical diagnosis’, ‘formal diagnosis’, or diagnosis made by a trained professional (or within a mental health report), then it was coded as relating to ‘mental disorder’. As examples, Bubolz and Simi (2019) refer to mental disorder as being present where ‘a medical practitioner had ever diagnosed the person’ (p. 6), Capellan (2015) refers to ‘formal’ and ‘confirmed diagnosis’ (p. 400) and Corner and Gill (2021) required an ‘official psychiatric consultation or diagnosis’ (p. 705).
2. If a study referred to mental disorder or mental illness but individuals (or a proportion of the individuals) but a formal diagnosis was not required, or where a diagnosis is suspected based on reported symptoms and the alignment of these symptoms with DSM or ICD criteria, then it was coded as relating to ‘suspected mental disorder’. For example, some studies referred to disorders being present when either (a) there was a formal clinical diagnosis or (b) publicly available information suggested that they met the criteria for a mental disorder but as assessed based on that information only. For example, Corner and Gill (2019) relied on open‐sources when classifying their sample as having a mental disorder. Disorder was present where there was an official diagnosis, but also where researchers formed the opinion that reported symptoms aligned with DSM/ICD criteria.
3. Finally, where studies referred to disorder but where there was no confirmed diagnosis, or no reference to diagnostic systems, the studies were coded as psychological problems. Some studies explicitly sought to explore psychological difficulties, distress, disturbance, etc., rather than disorder, and such studies were also clustered as reporting psychological problems. Capellan and Anisin (2018), for example, coded individuals as having a mental disorder when there was a confirmed diagnosis, or based on ‘characteristics by family members and close friends [that suggest] mental disturbance’ (p. 244).

Table 5 sets out our interpretation of the nature of the mental health difficulties being reported in each paper, with an accompanying justification.

**Table 5:** Classification of risks being examined in each included study.

Study	Rationale for mental health category
Bakker (2006)	Refers specifically to mental illness at the time of assessment and base rate in community ‘eleven of them suffer from mental illness’ (p. 40)… However, no formal diagnosis confirmed in text. So suspects disorder, but no diagnosis. Coded as Suspected Disorder—Current.
Bergen et al. (2017)	Refers to ‘Diagnosed with a mental health issue or were credibly reported to be suffering from a mental health issue’ (p. 3). Coded as Suspected disorder but not clear if point‐prevalence (now) or 12‐month period prevalence. Further information sought from authors.
Böckler et al. (2018)	Refers to mental disorders and mental dispositions so coded as Psychological Problems—Lifetime. Further information sought from authors.
Bronsard et al. (2022)	Refers to formal clinical assessment. Coded as Diagnosed Disorder—Current.
Brym and Araj (2012)	Refers to ‘outwards signs of depression… or personal crisis’, so coded as psychological problems—lifetime. Further information sought from authors.
Bubolz and Simi (2019)	Refers specifically to a mental disorder diagnosed by a medical practitioner (‘a medical practitioner had ever diagnosed the person with a mental health problem’ p. 6). However, they include evidence of maladjusted behaviour in their cases so we coded as Psychological Problems ‐ Lifetime Prevalence.
Candilis et al. (2021)	Refers to assessment of conduct disorder and anti‐social personality disorder by 4 trained academic psychiatrists. This is same sample as Dhumad et al., 2020 who note that those who completed the interviews were trained in all tools (p. 76). Therefore we coded as Diagnosed Disorder—Life‐time Prevalence
Capellan (2015)	Refers to ‘formal’ and ‘confirmed diagnosis’ (p. 400). Separately reports data for psychological problems. So we coded as a) Diagnosed Disorder Life‐Time and b) Psychological Problems Lifetime
Capellan and Anisin (2018)	This is based on open‐source data and where diagnosis was confirmed or where it is suggested based on ‘characteristics by family members and close friends [that suggest] mental disturbance’ (p. 244). Mental disturbance conceptualised as ‘adverse psychological processes’ (p.244). As no claim this is about diagnosed disorder, or suspected disorder, we classified study as being about Psychological Problems.
Capellan et al. (2019)	Refers to ‘confirmed diagnosed mental illness’ (p. 105) and separately suspected problems and so coded here as (a) Diagnosed Disorder—Life Time and (b) Psychological Problems ‐ Lifetime
Chermak and Gruenewald (2015)	Refers to ‘known diagnosed mental illness’ (p. 142) and therefore coded as Diagnosed Disorder ‐ Lifetime
Cherney et al. (2022)	Refers to ‘confirmed diagnosed mental illness’ (p. 105) and so coded here as Diagnosed Disorder—Lifetime
Clemmow et al. (2020)	Used data set reported in Corner et al. (2019). In communication with Corner and Gill based on open source data only and not always clinical diagnosis, but reference to DSM/ICD criteria by researchers. So we classified as Suspected Disorder.
Corner and Gill (2015)	Communication from Gill and Corner states that the team used the ICD to code some cases for disorder based on symptoms present and hence coded as Suspected Disorder—Lifetime.
Corner et al. (2019)	In communication with Corner and Gill, suspected diagnosis as based on open source data only and not always clinical diagnosis, but reference to DSM/ICD criteria by researchers. Also reports psychological distress. So coded as Suspected Disorder and Psychological Problems—Lifetime.
Corner and Gill (2020)	Paul Gill confirmed that this paper uses same data set as Corner and Gill (2021) and Corner confirmed this is based on diagnosed disorder, and with the input of psychiatry in the research coding process. Also reports psychological distress. Classified as (a) Diagnosed Disorder Life‐Time and (b) Psychological Problems Life‐Time.
Corner and Gil (2021)	Reports that ‘official psychiatric consultation or diagnosis’ (p.705) required and if not, coded as ‘psychological distress’ (psychological problems for the purpose of this review. Authors contacted and confirmed psychiatrists aided coding of this field. Hence coded as (a) Diagnosed Disorder Lifetime and (b) Psychological Problems Lifetime.
de Roy van Zuijdewijn and Bakker (2016)	Refers to ‘indicators’ (p. 44) and ‘suggestion’ (p. 43) of mental health disorder in open‐sources used to build the data set and hence coded as Suspected Disorder Lifetime
Dhumad et al. (2020)	Refers to diagnostic system and staff trained in the interview tools. Hence coded as Diagnosed Disorder Life‐Time
Duits et al. (2022)	Refers to ‘Mental disorders and its traits and symptoms were based on the extensive forensic mental health assessments’ (p. 5) and provides data for disorders and symptoms. Hence coded as Diagnosed Disorder Life‐Time and Psychological Problems Life‐Time.
FBI (2019)	Refers to sample who ‘were formally diagnosed with one or more psychiatric disorders at some point before their attack’ (p. 20). Also refers other analysis where ‘behaviors or symptoms that could have been indicative of mental health stressors’ (p. 24). Hence study provides data for (a) Diagnosed Disorder—Lifetime and (b) Psychological Problems Lifetime
Freilich et al. (2019)	Refers to ‘history of diagnosed mental illness’ (p. 949). Also reports psychological problems. Coded here as (a) Diagnosed Disorder Lifetime and (b) Psychological Problems Lifetime.
Gibson (2018)	Provides examples in case studies that suggest focus is on diagnosed disorder or suggested disorder. Coded as Suspected Disorder—Lifetime
Gill et al. (2014)	They coded disorder based on (a) confirmed diagnosis, or (b) compatibility with ICD. So some not formally diagnosed. Hence coded as Suspected Disorder Lifetime
Gill (2015)	Suspected disorder as no confirmed diagnosis but reference to diagnostic systems. Coded as Suspected disorder—Lifetime
Gill et al. (2021)	The lone‐actors in this data set is same as Gill et al. (2014). They coded disorder based on (a) confirmed diagnosis, or (b) compatibility with ICD. So some not formally diagnosed. Hence coded as Suspected Disorder Lifetime
Gill et al. (2022)	Refers to ‘diagnosis had been made’ (p. 118) and based on closed‐sources, so coded as Diagnosed Disorder Lifetime
Gottschalk and Gottschalk (2004)	Refers to ‘psychopathology’, uses MMPI for assessment and refers to clinical thresholds. Coded as Diagnosed Disorder—Current. Further information sought from authors.
Gruenewald et al. (2013)	Refers to ‘mental health issue’ (p.77) and ‘mental illness’ but does not refer to ‘diagnosed’ or ‘confirmed’. Coded as Suspected Disorder—Life Time
Hamm et al. (2017)	Refers to ‘we are primarily relating on court documents, psychiatric evaluations and news coverage. In some cases mental health problems were not clinically diagnosed’. Coded as Psychological Problems ‐ Lifetime
Haugstvedt and Koehler (2021)	Authors confirmed that not all cases involved diagnosis, and inferences made based on information in open‐sources. Coded as Psychological Problems—Lifetime.
Horgan (2016)	This is the same data set as Gill et al. (2021) and a sub‐sample of Gill et al. (2014); and Corner et al. (2019). Coded as Suspected Disorder as they refer to mental illness and ICD 10 codes—Suspected Disorder—Lifetime.
Khazaeli and Khoshnood (2019)	Refers to ‘confirmed diagnosed mental illness’ (p. 105) and so coded here as Diagnosed Disorder—Lifetime
Jensen and Kane (2021)	Refers to ‘mental health concerns’ (p. 10) and coded as Psychological Problems—Lifetime
Kupper and Meloy (2021)	Refers to limitations of using manifestos to determine diagnosed disorders and so coded as Psychological Problems—Lifetime
LaFree et al. (2018)	Presents information on ‘clinical diagnosis’ and on psychological problems as reported by self‐report and ‘testimony by family and friends’ (p. 246). Coded as (a) Diagnosed Disorder—Lifetime and (b) Psychological problems—Lifetime
LaMontagne (2019)	Refers public/popular speculation and professional diagnosis and not reported separately (‘ordinal: 0 = No; 1 = Yes, according to public/popular speculation and 2 = Yes, professionally diagnosed’ (p. 59). Acceptance of public/popular speculation for classification, so we coded as Psychological Problems—Lifetime.
Lankford (2013)	Refers to personal crises which may include psychological problems (lifetime rates). Contacted authors requesting data.
Liem et al. (2018)	Refers to ‘official diagnosis’ and ‘indication of a mental disorder’ and presents separate data for each. Thus coded here as (a) Diagnosed Disorder–Lifetime and (b) Psychological Problems—Lifetime.
Lucas (2021)	Refers to ‘evidence of a mental illness’(p. 22) and later ‘some’ evidence of mental illness. No reference to diagnostic criteria, so coded as Psychological Problems: Lifetime
Lyons and Harbinson (1986)	Based on clinical interviews with trained mental health professionals with subjects mental health assessed at time of prosecution for offence. Coded as Diagnosed Disorder—Current.
Merari and Ganor (2020)	Based on clinical interviews with trained professionals in prison setting. Coded as Diagnosed Disorder—Current.
Merari et al. (2010)	Refers to ‘depressive tendencies’ (p. 95) and then diagnosed disorder during clinical interview—Coded as diagnosed disorder and psychological problems—Current
Merari (2021)	Contains data from both Merari and Ganor (2020) and Merari et al. (2010) and so reports diagnosed disorder and psychological problems—Current
Meloy et al. (2019)	Corresponding author confirmed that not all terrorist sample had a formal diagnosis. Coded as Suspected Disorder—Lifetime
Perry et al. (2018)	Reports ‘indications of mental illness’ (p. 908) and thus coded as Psychological Problems—Lifetime
Pfundmair et al. (2022)	Refers only to ‘mental problems’ and coded as Psychological Problems—Lifetime
Pitcavage (2015)	Refers to ‘tentatively, that 7 of the 35 perpetrators (20%) may have suffered from a degree of mental illness’. Opensource data and no suggestion of diagnosis. Coded as Psychological Problems—Lifetime
Pendergast (2020)	Refers to ‘mental health is a dichotomous measure where an extremist either has no known previous history of mental health issues, or there is some evidence of past mental health’ (p. 41). No suggestion of diagnosis, so coded as Psychological Problems—Lifetime
Simi et al. (2015)	Same sample as Bulboz and Simi (2019)
Simi et al. (2016)	Same sample as Bulboz and Simi (2019)
Thijssen et al. (2021)	Refers to psychiatric reports and thus coded as Diagnosed Disorder—Lifetime
van Leyenhorst and Andreas (2017)	Reports that ‘files showed four suspects who were, prior to their current prosecution, diagnosed with a DSM‐IV disorder’ (p. 332). Coded as Diagnosed Disorder—Lifetime
Weenink (2019)	Refers to mental health problems that were clearly present or indications that they were present. Coded as Psychological Problems—Lifetime
Weenink (2015)	Refers to ‘They have been clinically diagnosed with a disorder’ (p. 21). Coded as Diagnosed Disorder—Lifetime
Zeman et al. (2018)	Refers to ‘diagnosed’ and reference to ‘mental health examination’ on p. 15. Coded as Diagnosed Disorder‐Lifetime

## 5 RESULTS

### 5.1 Description of studies

**Figure 1:**
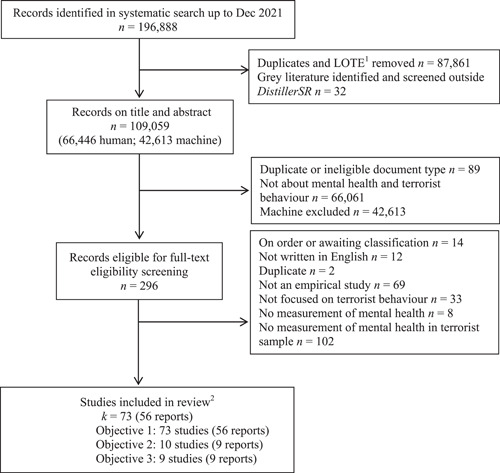
PRISMA flow diagram. ^1^LOTE = Language other than English. ^2^Studies could be included under more than one review Objective and so studies listed by Objective will not sum to total studies included in the review.

Figure 1 (PRISMA flowchart) presents the results of our search, screening and identification process for all review objectives. The systematic search of electronic databases identified 196,888 records, unrestricted by publication date. After de‐duplication, 109,027 records were progressed to title/abstract screening in *DistillerSR*. An initial stage of de‐duplication was conducted in EndNote, followed by another stage of de‐duplication using the Detect Duplicates function in *DistillerSR* (which can identify additional duplicates compared to EndNote). A total of 108,763 records were excluded at this stage as duplicates, ineligible types (e.g., book reviews), or because they were not about terrorism and mental disorder, leaving 264 records for full‐text review.

The full‐texts of all records were obtained and screened in *DistillerSR*. These were combined with full‐texts of records harvested from past reviews, the grey literature, hand searches of journals, forward citation searching, and consultation with expert groups (*n* = 32). Of the 296 documents, the full text of 14 could not be sourced and are listed in the ‘Studies awaiting classification’ reference section. The remaining full texts were reviewed, with two excluded due to being duplicates of other studies. Others were ineligible as they were not published in English (*n* = 12), were not focused on terrorist behaviour (*n* = 33), were not empirical studies (*n* = 69), did not include a measure of mental health (*n* = 8), or did not measure mental health in a terrorist sample (*n* = 102). This left 56 papers reporting on 73 terrorist samples (studies). For clarity, henceforth we use the term ‘paper’ to refer to the original full‐text record and ‘study’ to refer to each unique terrorist sample isolated.

All 56 papers, reporting 73 studies, were included in Objective 1 (Prevalence), though not all were included in the data syntheses reported due to overlapping datasets. Of these, 9 papers (10 studies) reported prevalence rates where the difficulties were onset before either terrorist involvement or the index offence and were eligible for Objective 2 (Temporality). Finally, 9 papers were eligible for synthesis under Objective 3 as they examined the association between mental health difficulties and terrorist behaviour (i.e., comparing terrorists and non‐terrorists). In the remainder of this section, we deal with each objective in turn.

### 5.2 Objective 1—Prevalence

#### 5.2.1 Included studies

##### Life‐time prevalence of mental health difficulties

Fifty‐six papers met our eligibility criteria for Objective 1 reporting on 73 studies (*n* = 13648). The characteristics of the studies are summarised in Table 6. The ethnicity of the samples, to the extent reported in the included studies, is summarised in Table 7. Of these studies, we have sought additional information for five (Bergen et al., 2017; Böckler et al., 2018; Brym & Araj, 2012; Gottschalk & Gottschalk, 2004; Lankford, 2013) to enable us to complete an assessment of risk of bias and extract data for our data synthesis. These papers are included only in our narrative syntheses.

**Table 6:** Characteristics of included studies for Objective 1—Prevalence

Study	Sample(s)	Paper	Design	Year data coverage	Risk	Prevalence	Source	Location(s)	Sample size	Age	Gender (Male%)	Ethnicity
Bakker (2006)	Jihadi	R	CS	2001–2006	S	C	OS	E	242	27.30	98	Unclear
Bergen et al. (2017)^a^	Jihadi	R	C	2001–2017	S	?	?	US	415	29	NR	Sample includes ‘white, African‐American, Somali‐American, Vietnamese‐American, Bosnian‐American, and Arab‐American’ (p. 34‐35)
Böckler et al. (2018)^a^	Jihadi	J	C	2000–2013	P	?	OS	Germany	7	23	100	Unclear
Bronsard et al. (2022)	Jihadi	J	CC	NA	D	C	Clin	France	15	17	60	Unclear
Brym and Araj (2012)^a^	Group actor	J	C	NR	P	LT	I	Palestine	43	NR	NR	Unclear
Bubolz and Simi, 2019	Right‐Wing	J	C	NR	P	LT	I	US	44	NR	86.4	Unclear
Candilis et al. (2021)	Mixed ‐ Iraqi	J	C	NR	D	LT	I	Iraq	160	34.10	100	Unclear
Capellan (2015)	Lone‐actor	J	CC	1970–2014	D & P	LT	OS	US	40	38.00	97.5	Non‐ideological active shooters: White = 59%; Black = 25%; Latino = 9%; Asian = 4%; Arab = 0.8%; Mixed = 1.3%. Ideological active shooters: White = 70%; Black = 18%; Latino = 0%; Asian = 3%; Arab = 10%; Mixed = 0%.
Capellan and Anisin (2018)	Lone‐actor	J	CC	1985–2015	P	LT	OS	US	45	NR	95.2	Unclear
Capellan et al. (2019)	Lone‐actor	J	CC	1966–2017	D & P	LT	OS	US	47^b^	37.7	97.8	‘Disgruntled’ offenders: White = 52%, Black = 33%, Hispanic = 11%, Asian = 3%, Middle Eastern= 0%, Other = 2%. ‘School’ offenders: White = 77%; Black = 8%; Hispanic = 3%; Asian = 8%; Middle Eastern = 0%; Other = 3%. ‘Ideological’: White = 57%; Black = 28%; Hispanic = 0%; Asian = 0%; Middle Eastern = 15%; Other = 0%. ‘Rampage’ offenders: White = 54%; Black = 23%, Hispanic = 15%; Asian = 5%; Middle Eastern = 3%, Other = 0.8%.
Chermak and Gruenewald (2015)	1. Right‐Wing	J	C	1990–2011	D	LT	OS: ECDB	US	637	28.40	92.6	White = 83.7%; Black = 8.8%; Hispanic = 1%, Arab = 5.3%; Other = 1.2%
	2. Left‐Wing	||	||	||	||	||	||	||	182	28.40	69.5	
	3. Jihadi	||	||	||	||	||	||	||	155	31.20	99.4	
	4. Mixed	||	||	||	||	||	||	||	974	28.90	89.5	
Cherney et al. (2022)	Mixed	J	C	1985–2020	D	LT	OS: PIRA	Au	33	17.00	90.9	Unclear
Clemmow et al. (2020)	Lone‐actor	J	C	1990–2015	S &P	LT	OS	US, E, Au	125	NR	NR	Unclear
Corner and Gill (2015)	Lone‐actor	J	C	1990–	S	LT	OS	US & E	119	NR	NR	Unclear
	Group Actor	||	||	NR	||	||	||	US/non‐US	119	NR	NR	
Corner et al. (2019)	Lone‐actor	J	C	1990–2015	S & P	LT	OS	US, E, Au	125	NR	NR	Unclear
Corner and Gill (2020)	Mixed	J	C	1900–	D & P	LT	OS: Auto	Global	91	NR	NR	Unclear
Corner and Gill (2021)	Mixed	J	C	1900–	D & P	LT	OS: Auto	Global	91	22.2.	70.6	Unclear
de Roy van Zuijdewijn and Bakker (2016)	Lone‐actor	J	C	2000–2014	S	LT	OS	E	120	29.70	NR	Unclear
Dhumad et al. (2020)	Mixed‐Iraqi	J	CC	NR	D	LT	I	Iraq	160	34.06	100	Unclear
Duits et al. (2022)	1. Jihadi ‐ <22	J	C	2012–2021	D & P	LT	CS: EDT	E	46	19.00	85	Unclear
	2. Jihadi ‐ 22+	||	||	||	||	||	||	||	120	29.00	96	
FBI (2019)	Lone‐actor	R	C	1972–2015	D & P	LT	CS + OS	US	52	37.70	100	White = 65%; Middle Eastern = 13%; Black = 8%; Bi‐racial = 8%, Asian = 4%, Hispanic = 2%
Freilich et al. (2019)	Mixed	J	C	1990–2013	D & P	LT	OS: ECDB	US	447	NR	NR	Unclear
Gibson (2018)	Jihadi	T	C	2001–	S	LT	OS	US	38	22.4	86.8	Unclear
Gill et al. (2014)^c^	1. Lone‐actors ‐ No links	J	C	1990–	S	LT	OS	US, E	87	33	97	Unclear
	2. Lone‐actors ‐ Links	||	||	||	||	||	||	||	21	33	97	
	3. Lone‐actor ‐ Dyads	||	||	||	||	||	||	||	11	33	97	
Gill (2015)	Lone‐actor	B	C	1990–	S	LT	OS	US, E	111	33	92	Unclear
Gill et al. (2021)	Lone‐actor	J	C	1990–2014	S	LT	OS	US, E	71	34	NR	Unclear
Gill et al. (2022)	Lone‐actor	J	C	1995–2015	D	LT	OS	UK	49	NR	87.8	Unclear
Gottschalk and Gottschalk (2004)^a^	Mixed	J	C	NR	D	C	I	Palestine & Israel	90	NR	NR	Unclear
Gruenewald et al. (2013)	1. Lone‐actor ‐ Right‐Wing	J	C	1990–2010	S	LT	OS: ECDB	US	47	35.30	100	Unclear
	2. Group Actor ‐ Right‐Wing	||	||	||	||	||	||	||	92	27.20	96.7	
Hamm et al. (2017)	Lone‐actor	B	C	1940–2016	P	LT	OS	US	108	NR	95.4	Sample ethnicity was described as ‘predominantly white (64%)’ (p. 50).
Haugstvedt and Koehler (2021)^d^	Right Wing	J	C	1948–2019	P	LT	OS: PRIUS	US	526	NR	NR	Unclear
Horgan (2016)	Lone‐actor	R	CC	1990–2014	S	LT	OS	US	71	34	98.6	Unclear
Khazaeli and Khoshnood (2019)	Lone‐actor	J	C	2015–2016	D	LT	OS	E	36	NR	97.2	Unclear
Kupper and Meloy (2021)	Mixed	J	CC	1974–2021	P	LT	OS: Manif	US, Ca, EU, NZ, Finland	22	NR	NR	Unclear
Jensen and Kane (2021)	1. Mixed	J	C	NR	P	LT	OS: PRIUS	US	1715	35.40	89.9	Unclear
	2. QAnon	||	C	To 2021	P	LT	OS: PRIUS QAnon	US	51	41.60	72.7	
LaFree et al. (2018)	Mixed	J	C	1948–2013	D & P	LT	OS: PRIUS	US	1473	34.20	90	Unclear
Lankford (2013)^a^	Suicide attackers	J	C	1990–2010	?	LT	OS	US	12	41.42	100	Unclear
LaMontagne (2019)^c^	1. Jihadi	T	C	1948–2016	P	LT	OS: PRIUS	US	455	NR	91	White = 65%; Black = 15%; Middle Eastern/North African = 9%; Hispanic/Latino = 5%; Asian = 4%; Native American = 0.04%.
	2. Left‐Wing	||	C	||	||	||	||	||	324	NR	91	
	3. Single‐Issue	||	C	||	||	||	||	||	340	NR	91	
	4. Right‐Wing	||	C	||	||	||	||	||	746	NR	91	
Liem et al. (2018)	Lone‐actor	J	CC	2010–2016	D & P	LT	OS	E	98	30.30	96	Unclear
Lucas (2021)	Right Wing Suicide	T	C	1990–2020	P	LT	OS: ECDB	US	16	NR	NR	Unclear
	Right Wing Suicide	T	||	1990–2020	P	LT	OS: DTGrwx	Germany	12	NR	NR	
Lyons and Harbison (1986)	Group Actor	J	CC	1974–1984	D	C	Clin	NI	47	32.00	NR	Unclear
Merari and Ganor (2020)	Lone‐actor	J	CS	NR	D	C	Clin	Israel & Palestine	39	NR	86.7	Unclear
Merari et al. (2010)	Group actor – non‐suicide	J	CS	NR	D & P	C	Clin	Israel & Palestine	12	19.6	100	Unclear
	Group actor ‐ Suicide	||	||	NR	D & P	C	Clin	Israel & Palestine	15	19.80	100	
	Group actor ‐ Organiser	||	||	NR	D & P	C	Clin	Israel & Palestine	14	27.60	100	
Merari (2021)	Group actor – non‐suicide	BC	CS	NR	D & P	C	Clin	Israel & Palestine	12	19.6	100	Unclear
	Group actor ‐ Suicide	BC	||	NR	D & P	C	Clin	Israel & Palestine	15	19.8	100	
	Group actor ‐ Organiser	BC	||	NR	D & P	C	Clin	Israel & Palestine	14	27.6	100	
Meloy et al. (2019)	Lone‐actor	J	C	1993–2016	S	LT	OS	US	33	39.00	100	Unclear
Perry et al. (2018)	Lone‐actor	J	C	2000–2016	P	LT	CS + OS	Israel & Palestine	62	NR	95.2	Unclear
Pfundmair et al. (2022)	Jihadi	J	C	NR	P	LT	OS	E	86	21.33	97.7	Unclear
Pitcavage (2015)	Lone‐actor	T	C	1993–2012	P	LT	OS: ADL	US	35	NR	100	Unclear
Prendergast (2020)	Right‐Wing	T	C	1948–2017	P	LT	OS: PRIUS	US	922	37.56	94.4	Predominantly ‘White: 95.37%’ (p. 36).
Simi et al. (2015)	Right‐Wing	R	C	NR	D	LT	Int	US	44	NR	NR	Unclear
Simi et al. (2016)	Right‐Wing	J	C	NR	D	LT	Int	US	44	NR	86.4	Unclear
Thijssen et al. (2021)	Mixed	J		2014–2020	D	LT	CS	Netherlands	82	NR	91.5	Unclear
van Leyenhorst and Andreas (2017)	Jihadi	J		2015	D	LT	CS	Netherlands	26	21.00	75	Unclear
Weenink (2019)	Jihadi	J		2012–2016	P	LT	CS	Netherlands	319	24.00	69	Unclear
Weenink (2015)	Jihadi	J		2012–2014	D	LT	CS	Netherlands	140	24.00	84	Unclear
Zeman et al. (2018)	Lone‐actor	J		1998–2016	D	LT	OS	US, Ca, E, Switzerland, Norway, Australia	93	NR	97	Unclear

Abbreviations: Au, Australia; C (under prevalence), current; Ca, Canada; C (under design), cohort, CC, case control; CS, cross sectional; D, diagnosed disorders; E, Europe; J, journal article; LT, lifetime; NI, Northern Ireland; NZ, New Zealand; P, psychological problems; R, report, book; S, suspected disorders; T, thesis.

^a^ Further information sought from authors.

^b^ Author provided data for *n* = 47. *N* = 45 in published paper.

^c^ Age/gender reported are for all samples combined.

^d^ Data for *n* = 526 provided by corresponding author.

**Table 7:** Measurement of terrorism in included studies

Study	Measurement of terrorist behaviour/Terrorist sample inclusion
Bakker (2006)	All terror incidents in Europe were reviewed between September 2001 to October 2006 (350 incidents). Authors selected incidents involving terrorists who claimed to be Jihadi fighters. Authors proceeded to select ‘those cases in which people had been formally charged and taken into custody for an extended period of time, in which they were convicted for terrorist activities or in which they committed suicide during the attack (the first London bombings) or after the attack (the Madrid bombings)’ (p. 17).
Bergen et al. (2017)	Unclear
Böckler et al. (2018)	The sample identified ‘all incidents of severe targeted violence in Germany between 1999 and 2013’ (p. 8). The terrorist sample ‘severe targeted school violence’ encompassed individuals whom ‘attempted or completed attacks in schools, were committed by a current of former student of the school, planned and executed with potentially lethal weapons and the intention to kill one or more persons associated with the school, where the attack at least commenced, and when the perpetrator had not yet reached the age of twenty‐five’ (p. 9). The terrorist sample ‘targeted attacks with ideological or religious background’ included those who ‘attempted or completed attacks against the political order in which the perpetrators referred to an ideological or religious worldview’ (p. 9).
Bronsard et al. (2022)	The sample included adolescents who had been prosecuted for ‘criminal association to commit terrorism’, and those who were a ‘minority at the moment we started our study in January 2018’ (p. 2). The sample were all noted to have ‘joined an ideology that advocates the use of violence in the name of Islam’ (p. 7).
Brym and Araj (2012)	The sample was drawn from ‘a 25 percent random sample of suicide bombers (*N* = 42) from a database of collective violence events that occurred in Israel, the West Bank, and Gaza between 2000 and 2005’ (p. 434).
Bubolz and Simi (2019)	‘The level of group involvement for members of our sample included 7 individuals who founded a White supremacist group and 37 subjects who were either core or peripheral members’ (p. 7).
Candilis et al. (2021)	The terrorist sample comprised of ‘incarcerated offenders convicted of terrorism under the Iraqi Anti‐Terrorism Law and held at a maximum‐security Baghdad prison’ (p. 4).
Capellan (2015)	Ideological perpetrators were categorised as those who ‘hold extremist values and beliefs, but the event itself does not have to be ideologically motivated’ (p. 400). Authors gathered their sample from the active shooter data set which ‘was compiled from government reports, previous scholarship, and media reports of events that occurred between 1970 and June 2014. The primary source, however, was the Kelly's active shooter report. The report identified 324 events that occurred between 1960 and 2012, including foiled attempts’ (p. 400).
Capellan and Anisin (2018)	The authors ‘employed an open‐source data collection strategy to identify and collect information on both failed and successful mass public shootings that occurred in the United States from 1984 to 2015’ (p. 241). The authors focused on ‘mass public shootings committed by offenders who adhered to either far‐right radicalism (racial, religious, among others) or Islamic or Jihad‐inspired radicalism, and Black nationalist ideologies’ (p. 242).
Capellan et al. (2019)	Authors used an ‘open‐source data collection strategy to identify and collect information on both failed and successful mass public shootings that occurred in the United States from 1966 to 2017’ (p. 815).
Chermak and Gruenewald (2015)	The sample comprised of ‘three types of domestic extremists: far‐Right homicide offenders, Left‐wing offenders (i.e., environmental and animal rights extremists), and members of Al Qaeda and affiliated movements (AQAM extremists) Domestic extremist perpetrators were identified from the Extremist Crime Database (ECDB)’ (p. 140). The sample of far‐Right extremists were included where they had ‘been arrested in relation to cases of homicide’, including attacks both ideologically and non‐ideologically motivated (p. 140). The sample of left‐wing offenders included ‘animal and environmental rights extremists who have been arrested and charged in relation to ideologically motivated violent crimes’ (p. 141). Lastly, members of Al Qaeda and affiliated movements (AQAMs) were included wherein they had been ‘arrested and charged in the United States for committing crimes of homicide, attempted homicide, and other crimes related to failed and foiled violent terrorist plots’ (p. 141).
Cherney et al. (2022)	Terrorist behaviour was measured as those who engage in ‘terrorism‐related activities’ or those ‘committing ideologically motivated illegal violent or non‐violent acts’ (p. 102), as well as individuals who joined a terrorist organisation or were otherwise associated with terrorist organisations.
Clemmow et al. (2020)	‘The defining criterion for assigning the label “lone‐actor terrorist” to an individual was whether subjects carried out or planned to carry out, alone, an attack in service of some form of ideology, for which they were convicted or died in the attempt. The lone‐actor terrorists in our sample can operate with or without command‐and‐control links’ (p. 457). Moreover, ‘all individuals planned their attack in the United States, Europe, or Australia between 1990 and the end of 2015’ (p. 457).
Corner and Gill (2015)	The sample included those who were ‘either convicted or died in the commission of their offense in the United States and Europe since 1990. The sample includes violent and nonviolent behaviours carried out by individuals and isolated dyads who either self‐radicalized or radicalized via a larger organization and then carried out acts external to command and control links’ (p. 26).
Corner et al. (2019)	Terrorist behaviour was measured as ‘individuals who engaged in or planned to engage in, lone‐actor terrorist attacks within the United States and Europe and were convicted for their actions or died in the commission of their offence’ (p. 114).
Corner and Gill (2020)	The sample was taken from ‘terrorist autobiographies to solicit relevant information regarding the terrorist life course’ (p. 505). Shapiro's (2013) bibliography was the initial source for identifying potential autobiographies, with autobiographies chosen based ‘on language (English and translated documents), timeframe (due to text availability, individuals active before 1900 were removed), admission of action, and availability’ (p. 505).
Corner and Gill (2021)	The sample was taken from ‘terrorist autobiographies to solicit relevant information regarding the terrorist life course’ (p. 699). Shapiro's (2013) bibliography was the initial source for identifying potential autobiographies, with autobiographies chosen based ‘on language (English and translated documents), timeframe (due to text availability, individuals active prior to 1900 were removed), admission of action, and availability’ (p. 699).
de Roy van Zuijdewijn and Bakker (2016)	The research ‘involved the construction of a database on perpetrators of lone‐actor terrorism within the European Union for the years between 2000 and 2014. Data were collected from open sources (court proceedings, media reports)’ (p. 42). Results identified ‘120 perpetrators of lone‐actor terrorism, involved in 98 plots and 72 actual attacks’ (p. 42).
Dhumad et al. (2020)	The sample consisted of ‘individuals convicted of terrorism under the Iraqi Anti‐terrorism Law’ (p. 75). Offences amongst the included sample included ‘killing civilians, affiliation with terrorist groups, planting bomb, and killing government officials or Iraqi war fighters, and other offences’ (p. 79).
Duits et al. (2022)	The sample was defined as ‘Jihadi terrorists convicted of terrorism in the Netherlands, Belgium, six German federal states, Austria and Sweden’ (p. 4).
FBI (2019)	‘While offenders may have affiliated or associated with a terrorist organization/ideological movement or may have received assistance from others at some stage during the planning or implementation of their attacks, they must have been both the primary architect and the primary actor in the attack action. The attack must have occurred within the US and the offender must have radicalized, at least primarily, within the United States’ (p. 10).
Freilich et al. (2019)	Authors included those who subscribed to a far‐right or Al Qaeda (or associated movements; AQAM) belief system who committed a violent act in the US since 1990 –‘For all included cases, the terrorist mush have been convicted of the crime, or was unable to be tried because they died before court proceedings began’ (p. 948).
Gibson (2018)	The sample consisted of ‘individuals who were converts to Islam and took part in radical activities’ and were ‘pulled from different studies on individuals who had committed some form of Islamic radical activity’ (p. 7). Moreover, individuals were included only if the ‘subject was a U.S. citizen or legal resident at the time of conversion’, ‘he subject's radical activities took place after September 11, 2001, and the radical activities were carried out, or attempted to be carried out, in the name of Islam’ (p. 9). Radical activities were defined as ‘a terrorist attack, took substantial steps to carry out an attack but was unsuccessful, travelled overseas to provide support for an Islamic foreign terrorist organization as defined by the United States Department of State, provided or attempted to provide support for an Islamic foreign terrorist organization as defined by the United States Department of State without traveling outside of the United States’ (p. 9).
Gill et al. (2014)	The sample ‘includes 119 individuals who engaged in or planned to engage in lone‐actor terrorism within the United States and Europe and were convicted for their actions or died in the commissioning of their offense’ (p. 425).
Gill (2015)	Terrorism was measured as ‘individuals who engaged in or planned to engage in, lone‐actor terrorist attacks within the United States and Europe and were convicted for their actions or died in the commission of their offence’ (p. 114).
Gill et al. (2021)	The terrorist sample included ‘those who perpetrated (or planned) their violence to occur in the United States’ (p. 1798). The sample is limited to post‐1990 events recorded via open‐source data.
Gill et al. (2022)	The sample includes individuals charged and convicted for terrorism in UK and where their behaviour involved ‘active plotting or commission of a violent terrorist act’ (p. 115).
Gottschalk and Gottschalk (2004)	The sample consisted of ‘90 Palestinian and Israeli Jewish terrorists currently incarcerated in Israeli jails or living in freedom’ (p. 41). This was achieved by the author first securing ‘authorization from various jailed terrorist leaders. In each prison, Palestinian terrorists of various groups agreed to meet only after having obtained the leader's collaboration and participation in the research’ (p. 41). Accessing terrorists living in freedom were identified by ‘Palestinian informants introduced by the Belgian consul in Jerusalem’ (p. 42).
Gruenewald et al. (2013)	The sample encompassed individuals who carried out ‘fatal attacks committed by far‐right extremists between 1990 and 2010’ in the United States. Moreover, for a ‘homicide to be included in this study, there must have been a response by law enforcement to the homicide (i.e., an arrest was made) and one or more extremist offender had to be charged in the respective case’. Lastly, ‘at least one perpetrator must have subscribed to a far‐right belief system and committed the homicide to further that belief system’ (p. 75).
Hamm et al. (2017)	The paper draws its ‘sample from all known cases of lone wolf terrorism in the US between 1940 and mid‐2016.’ The American Lone Wolf Terrorism Database was created by the authors (p. 23) and was built from ‘previous research, biographies, and memoirs, media sources, government reports, and most importantly, court documents, including criminal complaints, trial transcripts, supporting affidavits and letters, and medical and psychiatric evaluations’ (p. 24).
Haugstvedt and Koehler (2021)	Authors used ‘data from the PIRUS dataset published in November 2019’ (p. 6). Individuals were included ‘if they were arrested, indicted, and/or convicted of either engaging or planning to engage in ideologically motivated unlawful behaviour, or anyone who belonged to a designated terrorist organization or a violent extremist group’ (p. 6).
Horgan (2016)	The sample was limited to those offenders based in the United States. Open‐source information was used.
Khazaeli and Khoshnood (2019)	‘The focus of this present study is on lone‐actor terrorism from the beginning of 2015 until the end of 2016. In collecting data, we searched the GTD's dataset for terrorist attacks categorized as LAT (lone‐actor terrorist) attacks in 23 Western European countries’ (p. 31).
Jensen and Kane (2021)	The sample was drawn from ‘data on QAnon offenders in the United States compiled as a companion to the PIRUS database’ (p. 5). ‘To be included in the QAnon companion to PIRUS, an individual must have (1) radicalized, in whole or in part, in the United States; (2) espoused ideological beliefs that are related to the QAnon and/or Pizza gate conspiracy theories; and (3) committed a criminal act that resulted in their arrest, indictment, or death. Moreover, there must be evidence in reliable sources to support the conclusion that the individual's criminal act was related to their participation in the QAnon movement’ (p. 5). Meanwhile, ‘offenders who posted in support of QAnon but committed crimes with no apparent connection to the conspiracy theory, such as drug use violations, were excluded from the database’ (p. 5).
Kupper and Meloy (2021)	‘For this study, subjects were selected based upon the following criteria: 1. The attack was planned with a specific target in mind and designed to be witnessed by the public. 2. The offender acted alone with no affiliation to a terrorism group. 3. The incident was motivated primarily by personal grievances and/or violence‐justifying ideologies, excluding gang, organized, domestic, or state‐sponsored crime incidents. The number of casualties was not taken into consideration during the selection process, as some attacks did not result in any target injuries or deaths’ (p. 3).
LaFree et al. (2018)	‘The individuals in the database were included for committing ideologically motivated illegal violent or nonviolent acts, joining a designated terrorist organization, or associating with organizations whose leaders have been indicted of ideologically motivated violent offenses’ (p. 244).
LaMontagne (2019)	The entire PIRUS database (1865 subjects) was used in the current study. ‘To be included in the PIRUS dataset, one of the five criteria had to have been met: *The individual was arrested for committing an ideologically motivated crime, * The individual was indicted for committing an ideologically motivated crime, *The individual was killed as a result of their committing an ideologically motivated action, *The individual was determined to have been a member of a Designated Terrorist Organization (DTO) even if the group itself did not acknowledge the membership, * The individual was connected with an extremist organization whose head was indicted for an ideologically motivated violent offense. In addition to one of the criteria above, each individual must have:*Been radicalized within the United States,*Espoused or currently espouse ideological motives, and *There must be evidence their behaviours are linked to the ideological motives they espoused’ (p. 49).

Lankford (2013)	‘This study was designed to analyse terrorism, rampage, workplace, and school attacks that involved suicide attempts and occurred in the United States between 1990 and 2010’ (p. 257). Furthermore, ‘attacks were excluded if they involved fewer than two victims or were primarily domestic in nature’ (p. 258).
Liem et al. (2018)	Included lone‐actor terrorists were defined as ‘a single perpetrator (acting alone, in a dyad or triad), who committed an attack, and hence exclude those only threatening to commit an attack’ (p. 52). Furthermore, ‘cases were selected for inclusion if they occurred between January 1, 2000, and December31, 2014’ with the following inclusion criteria applying: ‘a) Violence, or the threat of violence, must be planned or carried out; (b) the perpetrator(s) must be an individual, dyad, or triad; (c) the perpetrator must act without any direct support in the planning, preparation, and execution of the attack; (d) the perpetrator's decision to act must not be directed by any group or other individuals; (e) the motivation cannot be purely personal material gain; and (f) the target of the attack extends beyond those victims who are immediately affected by the act’ (p. 52).
Lucas (2021)	The sample was ‘composed of individuals or groups who: are fiercely nationalistic (as opposed to universal and international in orientation); are anti‐global; suspicious of centralized federal authority; are reverent of individual liberty (especially their right to own guns, be free of taxes); believe in conspiracy theories that involve a grave threat to national sovereignty and/or personal liberty; believe that one's personal and/or national “way of life” is under attack and is either already lost or that the threat is imminent (sometimes such beliefs are amorphous and vague, but for some the threat is from a specific ethnic, racial, or religious group); and believe in the need to be prepared for an attack either by participating in or supporting the need for paramilitary preparations and training or survivalism’ (p. 19/20).
Lyons and Harbison (1986)	‘The period of study is from 1974‐84. All of those included had been charged with murder’ (p. 194). Moreover, ‘only those directly involved in a violent act, e.g., pulling the trigger or planting the bomb, were included’ (p. 194).
Merari and Ganor (2020)	‘The participants in this study were Palestinian prisoners who had been arrested for carrying out attacks against Israeli civilians or security forces’ (p. 3). Moreover, ‘a criterion for selection was that there had been no involvement of a terrorist group in the decision planning, preparation and execution of the attacks committed by the participants. Another criterion for selection was the type of attack. The selected participants had carried out attacks that involved a risk of being killed, wounded and/or captured. The weapons used for the attacks were knives, firearms, or cars. Stone and Molotov cocktail throwers were excluded from the sample’ (p. 4).
Merari et al. (2010)	The sample included ‘“three groups of jailed Palestinian terrorists: (a) would‐be suicide bombers, (b) a control group of terrorists arrested for participation in non‐suicide missions, and (c) organizers of suicide operations. The suicides' sample included fifteen men, who had been arrested in the process of trying to carry out a suicide attack’ (p. 89). ‘The control sample included 12 men, who had been tried and jailed for participation in various political violence activities, ranging from stone throwing to armed assaults’ and the ‘organizers sample included fourteen who had been jailed for commanding and coordinating suicide attacks’ (p. 90).

Merari (2021)	‘The current paper synthesises the data gathered from Merari and Ganor (2020) and Merari et al. (2010). As such, the participants in this study were Palestinian prisoners who had been arrested for carrying out attacks against Israeli civilians or security forces’.
Meloy et al. (2019)	The sample consisted of ‘North American subjects who engaged in a lethal terrorist attack’ (p. 95). For the 33 lone‐actor terrorists included in the study, they must have ‘committed a politically motivated lethal or near lethal attack against non‐combatants in North America between 1993 and 2016’ (p. 96).
Perry et al. (2018)	The sample was derived from a ‘population of vehicle‐borne terrorist attacks committed by lone‐actor predators in Israel and the West Bank between January 2000 and March 2016’ (p. 904). In this study, lone‐actors were defined as ‘an unaffiliated individual who acts on his or her own without orders from—or even connections to—an organization’ may ‘collaborate with other individuals’, and ‘may be inspired by the actions or the ideology of an organization, without being in direct contact with any of its member’ (p. 901).
Pfundmair et al. (2022)	‘All subjects were radicals who had been reported in newspapers for various reasons: Some had planned or committed a terrorist attack, others had returned from Syria and were charged in court, others had given an interview to declare they turned away from radical Islam’ (p. 58).
Pitcavage (2015)	The date range ‘1993 to 2012’ was applied to the sample in this study. ‘The perpetrators selected for this comparison all killed at least one person’ thus including only ‘those individuals who actually were successful in committing a lethal act of lone wolf violence’ (p. 1666).
Pendergast (2020)	The current study ‘examines violent and non‐violent outcomes among a sample of extremists publicly identified for their ideological behaviour and affiliations’ (p. 37) as captured in the PIRUS data set. Terrorists with multiple ideologies were defined as when ‘extremists are associated with more than one extremist ideology, either concurrently or moving from one ideology to another. Single ideology peers are only associated with one ideology’ (p. 2).
Simi et al. (2015)	The sample consisted of ‘former members of violent white supremacist groups who lived in 15 different states across all regions of the United States’, with ‘initial contacts with former white supremacists based on the long‐term ethnographic fieldwork of the lead researcher’ (p. 2).
Simi et al. (2016)	The sample consisted of ‘former members of violent white supremacist groups who lived in 15 different states across all regions of the United States’, with ‘initial contacts with former white supremacists based on the long‐term ethnographic fieldwork of the lead researcher’ (p. 541). ‘The study also relied on contacting former extremists with a public presence who have either written books about their lives or shared their experiences in some type of public forum. Each of the initial subjects was asked to provide referrals to other former extremists who might be willing to participate in the study’ (p. 541).
Thijssen et al. (2021)	The sample consisted of individuals who had been convicted of terrorism, being detained in the ‘terrorism wings of the penitentiary in Vught’ between 2014 and 2020 (p. 6).
van Leyenhorst and Andreas (2017)	The sample consisted of those ‘who, in the Netherlands, are suspected of, or prosecuted for, terrorism‐related offenses connected to Salafi‐Jihadi terrorism in and around Syria. Our sample was restricted to prosecutions where the DPS was tasked to perform advisory, supervisory and/or reintegration work’ (p. 313). However, ‘all of the files were compiled in the pre‐trial period, so all of the individuals in our sample were suspects at the time their files were selected (i.e., 2015)’ (p. 313).
Weenink (2019)	The sample consisted of ‘Jihadi travellers from the Netherlands’ which was comprised of ‘all individuals on the “List of jihadi travellers”, which was compiled by the counterterrorism team in the Central Unit of the Netherlands Police’ (p. 132). ‘In Study 1, the author used the list from February 2014 (List 1), which contained the personal details of all 140 travellers known to Dutch police at that moment. Study 2 uses the list from March2016 (List 2) (*n* = 319); 108 individuals from List 1 are also on List 2’ (p. 132).

Weenink (2015)	‘The sample is a list containing personal details of radical Islamists from the Netherlands whom the Dutch police suspect of having joined the fight in Syria, or are considered potential travellers (for example, because they have expressed their intent to do so). The list is a national “List of Travellers” (LOT), as compiled by the Counterterrorism and Extremism (CTE) team in the Central Unit of the Dutch National Police’ (p. 19).
Zeman et al. (2018)	The sample ‘contains the cases of lone wolves’ committing their terrorist attacks in the United States, Canada, the European Union, Switzerland, Norway and Australia from 1998 to 2016’ (p. 9). The Global Terrorist Database (GTD) was the source from which cases were selected. Only ‘Unaffiliated Individuals’ in the GTD were selected for inclusion, defined as ‘an individual who is not affiliated to a perpetrator group’ (p. 9).

###### Year, publication type and jurisdiction

Papers were published from 1986 (e.g., Lyons & Harbinson, 1986) to 2022 (e.g., Gill et al., 2022). The majority of the included papers (*k* = 48) were published since 2015 (86% of papers). Most of the papers (*k* = 43 papers; 77%) were published in peer‐reviewed journals. With the exception of two studies based on the same data set of terrorist offenders incarcerated in Iraq (Candilis et al., 2021; Dhumad et al., 2020), and five of offenders active in Israel and Palestine (Brym & Araj, 2012; Gottschalk & Gottschalk, 2004; Merari & Ganor, 2020; Merari et al., 2010; Perry et al., 2018), all other studies drew on samples of terrorists who operated in the West.

###### Sample size and participant profile

Sample sizes ranged from *n* = 11 to *n* = 1715. At the lower end, 12 studies reported prevalence rates based on samples of less than 20 participants. The average sample size across all 68 studies was 181. Included in this average are studies that reported samples sizes for sub‐samples and the sample as a whole (e.g., Chermak and Gruenewald (2015) report sample sizes for their sub‐samples (far right; far‐left; Al‐Qaeda affiliate) and for the full sample).

The mean age across samples reporting mean age (as opposed to range, median or mode only) was 27.8, with a range of 17 (Cherney et al., 2022) to 42 (Jensen & Kane, 2021). Two studies presented data relevant to developmental age. As anticipated, for all studies where gender composition was reported, the sample was comprised either primarily or entirely of males. The majority of studies (80.1%) had samples where males made up 80% or more of the sample. The average proportion of males across all samples was 90.4%.

###### Data sources and settings

The majority of studies used open‐source databases of terrorist offenders that were either pre‐existing or were constructed for individual studies (48 studies). Open‐source databases typically relied on media coverage, court documents, government reports on individual terrorists and from which critical information relevant to mental health was imputed into the datasets. This includes Cherney et al's. (2022) investigation of the processes leading to radicalisation into terrorism in Australia. They developed a database based on open sources ‘(e.g., court documents and media reports) to compile variables on individuals who have radicalised in Australia across different ideological spectrums’ (p. 98). The codebook for that data set was informed by the Profiles of Individual Radicalisation in the United States (PIRUS) data set, and which was used in other studies included in the review (Haugstvedt & Koehler, 2021; Jensen & Kane, 2021; LaFree et al., 2018; LaMontagne, 2019; Pendergast, 2020). Another open‐source data set, the United States Extremist Crime Database (ECDB), was also used in multiple studies (Chermack & Freilich et al., 2019; Gruenewald et al., 2013; Gruenewald, 2015).

Seven papers (8 studies) reported data based on closed source datasets (data compiled from restricted files held by the statutory services including health, security and law‐enforcement) or datasets based on a combination of closed and open sources (e.g., Duits et al., 2022; FBI, 2019; Gill et al., 2022; Perry et al., 2018; Thijssen et al., 2021; Weenink, 2015; Weenink, 2019). Some studies were based on interviews with terrorists or former terrorists in community settings (e.g., Bubolz & Simi, 2019; Simi et al., 2015; Simi et al., 2016), while others involved clinical interviews with terrorist offenders recruited through the criminal justice system (e.g., Bonsard et al., 2022; Candilis et al., 2021; Dhumad et al., 2020; Lyons & Harbinson, 1986; Merari & Ganor, 2020).

###### Outcomes—Forms of terrorism

Papers focused on different forms of terrorism (see Table 7). We coded these as ‘Lone Actor terrorism’, ‘Right‐Wing terrorism’, ‘Jihadi terrorism’, ‘mixed’ and ‘other’, reflecting the key forms of terrorism examined in the included studies. Some papers focused on lone‐actors who were not‐affiliated with others and acted alone (e.g., Gruenewald et al., 2013; Pitcavage, 2015). Others, however, adopted broad conceptualisations of lone‐actor terrorism. This includes Liem et al. (2018) who sampled individuals who operated in a ‘small cell’ in their definition of lone actor terrorism (‘the use of violence by a single perpetrator (or small cell)… who acts without any direct support in the planning, preparation and execution of the attack, and whose decision to act is not directed’, p. 51). A similar definition was used by Khazaeli Jah and Khoshnood (2019) who included those who were acted alone or as part of a ‘small cell… without direct support’ (p. 26). The FBI's 2019 report of lone actor terrorism only required that lone actors were ‘the primary architect and primary actor’', with some in the sample receiving ‘assistance from others at some stage during the planning or implementation of their attacks (p. 10). Gill et al. (2022) used an ‘expansive interpretation of what constitutes lone‐actor terrorism’ and included those who ‘operate autonomously of a group’, ‘solo terrorists… trained and equipped by a group—which may also choose their targets—but attempt to carry out their attacks autonomously’ and ‘isolated dyads… who operate independently of a group’ (p. 115). The same interpretation of lone‐actor was used in an earlier paper (Gill et al., 2014).

Eleven studies (i.e., samples) focused reported specifically on right wing (far‐right) terrorists. With the exception of Lucas' (2021) sample of right wing terrorists in Germany, all were based on terrorists active in the US (Bubolz & Simi, 2019; Chermak & Gruenewald, 2015; Pendergast, 2020). This excludes one study that reported specifically on QAnon terrorists, and categorised as ‘other’ in this review (Jensen & Kane, 2021).

Thirteen studies reported on a sample of terrorists described as Jihadi, Al‐Qaeda or similarly affiliated groups. This includes three papers focusing on Jihadi from the Netherlands—two papers by Weenink (2015 & 2019) who reported on individuals who left, or attempted to leave the Netherlands, for Syria and a third paper that reported on ‘Dutch Jihadists’ suspected or convicted of terrorist offences (van Leyenhorst & Andreas, 2017). Duits et al. (2022) included a sample of Jihadi terrorists who had been convicted of terrorism between 2012 and 2021 in Europe and Pfundmair et al. (2022) reported on 86 Jihadi ‘radicals’ in Western Europe who had ‘committed a terrorist attack… returned from Syria and were charged in court… [or] had given an interview to declare they had turned away from radical Islam’ (p. 58). Chermack and Gruenewald's (2015) also reported on a sample of Al‐Qaeda affiliated terrorists, this time based on terrorists active in the US, as did Bergen et al. (2017) (which was excluded from the data synthesis as we are awaiting additional information from the authors as to the number of positive cases of suspected disorder from the sample of 415 individuals).

Some studies included samples of terrorists without differentiating, in the presentation of data pertaining to mental health, between specific forms of terrorism, and categorised here as ‘mixed’ samples. This includes Corner and Gill's (2020) study of a sample including ethno‐nationalist, right‐wing, religious, and single‐issue terrorism, Freilich et al. (2019) sample of far‐right and Al‐Qaeda (and associated) terrorists, Jensen and Kane's (2021) sample of far‐right, far‐left and single issues terrorists from the PIRUS database and Cherney et al.'s (2022) sample of white supremacists, single issue terrorists and ‘Islamists’ (p. 103).

###### Predictor/Risk—Mental health difficulties

All studies reported on mental health difficulties in terrorist samples, though some reported on diagnosed disorders (*k* = 36), some on what we refer to as ‘suspected disorders’ (*k* = 17) and others psychological problems (*k* = 35). Some reported on more than one category of difficulty (e.g., diagnosed disorders and psychological problems).

A number of papers were based on clinical interviews by trained professionals reported rates of diagnosed mental disorder. For example, in an early study of group‐based ‘para‐military’ terrorism in Northern Ireland, Lyons and Harbinson (1986), both mental health professionals, reported on their psychiatric assessments of 47 offenders. Their assessment included clinical interview, review of case notes and the gathering of collateral information from ‘next‐of‐kin’ (p. 194). Dhumad et al. (2020) interviewed 160 terrorists incarcerated in a prison in Bagdad, Iraq, and assessed for features of childhood conduct disorder and anti‐social personality disorder (ASPD) based on DSM‐V criteria. Interviews were conducted ‘by researchers who received specific training in administering the study tool’ (p. 76). Similarly, Merari and Ganor (2020) conducted clinical interviews with 39 incarcerated lone‐actor Palestinian terrorists. The interviews were completed by clinical psychologists and included administration of the Thematic Apperception Test (TAT), the Minnesota Multi‐phasic Personality Inventory 2 (MMPI‐2) and Structured Clinical interview for DSM 5 screening personality questionnaire (SCID‐5 SPQ). Where indicated, the full SCID 5 for assessment of personality disorder was also administered. Merari and Ganor (2020) and Lyons and Harbison's (1986) studies reported on the extent to which the sample met the diagnostic criteria for mental disorders at the time of interview (i.e., the point prevalence was ‘now’), whereas Dhumad et al.'s (2020) study was of point prevalence (‘now’) of anti‐social personality disorder and period prevalence (childhood) of conduct disorder.

Some studies were based on bespoke or pre‐existing datasets. Liem et al.'s (2018) sample of EU‐based lone actor terrorists was based on open‐source data. They coded disorder as present where there was ‘some or sure indications of mental illness’ (p. 54), but later provided sufficient information to isolate a sub‐sample of individuals who ‘underwent a clinical examination and were diagnosed with a particular mental disorder’ (p. 60).

However, for some studies based on open‐source datasets it was not always clear what level of evidence was required for an individual in the data set to be classified as having a mental disorder. As noted earlier, where authors described their samples as having mental disorders, but the information provided did not clearly suggest this was the case, we classified these studies as reporting on ‘suspected disorders’. For example, in Corner and Gill's (2020) data set of 91 terrorists, and based on autobiographical data, they report that ‘due to the sensitive nature of specific subjects, such as abuse and mental disorder, there was an inherent lack of disclosure. In these cases, it was necessary to deduce possible occurrences from available information’ (p. 506). They used diagnostic information in the autobiographies to make inferences in relation to the presence or absence of suspected mental disorder. Other studies were similarly classified as reporting on suspected mental disorder, including Gruenewald, Chermack and Freilich (2013) studies of right‐wing terrorists, based on the ECDB open‐source database and where they had a ‘reported history of mental illness’ (p. 77) but there was no clear reference to ‘diagnosed’ or ‘confirmed’ disorder.

Freilich et al. (2019) also used the ECDB data set, this time to explore a mixed sample of right‐wing and Al‐Qaeda (or associated) terrorists. However, they enriched the data set with additional data to allow them to report ‘on a history of diagnosed mental illness that was recorded in the open‐source documents’ (p. 950). Consequently, Freilich et al.'s study was classified in the review as reporting on diagnosed disorder (rates for psychological problems are reported separately).

Other studies reported more broadly on psychological problems, including Weeninks (2019) study of Dutch jihadis based on closed‐sources and Capellan and Anisin (2018) study of lone‐actors in the US. In the latter they report on the presence of ‘mental disturbance’ which includes diagnosed disorders and psychological problems (‘adverse psychological processes’) (p. 244).

Some studies reported the point prevalence of difficulties at that time (i.e. a point‐prevalence based on ‘right now’) (6 papers, reporting on 10 studies/samples) and these are discussed further below (see *Point‐prevalence rates of mental health difficulties*. All other studies reported lifetime prevalence including, in some studies, across the full life‐trajectory of terrorist involvement from radicalisation to exiting from terrorism.

Finally, it is relevant to note that many of the studies considered mental health difficulties as just one of many potential characteristics of terrorist samples or potential processes implicated in violent radicalisation into terrorism. In part, this explains why there was variation in the extent to which authors attended to the reliability and validity of classifying difficulties as present or absent using the data at their disposal.

##### Point‐prevalence rates of mental health difficulties

Six papers with 10 samples/studies reported point‐prevalence rates of mental health difficulties (i.e., presence of diffiuclties at a point in time). However one of these, Merari (2021) is a book chapter that presents data already captured in the other studies (Merari & Ganor 2020; Merari et al., 2010). Focusing on the remaining 5 papers (7 studies), 4 involved clinical interviews with individuals involved with the criminal justice system for terrorist activities (Bronsard et al., 2022; Lyons & Harbinson, 1986; Merari et al., 2010; Merari & Ganor, 2020). Bakker's (2006) study of Jihadi terrorists in Europe was based on open source information.

Excluding Bakker's (2006) sample of 242 individuals, less than 50 individuals were recruited to the other studies (Bronsard et al., 2022 *n* = 15; Lyons & Harbinson, 1986 *n* = 47; Merari et al., 2010 *n* = 41 (all samples); Merari & Ganor, 2020 *n* = 39). Both papers from Merari focused on Palestinian terrorism and invovled interviews with incarcerated terrorists. Lyons and Harbinson (1986) report on their interviews with terrorist offenders from Northern Ireland referred for psychiatric assessment pre‐sentencing and ‘seen at the request of the defending solicitor or barrister’ (p. 194). Bronsard et al. (2022) interviewed 15 minors convicted for involvement in, or attempting to become invovled in, Jihadi terrorism in France.

All studies reported suspected diagnoses or confirmed diagnoses based on clinical interviews. Bronsard et al., (2022) used the MINI‐KID 2, a semi‐structured clinical interview sensitve to DSM and ICD diagnostic categories, the Abbreviated‐Diagnostic Interview for Borderline Personality Disorder (Ab‐DIB) and the WAIS‐IV (Ravens Matrices) for intelligence among other diagnostic and screening tools. Merari (2020, 2010) used projective tests (e.g., Rorschach Test), screening and diagnostic tests (e.g., Minnesota Multiphasic Personality Inventory 2; MMPI‐2), and structured clinical interview (e.g., SCID). Lyons and Harbinson (1986) used a structured interview in their assessment of mental disorder.

#### 5.2.2 Excluded studies

The majority of the full‐texts reviewed did not meet the review eligibility criteria for Objective 1 and references for these studies are included under ‘References to excluded studies’. A number of studies that might be expected to be included were also excluded as this stage and these are detailed below.

Two papers were excluded as it was not clear that their samples were comprised of those who had engaged in terrorist behaviour. Morris and Meloy (2020) report prevalence rates of mental disorder among 23 individuals referred to the Prevent programme in Scotland as being at risk of becoming involved in terrorism, and who were described at the time of their assessment as ‘lone‐actors‘ in that ‘none were embedded with organized terrorist groups’ (p. 1638). The paper was excluded as the sample was comprised of individuals ‘at risk of involvement' (p. 1638) rather than involved, and thus did not meet our inclusion criteria. Thirty‐nine percent of that sample had a history of a mental disorder diagnosed by a consultant psychiatrist, most commonly a substance use disorder (26%, *n* = 6).

King et al. (2018) was also excluded because we were not clear that the sample had engaged in terrorist behaviour. They sampled prisoners ‘who had been assigned an Islamism‐related security label’ either by the intelligence service or on observations made by prison staff, and which may occur ‘if materials distributed or symbols used by such groups were found’ (p. 131). The authors stress that the ‘security labels were thus *not* primarily assigned based on the offences the inmate had committed’ (our emphasis, p. 131). We excluded the study as it was not clear that the individuals coded as being involved in Islamist Terrorism were involved in a terrorist group, ever engaged in terrorism, or had progressed beyond the process of radicalisation. King and colleagues reported that 31% of the ‘Islamist Terrorism’ group had some indicators of mental health issues, though sample size was small (20 per group).

We excluded Meloy, Goodwill, Clemmow and Gill (2021) study based on an analysis of 125 lone actors, primarily from North America and Europe. The paper did not report prevalence rates of mental health difficulties and we did not seek this information from the authors as the data set is the same as that used in Corner et al. (2019), which is included in the review. Nor were we able to extract data from Clemmow et al. (2020). That paper draws on two pre‐existing datasets, both of which are already represented in the review (Corner et al., 2019; Horgan, 2016). We excluded Clemmow, Schumann et al. (2020) for the same reason (i.e., the study used the same data set as Corner et al., 2019 and Horgan, 2016).

Finally, we excluded Spaaij (2010) study of US lone‐actors. The study adopted a mixed methods approach comprised of a quantitative analysis of 74 individuals and detailed case studies of 5 individuals. Mental health difficulties were only considered in the case study research and thus the study did not meet our eligibility criteria and was excluded. Of the 5 individual case studies, 3 of the lone‐actors had a diagnosed personality disorder and one had been diagnosed with obsessive‐compulsive disorder.

#### 5.2.3 Risk of bias in included studies for Objective 1

We used the *JBI Checklist for Prevalence Studies* (JBI, 2020), for all papers that were eligible for consideration for our synthesis of prevalence rates of disorder among terrorist samples. For each paper, specific reasons for risk ratings for each item are reported. We use the Risk of Bias (ROB) assessment to draw attention to the methodological limitations and other sources of bias in this body of literature in the review rather than use it to isolate a set of high quality papers for inclusion in the meta‐analysis and narrative synthesis.

Our rationale for this is that research involving terrorist samples is inherently challenging, particularly in relation to data quality—accessing terrorist samples for the purpose of building profiles is very difficult, often necessitating that data sets are based on the compilation of data from a range of open‐sources. If we were to exclude studies where samples were not demonstrably representative of the wider terrorist population, or where mental disorders were not assessed through clinical interview or valid psychometric assessment, the consequence would be a limited review. We would also note, again, that while the presence of mental health difficulties was reported in all studies, it was not a specific objective, or primary focus, of some studies and rather one of many characteristics of terrorists reported on—therefore, for some studies our ROB is focused on an aspect of these studies that was not necessarily a primary focus. We also acknowledge that our ROB is based on information published in each study, and which may, in part, reflect the conventions of the relevant journal rather than the ROB of the study itself.

Table 8 summarises our risk of bias assessment for all papers across the 9 items of the JBI checklist, as well as the tenth item on plausibility. The ROB was completed in duplicate with just one discrepancy across the two reviewers (*κ* = 0.98) and which was resolved through discussion. We have sought further information on 5 papers to allow us complete both the risk of bias assessment and extract data for the data synthesis. Excluding these from the 56 papers, left 51 for full risk of bias assessment. As anticipated, 98% of papers (50 of 51 papers) were assessed as having high risk of bias as each received a rating of ‘High’ for at least one item. Only one paper (Duits et al., 2022) had all items as rated ‘Low’, thus achieving the low risk of bias designation.

**Table 8:** Risk of bias assessment according to the JBI checklist for prevalence data

Study	1. Representative	2. Recruitment	3. Sample Size	4. Description of participants	5. Coverage	6. Standard criteria	7. Measurement	8. Statistics	9. Missing data analysis	10. Plausibility	Risk of bias rating
Bakker (2006)	Low	Low	Low	Low	High	Unclear	High	Low	High	High	High
Bergen et al. (2017)^a^	NA	NA	NA	NA	NA	NA	NA	NA	NA	NA	?
Böckler et al. (2018)^a^	NA	NA	NA	NA	NA	NA	NA	NA	NA	NA	?
Bronsard et al. (2022)	Low	High	Low	Low	Low	Low	Low	Low	Low	High	High
Brym and Araj (2012)^a^	NA	NA	NA	NA	NA	NA	NA	NA	NA	NA	?
Bubolz and Simi (2019)	High	High	High	Low	Low	Low	Low	High	Low	Low	High
Candilis et al. (2021)	Low	Low	Low	Low	Low	Low	Low	Low	Low	High	High
Capellan (2015)	Low	Low	High	Low	Low	Low	Low	Low	High	Low	High
Capellan and Anisin (2018)	Low	Low	Low	High	Low	NA	NA	Low	Low	Low	High
Capellan et al. (2019)	Low	Low	Low	Low	High	Low	Low	High	High	High	High
Chermak and Gruenewald (2015)	High	Low	Unclear	Low	High	Low	Unclear	High	High	High	High
Cherney et al. (2022)	Low	Low	Low	Low	Low	Low	Low	Low	Low	High	High
Clemmow et al. (2020)	Low	High	Low	Low	High	Low	Unclear	High	High	High	High
Corner and Gill (2015)	High	Low	Unclear	High	High	Low	High	High	High	Low	High
Corner et al. (2019)	High	Low	Unclear	High	High	Low	Unclear	High	High	Low	High
Corner and Gill (2020)	High	Low	Unclear	High	High	Low	Unclear	High	High	Low	High
Corner and Gill (2021)	High	Low	Unclear	Low	High	Low	Unclear	High	High	Low	High
de Roy van Zuijdewijn and Bakker (2016)	High	Low	Unclear	Low	High	High	High	High	High	Unclear	High
Dhumad et al. (2020)	High	Unclear	Unclear	Low	Low	Low	High	High	Low	Low	High
Duits et al. (2022)	Low	Low	Low	Low	Low	Low	Low	Low	Low	Low	Low
FBI (2019)	High	High	Unclear	Low	High	Low	Low	Low	High	High	High
Freilich et al. (2019)	Low	Low	Unclear	Low	Low	Low	Unclear	High	Low	High	High
Gibson (2018)	High	High	High	Low	High	High	High	Low	High	High	High
Gill et al. (2014)	Low	High	Unclear	Low	High	Low	Unclear	High	High	High	High
Gill (2015)	Low	Low	Unclear	Low	High	NA	NA	High	High	Low	High
Gill et al. (2021)	Low	Low	Unclear	Low	High	Low	Unclear	High	Low	High	High
Gill et al. (2022)	High	Low	Unclear	High	High	Low	Low	Low	High	High	High
Gottschalk and Gottschalk (2004)^1^	NA	NA	NA	NA	NA	NA	NA	NA	NA	NA	?
Gruenewald et al. (2013)	Low	Unclear	Unclear	Low	High	Unclear	Unclear	Low	High	High	High
Hamm, Spaaij, Hamm and Spaaij (2017)	Low	Low	Low	Low	Low	Low	High	Low	Low	High	High
Haugstvedt and Koehler (2021)	Low	Low	Low	High	High	Unclear	Unclear	High	High	Low	High
Horgan (2016)	Low	Low	Low	Low	High	NA	Unclear	Low	High	Low	High
Khazaeli and Khoshnood (2019)	Low	Low	Low	Low	High	NA	NA	High	High	High	High
Jensen and Kane (2021)	High	Low	Low	Low	Low	Low	Low	Low	High	High	High
Kupper and Meloy (2021)	High	High	High	High	High	Unclear	High	Low	High	High	High
LaFree et al. (2018)	Low	Low	Low	Low	Low	Low	Unclear	Low	High	Low	High
LaMontagne (2019)	Low	Low	Low	High	High	NA	NA	Low	Low	High	High
Lankford (2013)^a^	NA	NA	NA	NA	NA	NA	NA	NA	NA	NA	?
Liem et al. (2018)	Low	Low	Low	Low	Low	Low	Low	Low	High	High	High
Lucas (2021)	Low	Low	Low	Low	High	High	High	High	High	Low	High
Lyons and Harbison (1986)	High	High	High	Low	Low	Low	Low	Low	Low	High	High
Merari and Ganor (2020)	Low	High	High	Low	Low	Low	Low	Low	Low	Low	High
Merari et al. (2010)	Low	Low	Low	Low	Low	Low	Low	Low	Low	Low	High
Merari (2021)	Low	High	High	Low	Low	Low	Low	Low	Low	Low	High
Meloy et al. (2019)	High	High	High	Low	High	High	High	Low	High	High	High
Perry et al. (2018)	Low	Low	Low	Low	Low	NA	NA	Low	High	High	High
Pfundmair et al. (2022)	High	High	High	Low	High	NA	NA	Low	High	High	High
Pitcavage (2015)	High	Low	Low	High	High	NA	NA	Low	High	Low	High
Pendergast (2020)	High	Low	Low	Low	High	NA	NA	Low	High	High	High
Simi et al. (2015)	High	High	High	Low	Low	Low	Low	High	Low	Low	High
Simi et al. (2016)	High	High	Unclear	Low	Low	Low	Low	Low	Low	Low	High
Thijssen et al. (2021)	Low	Low	Low	Low	High	Low	Low	Low	High	High	High
van Leyenhorst & Andreas (2017)	High	Low	Low	Low	High	Low	Low	Low	High	High	High
Weenink (2019)	Low	Low	Low	Low	High	NA	NA	Low	High	High	High
Weenink (2015)	Low	Low	Low	Low	High	Low	Low	Low	High	High	High
Zeman et al. (2018)	High	High	High	Low	Low	Low	Low	High	High	Low	High

^a^ Authors contacted for more detail on study to enable completion of ROB assessment.

The first two items on the checklist pertain to the representativeness of the sample (Item 1) and the appropriateness of the recruitment of the sample (Item 2). In relation to representativeness, we assessed each paper to the extent that ‘the sample frame was appropriate to address the target population’, with guidance suggesting that the sample frame should represent the target population (JBI, 2020). Importantly, the guidance suggests that a ‘sample frame may be appropriate when it includes almost all the members of the target population’. Hence, where papers sought to understand a specific form of terrorism within a specific jurisdiction and all known cases were sampled, a ‘low‐risk’ rating was applied. Twenty‐nine papers were identified as having low‐risk based on this criterion, with 22 rated as high‐risk. For example, Bakker's (2006) population of interest was Jihadi terrorists in Europe. They sampled 242 terrorists active in Europe between 2001 and 2006 and the information provided in the paper that all those identified as meeting their inclusion criteria were included. Based on the assumption that the sample was therefore likely to be representative, a rating of low‐risk of bias was allocated to the Bakker paper for Item 1. For other papers, however, authors acknowledged that the sample may not be representative (e.g., van Leyenhorst & Andreas, 2017), the full sample frame was not sampled (or it was not clearly stated the full sample frame was sampled) (e.g. Chermak & Gruenwald, 2015) or there was insufficient information to assess representativeness and it was not discussed by authors (e.g. Bubolz & Simi, 2019). In all such cases a rating of ‘high‐risk’ was applied.

When assessed in terms of the appropriateness of the recruitment of their samples (Item 2), 34 papers were assessed as low risk, 15 as high‐risk and 3 as unclear. Based on guidance, sampling was assessed as low risk where random sampling was used (e.g., LaFree et al., 2018) or where everyone, or almost all individuals, in a sampling frame were recruited. This meant that even though some data sets may not have been representative of the global target population, once everyone within the data set was recruited we assessed this as low‐risk of bias.

Item 3 on the JBI checklist assesses the appropriateness of the sample sizes included. We acknowledge that sample size of individual studies is not a primary concern when they are included in a meta‐analyses, but retained the item here for completeness and to help in our assessment of each included study.

As studies included do not report sample size calculations, and following advice in the JBI checklist, we calculated a threshold using the following formula:

$$n=\left(\left(Z^{2}\right)\left(P\right)\left(1-P\right)\right)/d^{2},$$

where *n* is the sample size, *Z* the Z statistic for the desired confidence interval (in this case 0.95%, and the *Z* value of 1.96), *P* the expected prevalence of the proportion (the lifetime rate of disorder in the general population of 25% (p.25) is used), and *d* the precision (set as half the Confidence Interval, or 0.05). This yielded a target population size of 294. We accept that this sets a very high threshold for terrorism research, and apply the sample size calculation as a benchmark only. We coded papers that met or exceeded *n* = 294 as low‐risk and those lower than *n* = 294 as high‐risk. Papers where the full, or almost all, of the sample frame were recruited were also coded as low‐risk regardless of the sample size (as arises, e.g., in the van Leyenhorst and Andreas paper, 2017). Twenty‐five papers were coded as low‐risk and 11 as high‐risk. Fifteen papers were marked as unclear, primarily because there was a lack of information in the papers as to how they determined their target sample size.

Item 4 relates to descriptions of the sample and setting and here we coded that criterion as met where the study reported demographic characteristics of age (mean and/or range) and gender, and the form of terrorism at a minimum. Nine of the papers were scored as high‐risk having not reported either age or gender. All other studies were assessed as low‐risk.

Items 5 and 9 related to data‐analysis and response rates. We used both items to assess the risk of bias that can arise when missing values are not appropriately reported and managed. To be assessed as low‐risk, papers had to report the number of missing values for the mental health difficulties (Item 5). For Item 9, to be assessed as low‐risk they had to compare those for whom data is missing with those for whom data is present (Item 9). The rationale for this has been discussed in the literature, with the omission of missing cases from the denominator in calculating proportions leading to an inflation in prevalence rates (e.g., Freilich et al., 2019; Gill et al., 2022).

While almost all papers calculated prevalence rates with missing cases included in the denominator, they do not report what proportion of the denominator is based on ‘missing data’ vs mental health difficulties reported to be absent (coded as high‐risk for Item 5). In addition, some papers do not compare missing cases with non‐missing cases, and which would inform our understanding of data‐coverage bias (Item 9). Twenty‐one papers were assessed as low risk for Items 5 and 17 for Item 9. Papers based on interview or clinical assessment tended to score as low‐risk for these items as they explicitly probed both presence and absence of difficulties leading to no or low missing data (i.e., Bronsard et al., 2022; Bubolz & Simi, 2019; Candilis et al., 2021; Dhumad et al., 2020; Lyons & Harbinson, 1986; Merari & Ganor, 2020; Simi et al., 2015; Simi et al., 2016).

Item 6 assesses the validity of the measurement of mental disorder, and was coded as low‐risk where there was reference to ‘confirmed’ or ‘formal’ etc., diagnosis. Item 7 assessed the risk of bias that may arises when assessment of diagnosis is made by individuals who are not trained in diagnostic interviews and systems. This item was coded as low‐risk when individuals were trained. For both items, we coded studies as not‐applicable where they were not assessing mental disorder and were, instead, focused on psychological problems (10 papers for Item 6, and 9 papers for Item 7). However, they were coded high‐risk if they purported to be reporting on mental disorders but do not refer to how individuals were assessed (Item 6) or who conducted the assessment (Item 7). Thirty‐three papers were low‐risk for Item 6, and 21 papers were low risk for Item 7.

Item 8 assesses the appropriateness of the statistical analysis. As we are primarily interested in the positive events and total sample size for our meta‐analysis of prevalence rates, once both are reported, or sufficient data is present to allow their calculation (e.g., percentage and denominator), studies were assessed as low risk. Nineteen papers were assessed as high‐risk as a result of not including a sufficient amount of statistical information. However, 32 papers included the required information and were thus assessed as low‐risk. As an exemplar, the most comprehensive reporting of data relevant to this review was provided by Duits et al. (2022) who reported numerator, denominator and proportion/percentages. They compared two samples of extremists (younger vs older) and also presented odds ratio, 95% Confidence Intervals and results of inferential statistics.

Item 9 focused on missing data. A large number of ‘missing data’ or ‘unknowns’ can reduce the study's validity. As a result, to reduce risk of bias, authors should compare those in the study (found cases) with missing data/unknown cases to assess nonresponse biases. Thirty‐four papers were marked as high‐risk as a result of not considering the missing data in their study.

Finally, as part of our ROB, we also explored the extent to which studies presented a theoretical account of the link between mental health difficulties and terrorist behaviour (plausibility). Again, it is relevant to note that mental health was not the primary focus of many of the studies included. This said, 41% of the papers (*k* = 21) made some attempt to explain how difficulties may be linked to terrorism and we return to these explanations in the Discussion section of the review.

#### 5.2.4 Synthesis of results for Objective 1

As noted earlier, we have classified the included studies as reporting on diagnosed mental disorder, suspected disorder, and psychological problems, with some studies reporting on more than one of these categories. In our synthesis, we start by reporting the pooled estimates of life‐time rates of mental disorder in terrorist samples. We then report pooled life‐time estimates for suspected disorder, psychological problems and for any form of mental health difficulty (disorder, suspected disorders and psychological problems). Next, we report the point‐prevalence rates of mental health difficulties (i.e., ‘now’).

Finally, we present two moderation analyses. The first explores the extent to which rates of mental health difficulties are moderated by studies' source of data and the second examines type of terrorism as a moderator. The latter, in particular, is important as one of the criticisms of the literature has been the failure to disaggregate different forms of terrorism when considering the link between mental health difficulties and terrorist behaviour.

##### Lifetime rates of mental disorder

As a benchmark against which to consider the pooled life‐time prevalence rate of mental disorder we refer to Steel and colleagues' (2014) meta‐analysis of lifetime prevalence rates of mental disorder among 16‐65 year olds globally, and where included studies applied a structured psychiatric diagnostic interview. They concluded that 29.2% [95% confidence interval (CI) = 25.9%–32.6%] of people experience a mental disorder at some point in their lives. That sample was based on a pooled sample of 452,595 individuals from 82 countries.

**Figure 2:**
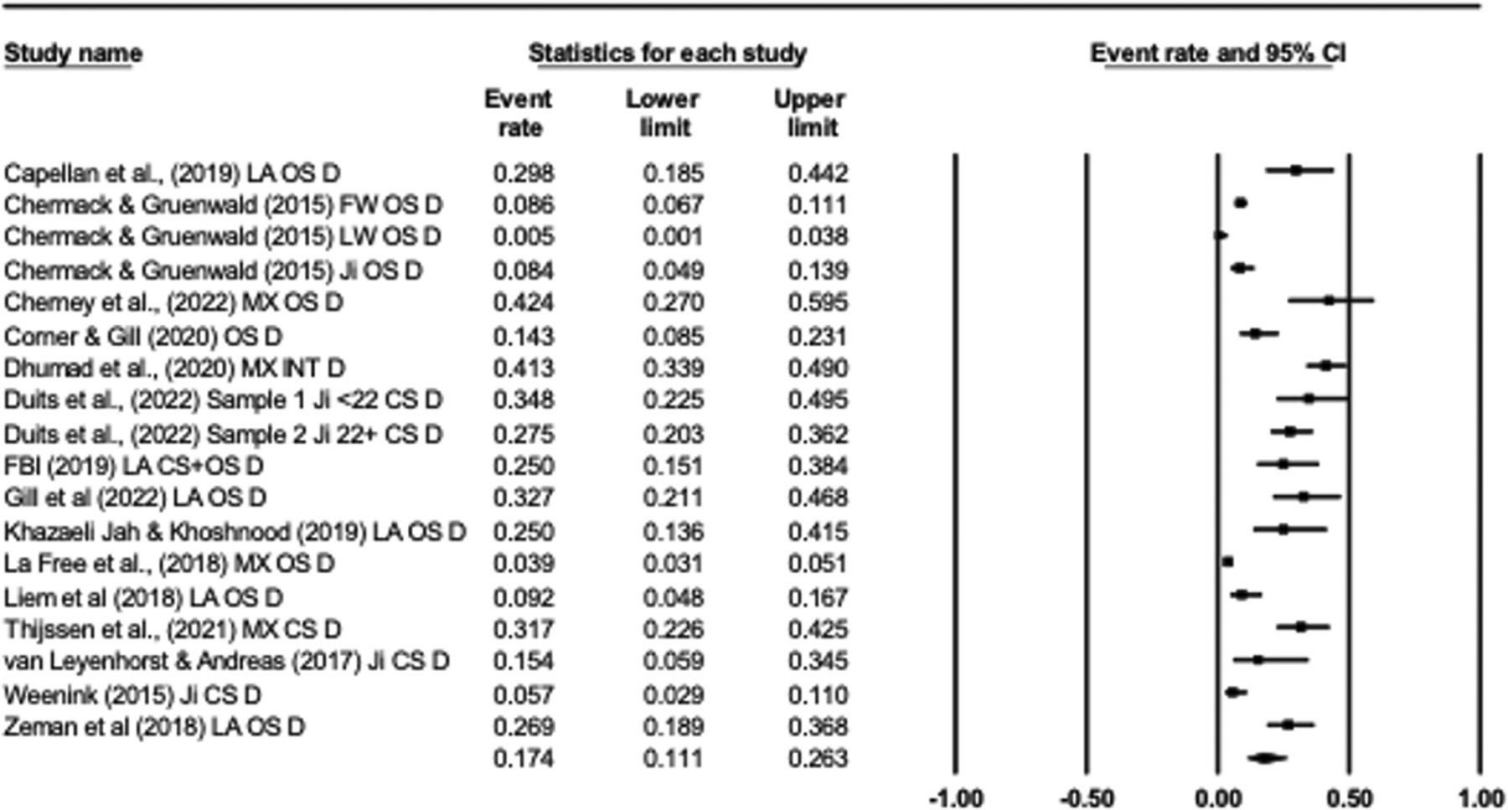
Forest plot of lifetime prevalence of mental disorders in terrorist samples

We conducted a random‐effects meta‐analyses using the total sample size for the studies (*k* = 18, *n* = 3520), regardless of type of terrorism, and with the number of positive cases of mental disorder as a proportion of all cases (i.e., denominator = all cases, including missing cases, in all samples). As anticipated, between‐study variability was high. The homogeneity test suggested that there was significant heterogeneity in the effect sizes across the included studies (*Q*_17_ = 334.4, *p* < .001) and 94.9% of the variability in effects is due to true differences across the studies rather than sampling error (*I* ^2^ = 94.9; *τ* ^2^ = 1.16). The prevalence estimates across the studies ranged from 0.5% (Chermak & Gruenewald, 2015) t0 42.4% (Cherney et al., 2022). The pooled prevalence was 17.4% (95% CI = 11.1%–26.3%) (see also Figure 2), which is lower than our benchmark from Steel et al. (2014).

We completed a sensitively analysis for the assessment of diagnosed mental disorder by first exploring the relative weights of each study, with no study responsible for more than 6.04% of the full weight and all studies contributing between 3.29% and 6.04%. As such, no study dominated the analysis. To complete the assessment we examined the pooled prevalence effects if any one study was removed from the data synthesis using the ‘one study removed’ option in CMA. As Chermack and Gruenewald (2015, 3 samples) and LaFree et al. (2018) both contribute data from large US datasets, we were particularly sensitive to the effects of removing the LaFree et al. data set. Doing so did not change the reported pooled prevalence considerably, increasing it slightly to 19.4% (95% CI = 13.5%–27.1%). Regardless of what study was removed, the point prevalence does not drop below 16.4% or exceed 19.4%.

**Figure 3:**
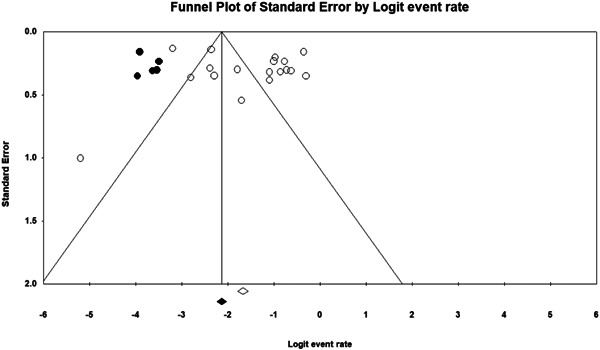
Funnel plot for publication bias of studies reporting on lifetime prevalence of mental disorder in terrorist samples

We assessed publication bias through a contour enhanced funnel plot and Eggers' regression text. The funnel plot was symmetrical, with some effects falling outside the funnel. Egger's test for a regression intercept was *p* = 0.37 (two‐tailed) suggesting no evidence of publication bias however (intercept = 2.41) even though the Trim and Fill test suggested the addition of four additional effects to be imputed (high, left and outside the funnel) to the left of the funnel to obtain symmetry in the plot (Figure 3).

##### Life‐time rates of suspected disorder

**Figure 4:**
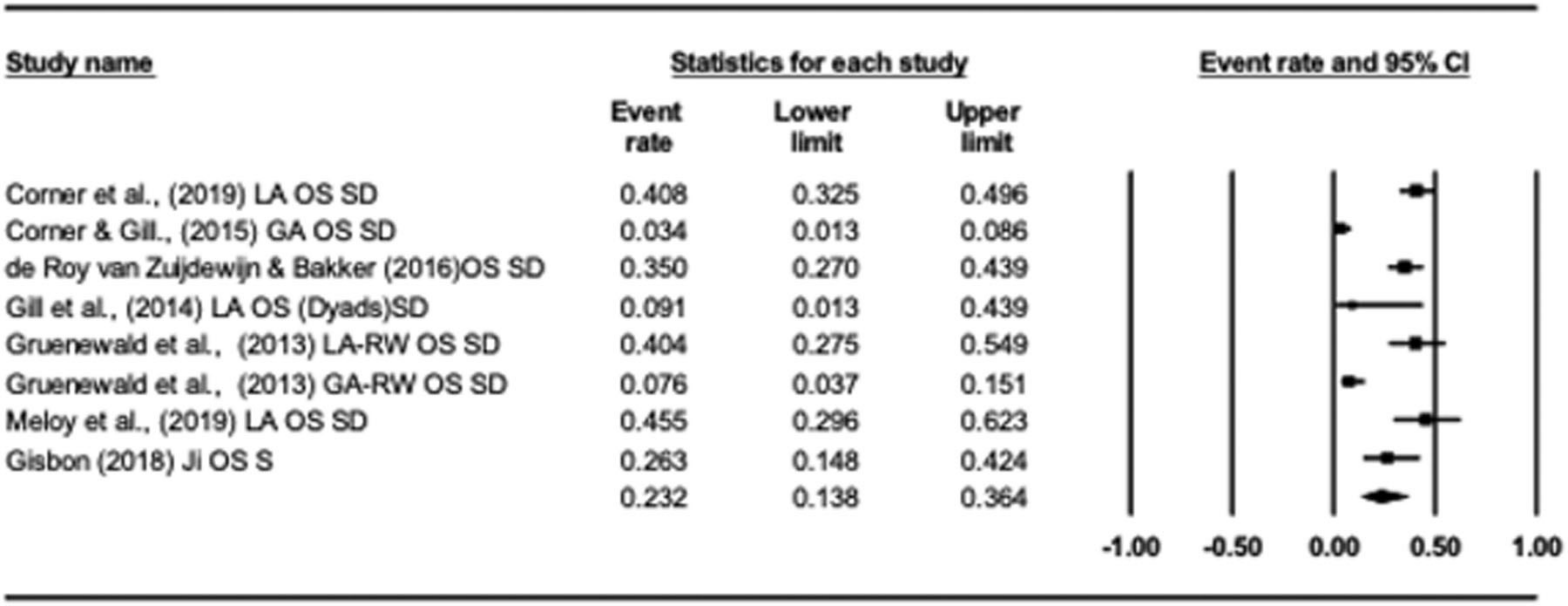
Forest plot of lifetime prevalence of suspected disorders in terrorist samples

We next replicated the analysis with studies that reported suspected mental disorder. In the data synthesis, seven papers with eight samples in total were included (*n* = 585). Again, a random‐effects meta‐analysis was run. There was evidence of heterogeneity in the effects reported across studies (*Q*_7_ = 57.69, *p* < 0.001) with almost 90% of the variability due true differences across the studies rather than sampling error (*I* ^2^ = 87.87; *τ* ^2^ = 0.68). The pooled prevalence was 23.2% (95% CI = 13.8%–36.4%), lower than the benchmark 29.2% from Steel et al. (2014) (see Figure 4). We assessed the impact of removing any one study from the analyses and noted that removing Corner and Gill (2015) led to an increase in the pooled prevalence rate to 29.5% (95% CI = 20.0%–41.2%). As the total number of studies was less than 10, in line with Cochrane guidelines, we did not assess publication bias (Page et al., 2022).

##### Life‐time rates of psychological problems

Next, we synthesised all studies and samples that reported on psychological problems. Some studies that reported diagnosed disorder or suspected disorder provided separate data for psychological problems. In such cases, we used the data relating to psychological problems. As a benchmark we draw on Winning et al. (2015) longitudinal study of psychological distress in adults from the 1958 British Birth Cohort Study, which reported that 50.7% of their sample of 6714 were distressed in childhood (24.07%, *n* = 1683), adulthood (14.97%, *n* = 1005) or both (10.68%, *n* = 717). We sought out other benchmarks but noted that psychological distress is typically measured as a point‐prevalence (‘right now’) reflecting the episodic nature of some forms of distress (e.g., following bereavement) and because the lifetime prevalence rate could be expected at close to 100%.

**Figure 5:**
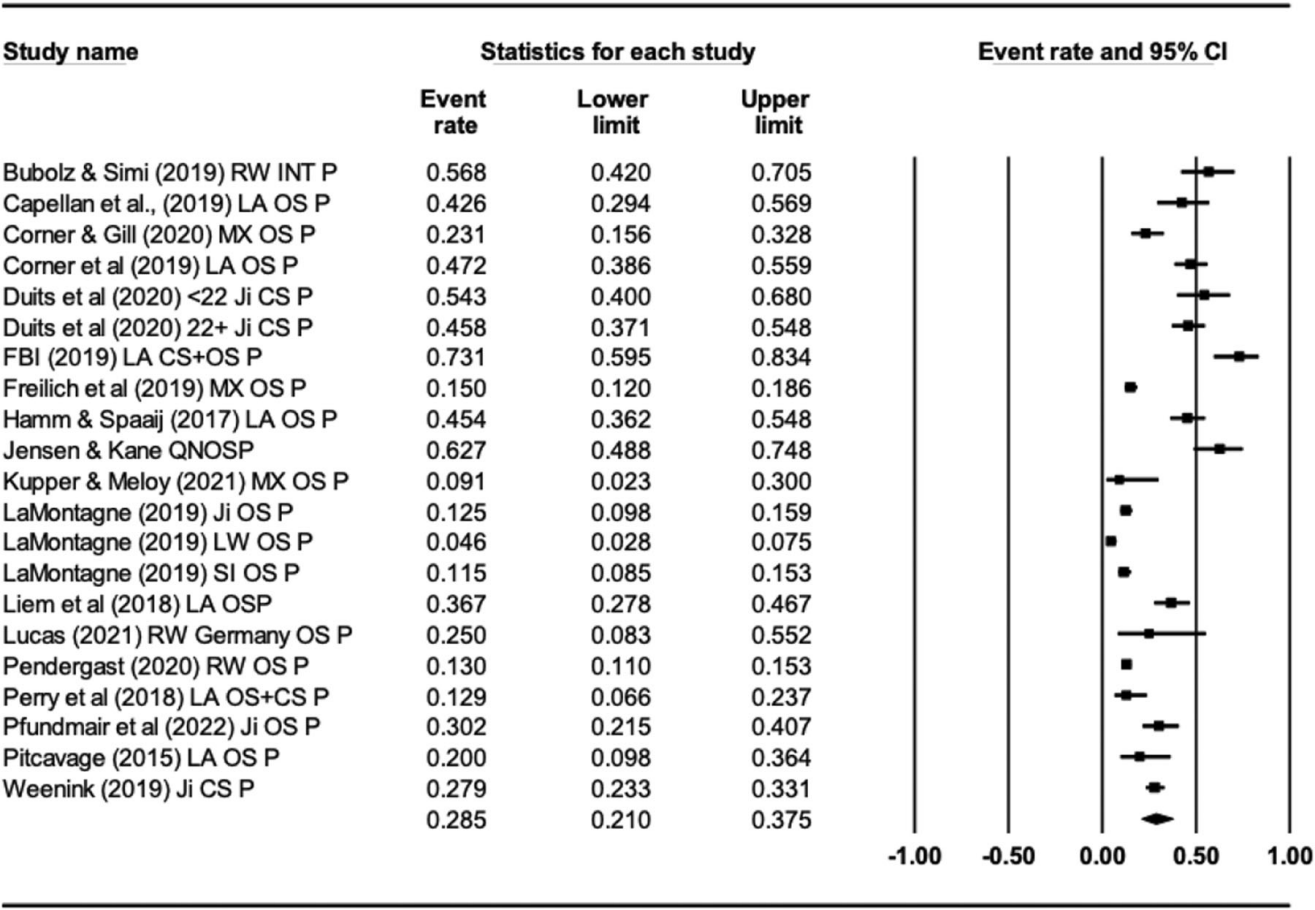
Forest plot of lifetime prevalence of psychological problems in terrorist samples

Eighteen papers with 21 samples/studies were included in the meta‐analysis (*n* = 3,806). There was heterogeneity in effects across the 21 studies (*Q*_20_ = 424.47, *p* < 0.001) with 95.3% of the variability due to true differences across the studies rather than sampling error (*I* ^2^ = 95.29; τ^2^ = 0.82). The pooled prevalence was 28.5% (95% CI = 21.0%–37.5%) which is less than both the benchmark for mental disorder and psychological distress (50.7%) (see Figure 5). Removing the FBI (2019) study from the data set reduced the pooled prevalence to 26.6% (95% CI = 19.6%–35.1%) and removing LaMontagne's (2019) left‐wing sample increased the pooled prevalence to 30.8% (95% CI = 23.0%–39.9%). Freilich et al. (2019) study used the ECDB data set and LaMontagne's (2019) three samples were based on the PIRUS data set. Omitting Freilich and colleagues' data did not change our conclusion that the pooled prevalence of life‐time rates of psychological problems does not exceed those expected in the general population (pooled prevalence = 29.4%, 95% CI = 21.3%–39.1%).

**Figure 6:**
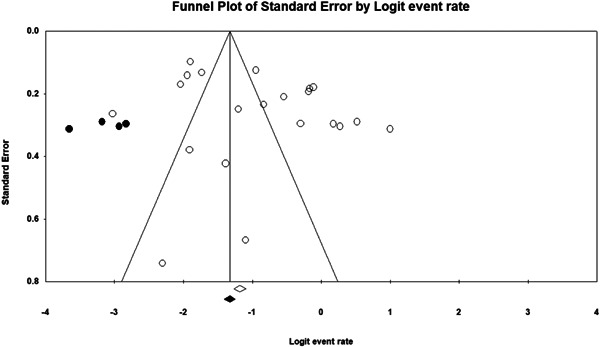
Funnel plot for publication bias of studies reporting on lifetime prevalence of psychological problems in terrorist samples

A funnel plot to assess publication bias was approximately symmetrical (Figure 6). The Trim and Fill test suggested that four inferred studies could be imputed left and outside of the funnel plot. Eggers' test suggested no evidence of publication bias however (point of intercept = 3.95, *p* = 0.09, two‐tailed).

##### Life‐time rates of any mental health difficulties

Finally, we combined all eligible studies, regardless of their reporting on disorder or problems, in one overall assessment of reported mental health difficulties in the identified literature. As some studies provide data for multiple mental health risks (e.g., disorder and problems), and other studies report different risks used overlapping datasets, we selected studies as follows. First, where one study reports two prevalence figures (e.g., psychological problems and diagnosed disorder; psychological problems and suspected disorder; suspected disorder and diagnosed disorder) we included only one effect in the synthesis with the most liberal risk selected. This meant that we prioritised risks in the following order: (1) Psychological Problems, (2) Suspected Disorder, (3) Diagnosed Disorder.

Second, if two studies reported results using overlapping datasets, but reported on different risks (e.g., study 1 = problems, study 2 = diagnosed disorder), we again prioritised problems over suspected disorder, problems over diagnosed disorder, and suspected disorder over diagnosed disorder. We do so as this strategy is most responsive to a lenient assessment of mental health issues in terrorist samples (we minimise the requirement for a clinical diagnosis).

Based on this approach, we excluded Chermack and Gruenewald's (2015) sample of right wing terrorists, for which they report mental disorder, in favour of Gruenewald et al.'s (2013) sample of right wing terrorists (for whom they report rates of suspected disorder). Both draw on the ECDB data set. Weenink (2015), which reported rates of diagnosed disorder, was displaced by Weenink (2019), which included but expanded up on their 2015 sample, but reported rates of psychological problems.

Thirty‐two papers reported on 37 samples for the analysis (*n* = 5082). Again, there was evidence of considerable heterogeneity in the data (*Q*_36_ = 548.66, *p* < 0.001) with 93% of variability in effect sizes attributable to true differences across studies rather than sampling error (*I* ^2^ = 93.44, *τ* ^2^ = 0.75). The pooled prevalence was 25.5% (95% CI = 20.2%–31.6%). Removing any one of the studies (i.e., sensitivity analysis) would not have changed the conclusion reached that the pooled prevalence of any mental health difficulty reported here does not exceed that expected in the general population. Removing the FBI (2019) study would lead to slight reduction in the pooled prevalence (24.4%, 95% CI = 19.4%–30.3%) and removing LaMontagne's (2019) left‐wing sample would lead to an increase in the pooled estimate to 26.6% (95% CI = 21.3%–32.8%). As LaMontagne's (2019) three samples derive from the PIRUS data set and Chermack and Gruenewald's (2015) two samples are based on the ECDB, we re‐ran the analysis excluding the latter. Again, there was no meaningful change in the pooled estimate (pooled prevalence = 27.4%, 95% CI = 21.8%–33.8%).

Publication bias for all studies reporting mental health difficulties (i.e., *k* = 37) was assessed through a contour enhanced funnel plot and Eggers' regression text. The funnel plot was symmetrical. A Trim and Fill test suggested the addition of one additional effect to be imputed (left of the plot) though Egger's test for a regression intercept (intercept = 1.04) was *p* = 0.47 (two‐tailed) suggesting no evidence of publication bias.

##### Moderator analysis of life‐time rates of mental health difficulties

On reviewing the eligible studies, and noting the considerable heterogeneity in all analyses, we considered potential moderators that might explain, in part at least, the evident heterogeneity. Both moderators were anticipated a priori (see Sarma et al., 2022). The first was data source. It may be the case that the heterogeneity noted in the data reflects variation in the way that different data sources capture the presence of mental health difficulties. In particular, we noted that some of the studies were based on either interviews with terrorists or closed sources (or a mixture of open and closed sources). Others were based on open source information including media reports. We therefore coded studies as Interview/Closed Source or Open Source and repeated the analysis using the data set with all studies reporting mental health difficulties to determine if the effect sizes varied across the two sets of studies. Nine studies drew on closed sources or interviews and 28 studies used open‐sources *(k* = 37, *n* = 5,082).

As evident from the Figure 5, the mean life‐time prevalence rate of mental health difficulties for samples using closed source information or interviews was 39.1% (95% CI = 28.9%–50.4%), higher than that for samples based on open source information (21.2%, 95% CI = 16.5%–28.1%). The difference was statistically significant (*Q*_1_ = 8.24, *p* = 0.004). There was evidence of considerable heterogeneity across both sets of studies (Interview/Closed *Q*_8_ = 71.76, *p* < 0.001, *I* ^2^ = 88.86, *τ* ^2^ = 0.41; Open Source Q_27_ = 379.52, *p* < 0.001, *I* ^2^ = 92.89, *τ* ^2^ = 0.72).

Next, we explored the extent to which different forms of terrorism might moderate the life‐time prevalence rates of mental health difficulties reported in the studies. Past research has proposed that there may be higher rates of mental health difficulties among lone‐actors. For this reason, we ran a moderator analysis with Lone‐Actor versus all other forms of terrorism (including mixed samples). Again, studies reporting any form of mental health difficulty were included *(k* = 37, *n* = 5082).

**Figure 7:**
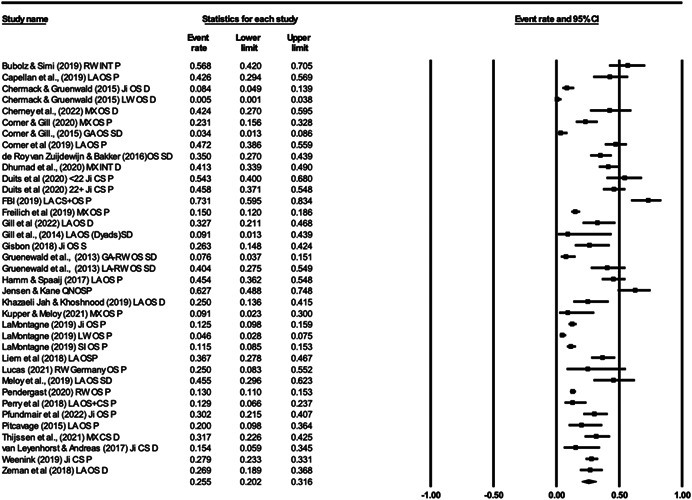
Forest plot of lifetime prevalence of any mental health problem in terrorist samples

**Figure 8:**
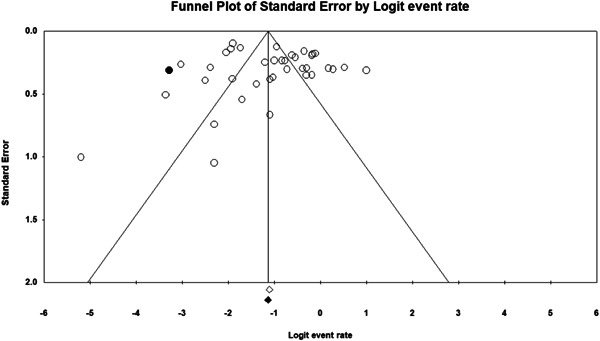
Funnel plot for publication bias of studies reporting on lifetime prevalence of any mental health difficulty in terrorist samples

**Figure 9:**
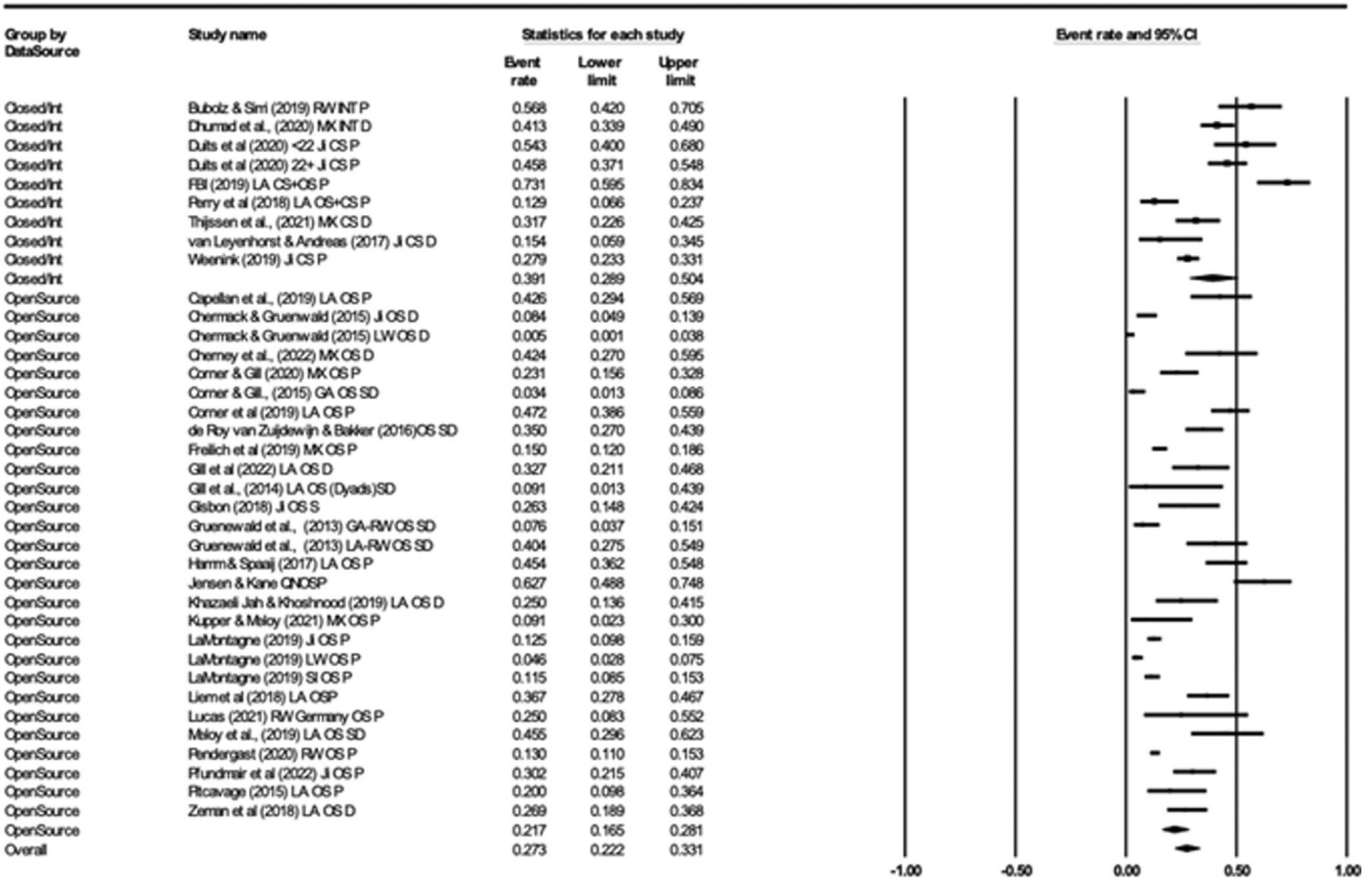
Moderating effects of data source on life‐time prevalence of any mental health difficulties in terrorist samples

**Figure 10:**
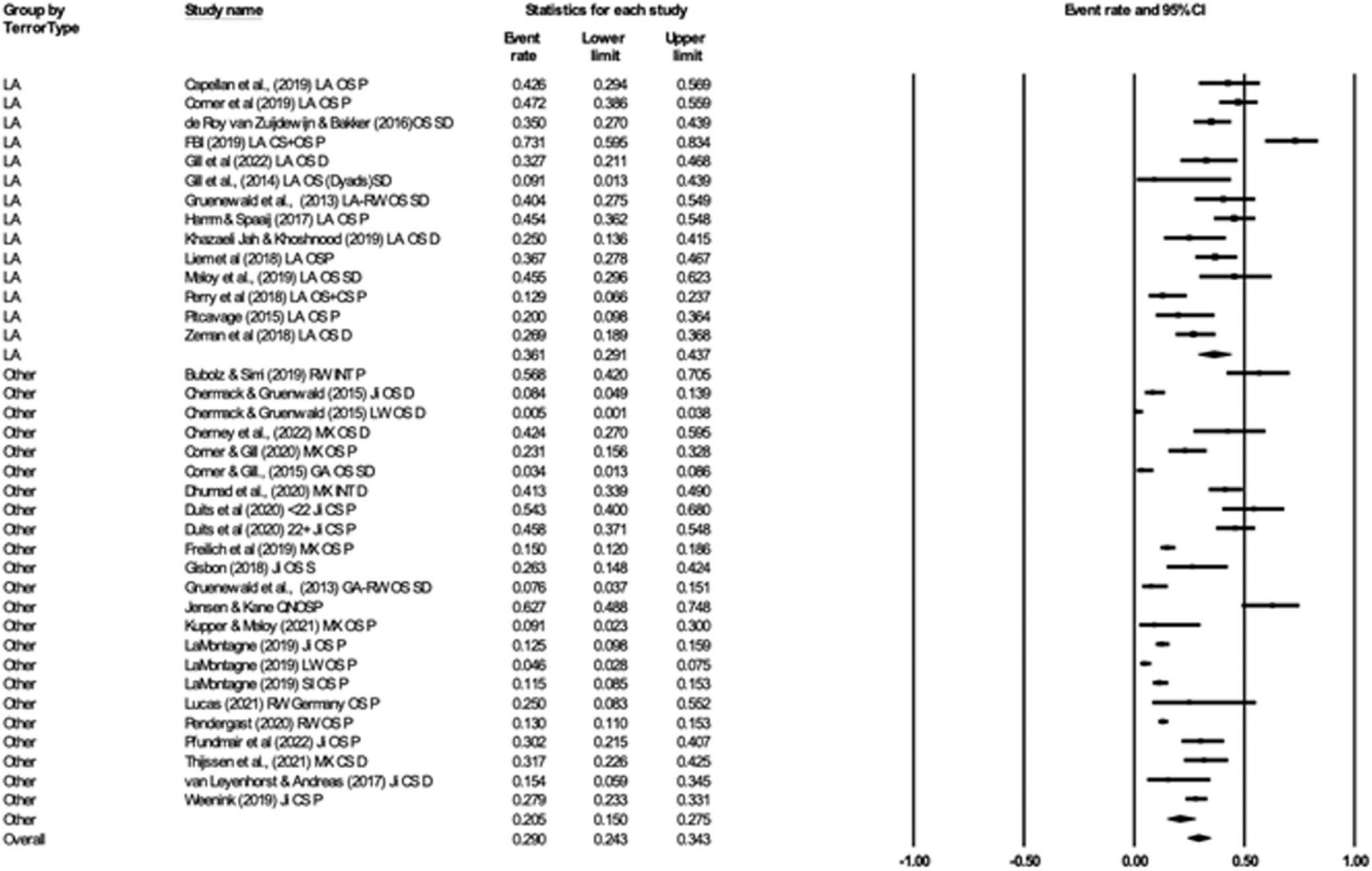
Moderating effects of terror type on life‐time prevalence of any mental health difficulties in terrorist samples

**Figure 11:**
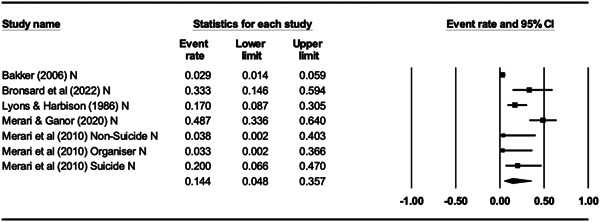
Forest plot of studies reporting point‐prevalence (now) rates of disorder or suspected disorder in terrorist samples

**Figure 12:**
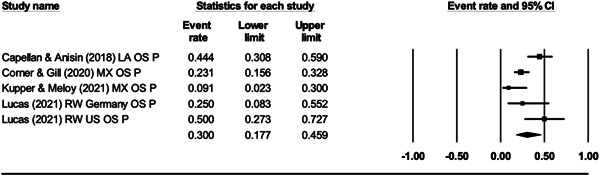
Forest plot of studies reporting lifetime rates of psychological problems where problems were onset before detection or involvement

**Figure 13:**
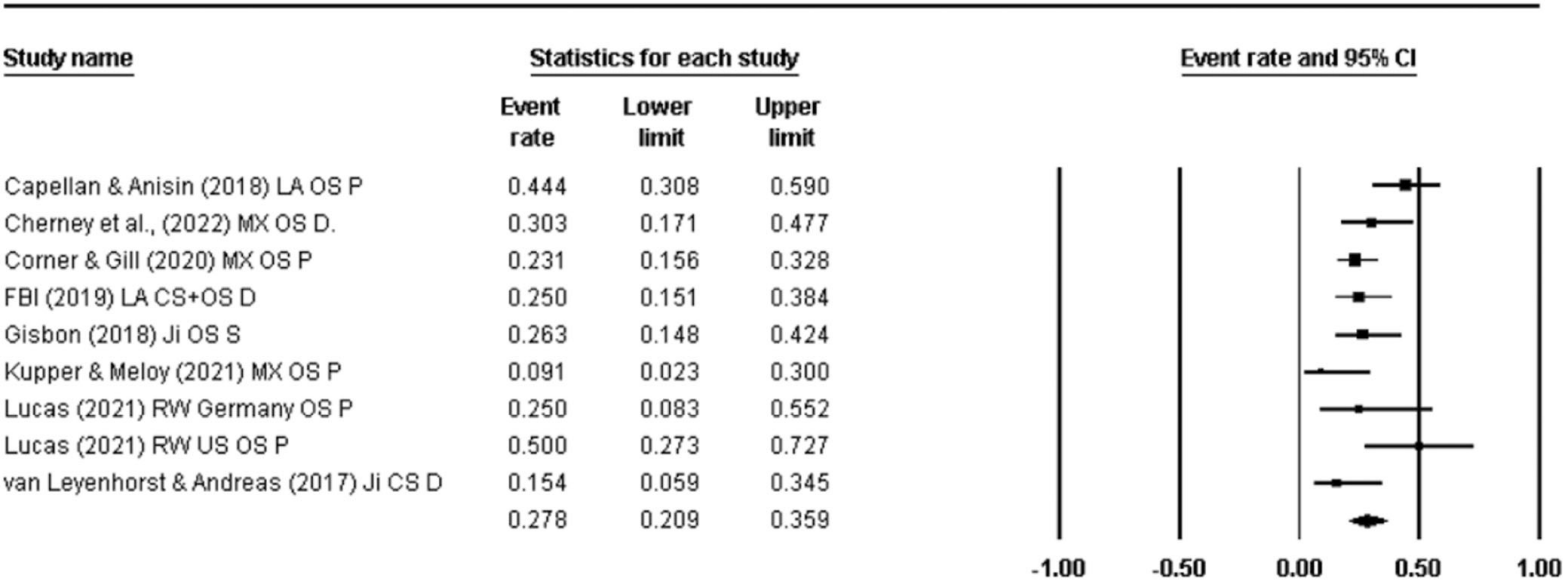
Forest plot of studies reporting lifetime prevalence rates of any mental health difficulties where these were onset before detection or involvement

Samples reporting rates for lone actors (*k* = 14) reported higher rates (36.1%, 95% CI = 29.1%–43.7%) than those reporting rates for other forms of terrorism (*k* = 23) (20.5%, 95% CI = 15.0%–27.5%). This difference was statistically significant (*Q*_1_ = 9.45, *p* = 0.002). Again, there was considerable heterogeneity across both sets of studies (lone actor *Q*_13_ = 60.74, *p* < 0.001, *I* ^2^ = 78.60, *τ* ^2^ = 0.27; Other *Q*_22_ = 376.21, *p* < 0.001, *I* ^2^ = 94.15, *τ* ^2^ = 0.75) (Figures 7, 8, 9, 10, 11, 12, 13).

##### Current mental health difficulties

Focusing on the 5 papers (7 studies, *n* = 384) reporting point prevalence of mental health diffiutlies, the pooled estimate of current mental disorder or suspected disorder was 14.4% with very wide confidence interval (95% CI = 4.8%–35.7%). There was heterogeneity evident across the studies (*Q*_6_ = 53.33, *df* = 6, *p* < .001) with 88.75% of the variability attributable to true differences in effects across studies rather than sampling error (*I* ^2^ = 88.75; τ^2^ = 2.05). None of these studies were included in the data syntheses reported earlier (i.e., analyses relating to life‐time prevalence rates). We did not assess publication bias given the low number of studies. In our sensitivity analysis we noted that the removal of Bakker (2006) led to an increase in the point‐prevalence rate to 22.5% (95% CI = 10.8%–41.0%). That study was the only study to use open‐source information in assessing diagnosed disorder and may point towards the potential for open sources to underestimate prevalence rates.

### 5.3 Objective 2—Temporality

#### 5.3.1 Included studies

Of the papers that met the eligibility criteria for Objective 1, nine papers reporting on ten studies provided data specifically relating to the proportion of a terrorist sample that had a mental disorder, suspected disorder, or psychological problems that onset either before becoming involved in terrorism or before being detected as involved (e.g., arrested for an offence). Of the studies, 8 were based on open source information (Bakker, 2006; Capellan & Anisin, 2018; Cherney et al., 2022; Corner & Gill, 2020; Gibson, 2018; Kupper & Meloy, 2021; Lucas, 2021 (2 samples), with the remainder based on closed source (including studies that used both open and closed sources) or interviews (FBI, 2019; van Leyenhorst & Andreas, 2017). Studies were based on samples of terrorists active in the US (Capellan & Anisin, 2018; FBI, 2019; Gibson, 2018), Australia (Cherney et al., 2022), Europe (Bakker, 2006; van Leyenhorst & Andreas, 2017) and globally (Corner & Gill, 2020; Kupper & Meloy, 2021). Lucas (2021) reports on terrorism in the US and Germany.

Three studies focused on Jihadi terrorism (Bakker, 2006; Gibson, 2018; van Leyenhorst & Andreas, 2017), two explored right‐wing terrorism (Lucas, 2021; both samples), two reported on lone actor terrorism (FBI, 2019; Kupper & Meloy, 2021) with the rest dealing with mixed samples (Capellan & Anisin, 2018; Cherney et al., 2022; Corner & Gill, 2020).

All studies provided data for the prevalence of mental health difficulties either before engagement or before detection for the index offence. Van Leyenhorst and Andreas (2017) report rates of diagnosed disorder before arrest. Capellan and Anisin (2018) report rates of psychological problems where those problems pre‐dated that attack leading to detection, as did Kupper and Meloy (2021) and Lucas (2021, both samples). Corner and Gill (2020) provided data directly to the authors for the proportion of their sample who reported diagnosed disorder or psychological problems before their engagement in terrorism, as did Cherney and colleagues (2022). The FBI (2019) also reports on rates of diagnosed disorder that pre‐dated involvement in terrorism. Gibson (2018) refers to the presence of psychological problems before religious conversion, and which is presumed to have occurred before becoming involved in terrorism. Bakker (2006) provides rates based on the presence of suspected disorder that at the time of arrest, measured as a point‐prevalence (‘now’).

#### 5.3.2 Risk of bias for studies included in Objective 2—Temporality

The risk of bias assessment for the studies included in our synthesis under Objective 2 was completed as part of the ROB for Objective 1 and using the JBI Checklist for Prevalence Studies.

#### 5.3.3 Synthesis of results for Objective 2

We used Capellan and Anisin (2018) rather than Capellan et al. (2019) for this analysis as the former reported data relevant to temporality where the latter does not. We also include both of Lucas (2021) samples as there neither overlap with other datasets used in the analyses. Of the 10 studies one reported current prevalence rates of suspected disorder (Bakker, 2006) and we did not include this in the data synthesis. The remaining eight papers reported life‐time prevalence rates of psychological problems (Capellan & Anisin, 2018; Corner & Gill, 2020; Kupper & Meloy, 2021; Lucas, 2021), suspected disorder (Gibson, 2018), and diagnosed disorder (Cherney et al., 2022; FBI, 2019; van Leyenhorst & Andreas, 2017) across nine samples/studies. The FBI (2019) reported rates of both psychological problems and diagnosed disorder, but does not report rates for psychological problems before involvement and so we used the rates for disorder here. Corner and Gill (2020) and Cherney et al. (2022) provided data for our analyses and data may differ from that reported in the original studies.

We calculated pooled estimates for psychological problems and all mental health difficulties in two sets of analyses. Of the five studies reporting psychological problems *(n* = 186), the pooled prevalence estimate for life‐time onset of problems before arrest/detection or involvement was 30% (95% CI = 17.7%–45.9%), and with evidence of heterogeneity across the datasets (Q_4_ = 13.20, *p* < 0.01) with almost 70% of the variability attributable to true differences in effects across studies rather than sampling error (*I* ^2^ = 69.7; τ^2^ = 0.39). For the nine studies reporting life‐time prevalence rates of any mental health difficulties before arrest/detection or involvement in terrorism *(n* = 335), the pooled estimate was 27.8% (95% CI = 20.9%–35.9%) with evidence of heterogeneity across the effects reported in the studies (*Q*_8_ = 16.16, *p* = 0.04, *I* ^2^ = 50.50; *τ* ^2^ = 0.15).

For both sets of analyses relating to temporality we conducted sensitivity analysis by inspecting the consequences of excluding one study from the data set. In neither analysis did the pooled estimate change. For the first analysis (problems, *k* = 5) the lowest estimate attainable was 25.7% (95% CI = 13.6%–43.1%; by excluding Capellan & Anisin, 2018) and the highest was 34.7% (95% CI = 21.6%–50.5%; by excluding Kupper & Meloy, 2021).

For the second analysis (difficulties, *k* = 9), the lowest estimate attainable was 25.4% (95% CI = 19.6%‐32.2%; by excluding Capellan & Anisin, 2018) and the highest was 29.3% (95% CI = 22.5%–37.1%; by excluding Kupper & Meloy, 2021). We did not assess publication due to the small number of studies in the analyses.

### 5.4 Objective 3—Mental health difficulties as a risk factor for terrorism

#### 5.4.1 Included studies

Of the papers included in our synthesis of Objective 1, 9 papers adopted a case‐control design where they compared rates of mental health difficulties in terrorist samples with rates in non‐terrorist samples (see Table 9). In one of these papers, the authors compared a terrorist sample of lone actor terrorists with three other types of lone‐actor attackers (Capellan et al., 2019). This paper draws together all data reported in earlies studies (Capellan & Anisin, 2018; Capellan, 2015) also eligible for inclusion in our narrative synthesis.

**Table 9:** Characteristics of studies included in Objective 3—Mental health difficulties as a risk factor for terrorism

Study	Jurisdiction	MHD	Data source	TS	CS1	CS2	TS (*n*)	CS1 (*n*)	CS2 (n)
Bronsard et al. (2022)	France	D—Now	Clin	Jihadi (minors)	Non‐terrorist offenders (minors)	NA	15	101	NA
Lyons and Harbison (1986)	N. Ireland	D—Now	Clin	Group Actors	Non‐political offenders	NA	47	59	NA
Kupper and Meloy (2021)	US, Finland, NZ, Canada, Europe	P—LT	OS	Ideologically motivated lone‐actors	Grievance motivated lone‐actors	NA	22	8	NA
Capellan and Anisin (2018)	United States	P—LT	OS	Lone‐actor (extremist)	Non‐extremist shooters	NA	45	261	NA
Capellan et al. (2019)	United States	P—T	OS	Lone‐actor (extremist)	Non‐extremist shooters	NA	47	272	NA
Capellan (2015)	United States	P & D—LT	OS	Lone‐actor (extremist)	Non‐extremist shooters	NA	40	242	NA
Dhumad et al. (2020)	Iraq	D—LT	I	Mixed	Murderers	Community Control	160	65	88
Liem et al. (2018)	Europe	D & P—LT	OS	Lone‐actor	Murderers	NA	98	300	NA
Horgan et al. (2016)	United States	S—LT	OS	Lone‐actor	Murderers	NA	71	115	NA

Abbreviations: Clin, study based on clinical interview; CS, comparison sample; D, diagnosed disorder; I, interview based study; LT, lifetime; OS, open source; P, psychological problems; S, suspected disorder; TS, terrorist sample.

Studies drew on samples from Europe (Bronsard et al., 2022; Liem et al., 2018; Lyons & Harbinson, 1986), the USA (Capellan & Anisin, 2018; Capellan et al., 2019; Capellan, 2015; Horgan et al., 2016) and Iraq (Dhumad et al., 2020). One study reported on samples from the USA, Finland, New Zealand, Canada and Europe (Kupper & Meloy, 2021).

Of these, five studies reported on diagnosed disorder (Bronsard et al., 2022; Capellan, 2015; Dhumad et al., 2020; Liem et al., 2018; Lyons & Harbinson, 1986), five report rates of psychological problems (Capellan & Anisin, 2018; Capellan et al., 2019; Capellan, 2015; Kupper & Meloy, 2021; Liem et al., 2018), and one reported on suspected disorder (Horgan et al., 2016).

Both Bronsard and colleagues (2022) and Lyons and Harbinson (1986) report on point‐prevalence rates of diagnosed disorder (at time of assessment) based on clinical interview. The remaining papers reporting on life‐time prevalence based on research interview (Dhumad et al., 2020) or open‐source data (Capellan 2015; Capellan & Anisin, 2018; Capellan et al., 2019; Kupper & Meloy, 2021).

In the papers, six were concerned with lone actor terrorism (Capellan & Anisin, 2018; Capellan et al., 2019; Capellan, 2015; Horgan et al., 2016); Kupper & Meloy, 2021; Liem et al., 2018), one recruited a sample of teenagers convicted of criminal association with Islamist terrorism (Bronsard et al., 2022), one recruited a sample of terrorists in Northern Ireland (Lyons & Harbinson, 1986) and one sample was described as mixed. As comparison groups serious homicide offenders or attempted murderers were used in all samples except Bronsard and colleagues (2022) who compared young people who were convicted of criminal association with terrorism with other, young offenders detained in a juvenile detention facility.

#### 5.4.2 Risk of bias

Using the JBI *Critical Appraisal Checklist for Case Control* studies (Moola et al., 2017), the nine included case‐control papers were appraised on ten criteria related to sampling, ascertainment of exposure and confounding (see Table 10). Similar to the checklist for prevalence data, a violation of one criterion (i.e., a score of ʻno’, indicating that the possibility of bias was not addressed) resulted in a ʻhigh’ risk of bias score. All case‐control studies were classified as ʻhigh’ risk of bias.

**Table 10:** Risk of bias assessment according to the JBI checklist for case‐control studies

Study	1. Groups comparable?	2. Cases and controls matched?	3. Criteria used for identification of cases and controls?	4. Reliable exposure?	5. Exposure measurement?	6. Confounds identified?	7. Strategies to deal with confound stated?	8. Outcomes assessment?	9. Length of exposure?	10. Statistical analysis	Risk of bias rating
Bronsard et al. (2022)	High	Low	Low	Low	Low	Low	Low	Low	Low	Low	High
Capellan (2015)	Low	High	Low	High	High	High	High	Unclear	Low	Low	High
Capellan and Anisin (2018)	Low	High	Low	High	High	High	High	Unclear	Low	Low	High
Capellan et al. (2019)	Low	High	Low	High	High	Low	High	High	Low	Low	High
Dhumad et al. (2020)	Unclear	Low	Low	High	High	Low	High	High	Low	Low	High
Horgan et al. (2016)	Low	High	Low	Unclear	Low	Low	Low	Low	Low	Low	High
Kupper and Meloy (2021)	Low	High	High	High	High	Low	High	High	Low	Low	High
Liem et al. (2018)	Low	High	Low	High	High	Low	High	Unclear	Low	Low	High
Lyons and Harbison (1986)	High	High	High	Low	Low	Low	Low	Low	High	High	High

Item 1 relates to the comparability of the two groups, with six studies assessed as low risk of bias and one as high. Bronsard et al. (2022) compared two groups of juvenile offenders but same sizes diverged (15 vs. 101) and it is not clear that the groups were comparable on factors other than offence type. Item 2 related to the recruitment of the two samples and the extent to which they are representative of their respective populations. Most of the studies (*k* = 7) were assessed as being at high risk of bias primarily because they sourced samples from wider populations and representativeness was not assessed. Dhumad et al. (2020) was rated as low risk as they considered sample representativeness.

Items 3 assess if the same criteria were used to identify cases and controls. Two studies were assessed as high risk on this item. Lyons and Harbinson (1986) on the basis that their samples were referred for psychiatric assessment. They clarify that the terrorist sample was referred by defence counsel, but it is not clear who made the referrals for the comparison group.

Items 4 and 5 refers to the reliability of the exposure and measurement of the exposure (i.e., mental health difficulties). In Horgan et al.'s (2016) review of open sources, two coders independently assessed the presence of the disorder and so the paper was rated as low risk for Item 5, but as ‘unclear’ for Item 4 as they report on suspected disorders (a clinical diagnosis was not required).

Items 6 and 7 assess the identification and treatment of confounding factors in the study. Seven of the nine studies clearly identified some confounding variables in their analysis including age differences (Capellan et al., 2019), gender imbalance (Dhumad et al., 2020) and coverage of data across jurisdictions (Horgan et al., 2016), however only Bronsard et al. (2022) address the confounds in their analysis.

Item 8 assess the measurement of terrorist behaviour. Here three studies were assessed as being at low risk of bias in that all samples were clearly defined and met specific criteria in terms of their offence characteristics. For example, all of those included in Capellan et al.'s (2019) comparison of terrorists and other attackers had been involved in a mass public shooting in the US between 1966 and 2017.

Item 9 relates to the duration of exposure. As the included studies measure life‐time prevalence of mental health difficulties, or clinical interviews to determine presence of difficulties, we assessed all studies as being of low risk. We also assessed the studies as being at low risk of bias in terms of their statistical analysis as in all cases appropriate analyses were reported. The minimum requirement was that studies reported proportions in each sample who had a mental health difficulty for the point or period of interest.

#### 5.4.3 Synthesis for objective 3—Mental health difficulties as a risk factor

We considered the appropriateness of presenting a data synthesis of the eligible studies for Objective 3. A particular concern is that, while all the studies used a comparison group of non‐terrorist offenders, these non‐terrorist offender samples are non‐homogenous. Dhumad et al.'s (2020) comparison group is comprised of incarcerated murderers from Iraq while Liem et al.'s (2018) comparison group were non‐terrorist homicide offenders. Other studies drew on non‐terrorist lone‐actors, described as ‘grievance motivated lone actors’ (Kupper & Meloy, 2021), ‘school shooters’, ‘rampage shooters’ and ‘disgruntled mass shooters’ (Capellan et al., 2019), non‐ideological mass shooters (Capellan, 2015) and solo mass murderers (Horgan et al, 2016). We acknowledge that this non‐homogeneity raises questions about the value of a data synthesis via meta‐analysis and so present single odds ratios for the studies.

Three studies reported point‐prevalence rates of diagnosed disorders: Bronsard et al. (2022), Lyons and Harbinson (1986), and Dhumad et al. (2020). Bronsard et al. (2022) compared point‐prevalence rates of disorder among 15 minors convicted for ‘criminal association to commit terrorism’ (p. 1) with 101 teenagers who had been convicted of non‐terrorist activities and were detained in Closed Educational Centres. The odds of the extremist sample having a mental disorder was less than that of the non‐extremist sample [odds ratio (OR) = 0.05, 95% CI = 0.01–0.18, *p* < 0.001]. In fact, the non‐terrorist sample was 20 times more likely to have a diagnosed disorder. Similarly, Dhumad et al. (2020) compared rates of Antisocial Personality (ASPD) in a terrorist sample (*n* = 160) with a sample of convicted murders (*n* = 65) in Iraq and reported that the terrorist sample was less likely meet the diagnostic requirements for ASPD (OR = 0.51, 95% CI = 0.27–0.96, *p* = .04). The sample of murderers was almost twice as likely as the terrorist sample to be diagnosed with ASPD. Lyons and Harbinson (1986) also compare point‐prevalence rates between terrorists and non‐terrorist offenders but there was a discrepancy in the reporting of the numerator for the comparison group. They report that 60 of the non‐political offenders had a mental disorder (a reported rate of 59%) but there were only 59 offenders in this sample. We did not attempt to contact the authors due to the time lapse since publication and an inability to identify an email address for either author. As we were unable to compute odds ratio for this study, we report the authors conclusion that terrorist offenders are less likely to have a mental disorder than non‐terrorist offenders, and more likely to come from a stable family background.

The remaining studies report life‐time prevalence rates of mental health difficulties. Capellan advised that the samples in their three studies overlap (Capellan & Anisin, 2018; Capellan, 2015; Capellan et al., 2019), and we, therefore, selected the most recently published study (Capellan et al, 2019). As the corresponding author provided additional data for this review (for prevalence of diagnosed disorder and psychological problems), the data reported here is not consistent with the data reported in the published journal article. In that study, the team compared ideologically motivated mass shooters with three types of non‐ideologically motivated mass shooters (school shooters, disgruntled mass shooters and rampage shooters) but, for our analyses, we compare ideologically motivated mass shooters (terrorists) with the other groups combined (i.e., non‐terrorists). We also included Kupper and Meloy's (2021) comparison of psychological problems among lone actor terrorists and grievance‐motivated lone actors, Dhumad et al.'s (2020) comparison of conduct disorder among convicted terrorists and murderers in Iraq, Liem and colleagues' (2018) comparison of psychological problems among 98 European lone actors and 300 non‐terrorist homicide offenders, and Horgan et al.'s (2016) comparison of suspected disorder across 71 lone actor terrorists and a comparison group of 115 solo mass murderers.

**Figure 14:**
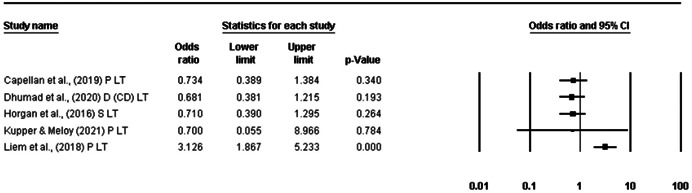
Summary of studies comparing life‐time prevalence rates of mental health difficulties in terrorist samples and non‐terrorist offender samples

The effect sizes for the remaining studies are summarised in Figure 14. Due to the variation in comparison groups, it was not considered appropriate to synthesise the effects of these studies and draw broad conclusions in relation to Objective 3. Instead, the effect sizes are displayed in Figure 14 for summary purposes only, with no pooled estimate provided. For four of the studies the ORs ranged from 0.68 to 0.73 with 95% CIs overlapping 1 (i.e., the difference in odds across the groups in these studies was non‐significant). The exception was Liem et al.'s (2018) study where the terrorist sample had a greater odds of having a life‐time history of psychological problems than non‐terrorist homicide offenders (OR = 3.13, 95% CI = 1.87–5.23, *p* < 0.001). Again, it is important to emphasise that these effect sizes should be interpreted in relation to each study's respective comparison group, rather than a homogenous ‘non‐terrorist’ sample.

In Liem et al.'s study, 37% of the terrorist sample (*n* = 36/98) had some indication of a mental illness with no missing values reported. For the common homicide offender, they report indications of mental illness for 47 cases in the sample, and which they report as 48% excluding 202 missing values (but we calculate as 15.66% based on the full sample of 300 offenders). The terrorist sample was drawn from the EU‐funded CLAT project, with the homicide offender sample drawn from the European Homicide Monitor (EHM). As such, findings may, at least in part, reflect differences in data coverage across the two data sets.

Finally, Dhumad et al. used a second comparison group drawn from the community. As all other studies used offender populations as their comparison group, it is important to distinguish it from the previous studies. The terrorist sample had greater odds of a life‐time history of childhood conduct disorder (OR = 2.24, 95% CI = 1.25–4.01, *p* = 0.007) than community controls, but the odds of a current diagnosis of ASPD did not differ across the groups (OR = 1.96, 95% CI = 0.94–4.09, *p* = 0.07).

## 6 DISCUSSION

### 6.1 Summary of main findings

The link, if any, between mental health difficulties and terrorist behaviour has perplexed researchers, policy makers and practitioners for several decades. Anecdotal evidence from end‐users would suggest that mental health difficulties do present among those who become involved in terrorism, as well as those classified as ‘at risk’ of becoming involved. This has led to the establishment of multidisciplinary teams comprised of law enforcement and health workers, with the latter expected to consider the psychological vulnerabilities of those being assessed and offer the appropriate supports (see Sarma, 2018, 2019a, 2019b). This is done with the dual intention of alleviating distress and protecting society.

Yet, across the empirical literature, evidence on the link between terrorism and mental health difficulties has been inconsistent and, at times, conflicting. This review has presented a synthesis of the body of empirical literature that has reported prevalence rates of mental health difficulties in terrorist samples (Objectives 1 and 2) and compared these rates with rates in non‐terrorist samples (Objective 3).

For Objective 1 we isolated studies reporting life‐time rates of diagnosed mental disorder, suspected disorder, and psychological problems in separate analyses. In doing so, we anticipated that we would see marked increase in rates from reported problems to suspected disorder and diagnosed disorder (we expected diagnosed and suspected disorders to be less prevalent than psychological problems). We did see such a trend, through it was less marked than anticipated.

Of the 18 studies that reported on life‐time prevalence rates of mental disorder, the pooled prevalence was 17.4% (95% CI = 11.1%–26.3%), with an increase to 23.2% (95% CI = 13.8%–36.4%) for the eight studies that reported life‐time rates of suspected disorder, and 28.5% (95% CI = 21.0%–37.5%) for the 21 studies reporting psychological problems. When we collapsed all studies reporting problems, disorder and suspected disorder into one analysis, the pooled prevalence rate for the 37 studies included was 25.5% (95% CI = 20.2%–31.6%). At the upper‐end, then, our pooled estimate of life‐time prevalence rate of mental health difficulties (Objective 1) was 28.5%.

We anticipated that isolating studies that reported prevalence rates of difficulties that emerged before involvement in terrorism would be sensitive to temporal sequencing and thus complement our test our review (Objective 2). However, the studies captured typically focused on the point of detection/arrest in temporal sequencing rather than the point of involvement. As we cannot rule out the possibility that some individuals develop mental health difficulties between the point of engagement and point of detection, our analysis is less sensitive to temporal sequencing that we had intended. Our estimates here, based on a relatively small number of studies, were 30% (*k* = 5, 95% CI = 17.7%–45.9%) and 27.8% (*k* = 9, 95% CI = 20.9%–35.9%), respectively.

In our protocol, we had envisaged adopting a range of benchmarks for disorder and problems for different jurisdictions. However, given that the range of estimates reported here are lower than anticipated, we consider it sufficient to note the following: In Steel and colleagues' meta‐analysis (2014) of lifetime prevalence rates of mental disorder among 16‐65 year olds globally, and where included studies applied a structured psychiatric diagnostic interview, they concluded that 29.2% (95% CI = 25.9%–32.6%) of people experience a mental disorder at some point in their lives. That sample was based on a pooled sample of 452,595 individuals from 82 countries. We also note that Winning et al. (2015) longitudinal study of psychological problems (distress) in adults from the 1958 British Birth Cohort Study reported that 50.7% of their sample of 6,714 were distressed at some point in their lives.

In our analysis for Objective 1 we exceeded Steel et al.'s (2014) estimate only twice. Studies that reported rates based on closed sources or interviews with terrorists reported a life‐time prevalence rate of 39.1% (95% CI = 28.9%–50.4%), though this was for any mental health difficulty (including psychological problems). Similarly, studies that reported rates for lone actor terrorists reported a life‐time prevalence rate of 36.1% (95% CI = 29.1%–43.7%), again based on any mental health difficulties. In both moderator analysis, however, the inclusion of diagnosed disorder, suspected disorder and psychological problems in the prevalence rates sets a less conservative threshold for mental health difficulties than that of Steel et al., where a confirmed psychiatric diagnosis (determined through psychiatric interview) was required.

We could also refer to other estimates, including an estimate by Carlson and colleagues (2019) which specifically estimated the current rate (i.e., point‐prevalence at any point in time) in conflict‐affected populations at 22.1%. This finding is relevant as it demonstrates that the point‐prevalence rates of mental disorder or suspected disorder identified in our review (14.4%, 95% CI = 4.8%–35.7%) is no greater than we would anticipate in the general population.

We also conducted a summary of single effects for the studies that compared terrorist samples with other samples (Objective 3). In our synthesis of five studies reporting life‐time prevalence rates of mental health difficulties, the studies trended towards terrorist samples being broadly comparable to their respective, comparison group. Two studies (Bronsard et al., 2022; Dhumad et al., 2020) provided data comparing point‐prevalence (current) rates of disorder between terrorist and non‐terrorist samples, with results from each study suggesting that the odds of the terrorist samples having a disorder was lower than that of non‐terrorist offenders. One study compared a terrorist sample with a community sample (Dhumad et al., 2020). In that study, the terrorist sample had a greater odds of having had a conduct disorder in childhood but were there was no difference in the odds of meeting the diagnostic criteria for ASPD.

In summary, our findings in relation to prevalence rates of mental health difficulties (Objective 1 and Objective 2) have provided unremarkable pooled estimates for terrorist populations as a whole, with some evidence that lone actor terrorists have higher rates of difficulties that other terrorist populations in line with earlier research (Corner & Gill, 2015; Gruenewald et al., 2013; Liem et al., 2018). It is not clear, however, whether these rates, which relate to any mental health difficulty, exceed those expected in the general population. It is important to note that the dearth of studies drawing from comparative general populations is a major limitation in the literature as a whole. Studies comparing terrorist samples with other samples have relied primarily on serious criminals as a comparison group and, perhaps unsurprisingly, do not provide evidence that mental health difficulties are associated with terrorist involvement.

At the outset of this review, we referred to the Bradford‐Hill criteria for determining causation. We viewed these criteria as important in the context of terrorism research because they point to the kinds of evidence that one would expect to see if there was a causal link between the risk and the outcome, even in the absence of prospective longitudinal case‐control studies. Here we suggested that if there was a causal link between mental health difficulties and terrorism, then we could reasonably expect to see higher prevalence rates of difficulties in terrorist samples than the general population or a comparison group (prevalence), and that this would be particularly marked where studies were sensitive to temporal sequencing (temporality). The evidence from this review did not support either criterion. As discussed further below, this may reflect the methodological limitations of the body of evidence and the narrow scope of the review.

The review was also interested in the Bradford‐Hill criterion of plausibility – that there should be a strong theoretical link between the risk and outcome. We considered this as part of our Risk of Bias assessment. In general, there is a sense of convergence in the literature included in the review where authors are arguing that the link between mental health and terrorism is (a) complex, (b) indirect and involving interaction with other psychosocial experiences and (c) highly individualised (e.g., Corner et al., 2019). At most mental health difficulties present a background non‐determinative predisposing factor nested among other factors and that may present a vulnerability to terrorism through the depletion of resilience, impairment of judgement, restriction of opportunity, and an almost unlimited range of other life experiences and situational factors. Such complexity is not uniquely a feature of trajectories into terrorism—it characterises almost all forms of serious offending where no single risk factor or process is determinative.

Bubolz and Simi (2019) illustrate this approach to formulation in their examination of mental health problems among former members of white supremacist groups, suggesting that these psychological problems may be rooted in traumatic experiences, often during childhood and adolescence and compounded by experiences of rejection in adulthood. Ultimately this may create a susceptibility to being drawn into extremist groups. Jensen and Kane (2021) reach a similar conclusion based on their research with QAnon followers as do Cherney and colleagues (2022) following their analysis of young people who radicalised to violence in Australia. They suggest that background predisposing factors like mental health difficulties and ‘deviant conduct’ interact with exposure to radicalising content and networks to ‘set up a potential pathway to violent extremism’ (p. 112). Others observed interactions between mental health difficulties and past non‐terrorist criminality (Thijssen et al., 2021), exposure to military‐related trauma (Haugstvedt & Koehler, 2021) and grievances such as poverty and oppression (Candilis et al., 2021).

With one exception, studies included in the review didn't propose that mental health difficulties precipitated terrorist involvement. Clemmow et al. (2020) do appear to allude to such a process however. They viewed lone actor terrorism as heterogeneous and develop a typology of lone‐actor violence based on person‐exposure patterns. Of four types that they isolate, one is labelled ‘susceptible’ and, according to the authors, may radicalise quickly due to high impulsivity and psychiatric disorder which presents a ‘core cognitive susceptibility to environmental influence’ (p. 468).

The possibility that mental health difficulties are protective against terrorist involvement was also considered in the studies, with most echoing what King et al. (2018) refer to as a selection effect, whereby terrorist organisations prefer recruits who can be trusted, are reliable and can take direction. This said, Bubolz and Simi (2019) found that white supremacist organisations are open to individuals with mental health problems and, in contrast with other forms of terrorism, ‘may actually prefer’ (p. 10) to recruit such individuals (p. 19).

In summary, the findings across our three review objectives converge with the theoretical positions set out in the papers. We don't see indications/tentative evidence of a causal relationship between mental health difficulties and risk of engagement in the pooled prevalence rates. Instead, our authors are arguing for more nuanced ways of thinking about the role mental health may play in the lives of those who may be at risk of involvement in terrorism.

### 6.2 Overall completeness and applicability of the evidence

We searched a broad range of platforms and databases, unrestricted by year of publication. Anticipating that some studies reporting relevant data would not have mental health as a primary focus, and thus may not refer to mental health in the title or abstract, we also completed an extensive supplementary search of the grey literature, contacting leading authors and expert networks, by hand searching specialist journals and by harvesting records from included studies. We believe that we have captured the bulk of studies that meet our eligibility criteria.

Clarification or additional data was requested from corresponding authors representing more than half of all included studies. This aided our decision making with regard to prioritising and excluding non‐independent studies (overlapping data set). It also helped us determine how authors managed missing data and allowed us to standardised our approach to calculating effect sizes (i.e., by counting positive cases as the numerator and all cases as the denominator).

Despite these strengths, we would acknowledge that our review had a number of limitations which hampers its applicability. In particular, we expect that there are additional, unpublished and published studies that we have not captured in our search, and acknowledge that the exclusion of studies not published in English undermines the overall completeness of the review. We also acknowledge that our review was narrow in scope and that there are other sources of evidence, including qualitative studies, that offer an equally valuable, and complementary, lens through which to explore the role of mental health difficulties in terrorism.

### 6.3 Quality of the evidence

Notwithstanding the barriers to conducting research with terrorist samples, and the high threshold set by the JBI checklists deployed in our risk of bias assessment, there were a number of limitations in the body of evidence included. We highlight three here. First, the majority of studies included were based on open‐source datasets that are almost entirely reliant on media and court reporting. While such datasets are valuable for dealing with many research questions, we would question to what extent open sources accurately capture the presence and absence of mental health difficulties and, by extension, the validity of interpretations of disorder or clinical psychological problems by researchers or clinicians based on such data.

Bakker (2006) captures the limitation of open sources well, reporting that ‘it is impossible to gather complete information from open sources for all variables… This means that our generalisations sometimes required some crude judgement on our part’ (p. 43). Others have been more vocal in their concerns, with Brym and Araj (2012) referring to researchers making inferences as to the characteristics of terrorists based on ‘scraps of biographical evidence pasted together from newspaper reports and other published sources’ (p. 433) and Kupper and Meloy (2021) acknowledging that their own source of data (manifestos) offered ‘very limited ability’ to identify the presence or absence of mental health difficulties (p. 14). Conversely, whilst acknowledging the limitations of open sources in terrorism research, Clemmow and colleagues (2020) note that systematic approaches to trawling open sources, extracting data and coding that data can mitigate against some concerns. Similar defences have been mounted by others (e.g., Cherney et al., 2022), and we see evidence of this rigour in most of the studies included in this review. The detailed accounts of the processes used to build the datasets in the eligible studies would suggest a more systematic process than suggested by Brym and Araj.

Some have argued that one way of overcoming this limitation is through in‐depth research with the terrorists themselves (e.g., Bubolz & Simi, 2019), an approach adopted by a number of studies included in this review. However, even here there have been concerns about the reliability of data based on retrospective recall of life experiences (Simi et al., 2016).

A second limitation of the evidence base is uncertainty as to the representativeness of the samples recruited, with many of the authors of the papers included here acknowledging that their findings may not generalise to other groups within their jurisdictions or apparently similar forms of terrorism in other parts of the world (e.g., Candilis et al., 2021; Pfundmair et al., 2022).

Third, few studies considered specific diagnoses and links to terrorism risk (for exceptions see Bronsard et al., 2022; Thijssen et al., 2021). The potential value of doing so is illustrated by Thijssen et al.'s (2021) study with incarcerated terrorist offenders. Having reported rates of diagnosed disorders that are in‐line with that expected in the general population, they then noted that rates of schizophrenia and autism spectrum disorder (ASD) were higher than expected. With regard to ASD, they suggest that individuals with ASD may have social and communication deficits and be detail‐orientated, both of which may attract them to on‐line environments and extremist content.

Fourth, we believe it is important to highlight the two different approaches to treating missing values adopted in the studies included and the consequences for reported effect sizes. Some papers based prevalence estimates for mental health difficulties based on positive cases against all cases for which the mental health status was known (i.e., confirmed positive or negative cases, but excluding unknown cases, and sometimes referred to as ‘valid precent’). Others reported rates based on positive cases against all cases. Our synthesis of all studies was based on the latter approach.

To illustrate the consequence of these divergent approaches it is worth considering LaFree and colleagues (2018) study of 1473 extremists in the US and based on open‐source data (where significant levels of missing values for mental health difficulties might be expected). Researchers were instructed to code disorder as a missing value if there was no reference to mental disorder in the sources. In their main analysis they report that this left them with 284 individuals for whom they had data, and 43% of whom had a mental illness. However, elsewhere they note that if they include all cases in the denominator, then the ‘effective rate of mental illness in the data set drops to 8.4%, which is comparable to rates of mental illness in the general population’ (p. 246).

Divergent approaches to managing missing data is not in itself an issue of quality (just reporting standards). What would improve the quality of the data, however, is consistent analysis of missing values to assess bias. Few studies included in the review presented such an analysis, though there are notable exceptions (e.g., Bronsard et al., 2022).

### 6.4 Potential biases in the review process

Our exclusion of studies that were not published in English (e.g., Alberda et al., 2018) introduces a potential source of bias in the review. It is hard to predict what effect this exclusion may have had on our synthesis. One review of 50 meta‐analyses concluded that when non‐English language papers were included in those reviews the estimates reported in the meta‐analyses changed, but in inconsistent directions (Neimann Rasmussen & Montgomery, 2018). In five studies, the estimates became more positive and, in 16, the estimates became less positive. While acknowledging that there can be significant resource requirements to running searchers in multiple languages (and screen and extract (reliably) data from those records), the authors concluded that ‘non‐English studies are important to include to avoid bias in reviews’ (p. 4).

Second, we did not screen titles and abstracts in duplicate which has been found to introduce biases. Waffenschmidt et al. (2019) presented a synthesis of papers that compared the reliability of single versus conventional double screening in systematic reviews. They noted that even among experienced reviewers, the median proportion of missed records was 3% when not screened in duplicate. In the current review, the collective experience of the reviewers, as well as time constraints associated with the large number of records returned from our search, informed our decision not to screen the titles and subtracts in duplicate. SC and KC are experienced reviewers with content expertise. They have screened for Campbell Collaboration and Cochrane reviews in the past, including one review in the area of terrorism where they screened together (Carthy et al., 2020). SC has a PhD in the area of psychology and terrorism and KC is a forensic psychologist who has co‐authored reports with KS on the link between mental disorder and terrorism.

Third, two key decisions made by the research team shaped the review and analyses. First, we selected studies that met our conceptualization of terrorism. In the main this was uncomplicated, as the samples recruited engaged in behaviours that could be broadly considered as terrorism. However, this was not always the case. For example, we included Jensen and Kane's (2021) QAnon sample in our review. The sample is a sub‐sample (*n* = 51) of QAnon followers and excludes those involved in the Capital Hill riots on January 6th, 2021. The QAnon movement is viewed as an emerging and evolving threat by the US authorities who have expressed concern that more followers will move from radical on‐line behaviour (‘digital soldiers’) to violence. However, some have cautioned that most followers pose little threat of violence and that researchers run the risk of exaggerating the threat they pose by conflating radical thought and radical action (Moskalenko & McCauley, 2021). Others may have excluded this sample. In this case, we sought to be inclusive and selected the study as being eligible for our review.

Fourth, we classified studies as reporting on mental disorder, suspected disorder and psychological problems. We have sought to make this process rigorous and transparent in our reporting but recognise that we could have set more stringent requirements in classifying a study as reporting on diagnosed disorder by requiring a confirmed clinical assessment. We could also have set a less stringent requirement by not disaggregating those with diagnoses and suspected diagnoses. Others may have approached this differently.

Finally, we acknowledge the importance of moving beyond data based on general terrorist samples towards a more nuanced assessment of rates of mental health difficulties across different forms of terrorism. In our disaggregation of forms of terrorism in our data synthesis, we compared studies that reported on lone‐actor terrorist samples with studies reporting on all other terrorist samples. However, as the literature base builds, future syntheses will be in a position to disaggregate the analyses further and, thus, consider rates of mental health difficulties across different forms of terrorism (e.g., Jihadi vs. lone actor vs. right‐wing vs. separatist, etc.).

### 6.5 Disagreements or agreements with other studies or reviews

Our conclusion that the evidence reporting period and point prevalence of mental health difficulties does not support the mental health—terrorism hypothesis is in‐line with much of the literature. Our estimates differ from an earlier review, but not remarkably so. Gill et al. (2021) completed a review and meta‐analysis of 19 studies that reported rates of mental disorder in terrorist samples. They reported a pooled prevalence rate of confirmed diagnosis of 14.4%, with higher rates for studies using closed data (16.96%) than those using open sources (9.82%). They also noted that studies comparing lone actors with other terrorist samples reported higher rates in the former than the latter. They conclude that a) the evidence does not support the presence of e a unique profile of psychopathological traits that may leave an individual vulnerable to radicalisation but b) lone‐ and group‐terrorists may appear to be two distinct groups of people in terms of their drivers and criminogenic needs.

Our review reports higher rates of mental disorder than reported in Gill and colleagues' (2021) synthesis and observes the same trend with regards of lone actors compared to other forms of terrorism. The higher rates reported here may reflect a more inclusive approach where we permitted terms like ‘confirmed diagnosis’, and reference to ‘disorder’ and diagnostic criteria as evidence that authors were counting medically diagnosed disorders.

### 6.6 Authors' conclusions

Based on our synthesis of this evidence base, the review does not lend support to the assertion that terrorist samples are characterised by higher rates of mental health difficulties than that observed in the general population. Accepting that there are methodological limitations in the studies extracted, the pooled life‐time prevalence estimate for mental disorder is close to the prevalence rate seen in the general population and the confidence intervals of that estimate are within the range expected for the general population. The exception may be lone actor terrorism, where rates were significantly higher than all other forms of terrorism combined. However, even here the rates are broadly in line with what is expected in the general population.

Findings and conclusions are tentative, reflecting limitations in the body of evidence. We do not discount the possibility that as more evidence emerges our understanding in the area will evolve and conclusions may change. We accept that regardless of findings deriving from group‐based statistics, mental health problems may emerge as relevant in an individual case when that individual is understood in context, as part of a broader psychosocial picture. This is in line with current thinking in the area (e.g., Thijssen et al., 2021).

### 6.7 Implications for policy and practice

In considering the implications of our review findings for policy and practice we are acutely aware of the narrow focus of our review, and issues with the reliability and validity of the body of evidence as a whole. We also acknowledge that in restricting the review to prevalence and case control studies we have taken just one of many lenses that could be used to consider the mental health—terrorism hypothesis, and that there is equally important social scientific literature that adopts qualitative approaches. These too provide valuable context and insights in the area. Against the backdrop of these limitations, we tentatively suggest that following implications of our findings.

The first implication relates to the focus of the debate around mental health and terrorism among policy, practice and research stakeholders, and which seems to all‐too‐frequently view mental health difficulties as a causal risk factor for violence and assume that if we could only deal with these mental difficulties we would see a reduction in risk. This case is not supported in the evidence reviewed here because there is little evidence from prevalence studies that mental health problems are more prevalent in terrorist samples than in the general community (terrorism *in all its forms*, as opposed to specific forms of terrorism). We note the emerging consensus that mental health difficulties are one of many indirect non‐causal predisposing risk factors for terrorist involvement just as such difficulties are a background predisposing risk factor for a whole host of other negative outcomes in life including delinquent and offending behaviour (e.g., Malvaso et al., 2016), interpersonal violence (Coid et al., 2021) and cardiovascular disease (Suls & Bunde, 2005).

There may be exceptions for specific forms of terrorism including lone actors, or specific sub‐types of lone actors. This points to the importance of disaggregating terrorism into more granular sub‐types and continuing to seek out any evidence that mental health difficulties play a more direct or determinative role in specific forms of terrorism (e.g. Gill & Corner, 2017). Until such a case is made, however, we should neither assume that a more direct link exists nor discount that it may.

If it is accepted that the best evidence points towards mental health difficulties as a being an indirect background predisposing risk factor for terrorism involvement, then there are some obvious implications for both risk assessment of those believed to be at risk of terrorism involvement/recidivism and for broader preventative measures. Mental health should remain a focus of risk assessment. However, the focus should not be just on a possible direct link between a mental disorder and violence propensity (not supported by the evidence reviewed here), but also the implications of such difficulties for management and supervision (e.g. engagement and compliance with interventions to reduce risk), homelessness, social isolation, personal support, stress and coping, employment and a host of other difficulties that are associated with psychological problems and also offending behaviour. This observation resonates with the conclusions reached by authors of the studies included here who argue in favour of non‐causal links between mental health and terrorism (e.g., Bubolz & Simi, 2019; Jensen & Kane, 2021). It also resonates with the broader practice of forensic risk assessment, and central to popular risk assessment systems including the HRC‐20 (Douglas et al., 2013).

That rates of mental health difficulties are in line with those in the general population suggests that there is value in considering public health primary prevention approaches to countering terrorism which has been recommended by others (e.g., Bhui & Jones, 2017). Here the focus is upstream of the problem of concern, where resources are invested in communities to bolster resilience and access to healthcare etc., to reduce rates of psychological problems or ability to cope with problems and with the potential consequence that communities become resilient to crime in all its forms.

### 6.8 Implications for research

Despite a relatively large and growing literature on terrorism and mental disorder, it remains an area of research that is difficult to expose to academic enquiry. The barriers to conducting this type of research are many and varied. Indeed, the limitations identified in the evidence‐based are predominantly born out of these challenges, rather than any oversight on the part of researchers. This said, the review has highlighted a number of key limitations in the literature on terrorism and mental disorder that can serve to either, (a) enhance our ability to critical evaluate research findings or (b) improve the way we do research in the first place. These follow from the conclusions of our Risk of Bias assessment and discussion of the quality of the evidence encountered during this review.

First, databases constructed from open sources such as media reports and court reports, present a readily available source of potentially rich data on the characteristics of terrorists and their behaviours. However, the value of open sources in terms of classifying cases as either ‘having’ or ‘not having’ a diagnosis of a mental disorder is entirely contingent on such information being made open‐source in the first place, as well as its accuracy. There is a need to complement studies using such databases (which represented the majority of our studies), with studies that use alternative sources of data. These alternatives will also have strengths and limitations, but collectively the findings from studies incorporating different methodologies allow us to measure the phenomenon through different tools, thus having more confidence in their generalisability.

Second, there is a need for some reporting standards to be adopted in the field. The review raised concerns about the treatment of missing values and the potential for the exclusion of missing cases from the denominator in calculating prevalence rates to dramatically inflate these rates. At the same time, including missing cases may serve to artificially decrease the prevalence rates. The obvious solution is to report both prevalence estimates as a standard, highlight the difference between the two and analyse any bias in patterns of missing data.

Third, we will need increasingly nuanced research to explore non‐causal processes that may lead to terrorism. Research needs to increasingly disaggregate terrorism into all its forms and sub‐forms, and also consider the links between specific forms of mental health difficulties and terrorist behaviour. In this way, disaggregation of both risk and outcome are required. Although we have observed the disaggregation of mental health difficulties into formal diagnoses and other, less clinical constructs (e.g., psychological problems), disaggregation of the former is lacking, limiting our ability to understand the nature of the relationship between specific, types of disorder and terrorism involvement. In terms of the outcome, we have observed some disaggregation. However, to date, studies have relied primarily on serious criminals for comparison groups and, perhaps unsurprisingly, do not provide evidence that mental health difficulties are associated with terrorist involvement

## CONTRIBUTIONS OF AUTHORS

*Content*: Kiran Sarma, Sarah Carthy and Katie Cox

*Information retrieval*: Sarah Carthy, Katie Cox and Kiran Sarma

*Statistical analysis*: Kiran Sarma

*Systematic review methods*: Kiran Sarma, Sarah Carthy and Katie Cox

## SOURCES OF SUPPORT

This review was supported by the DHS Science and Technology Directorate and the Five Research and Development (5RD) Countering Violent Extremism Network. This study also received support by the European Commission's Horizon 2020 programme, Grant No. 699824.

## DECLARATIONS OF INTEREST

The authors declare no conflict of interest. While the authors have been involved in the development of related research, no studies published by the authors are included in this review. The authors previously published the preliminary results of part of this study elsewhere.

## PLANS FOR UPDATING THE REVIEW

The corresponding author intends to update the review every 5 years.

## Supporting information

Supporting information.
