# Supplementary material for: Mental disorder, psychological problems and terrorist behaviour: A systematic review and meta‐analysis
Source: Campbell Syst Rev. 2022 Aug 10;18(3):e1268. doi: 10.1002/cl2.1268 (PMC9364674; doi:10.1002/cl2.1268)
Supplement: Supplementary file 1 — Supporting information. [file CL2-18-e1268-s001.docx]

## Appendices

Appendix A: Search record

Appendix B: Determination of independent findings

Appendix C: Coding form

**APPENDIX A. Search Record**

| **Database** | **Platform** | **Search Date** | **Coverage Dates** |
| --- | --- | --- | --- |
| Criminal Justice Database | ProQuest | 6/04/2022 | 1981-present |
| Psychology Database | ProQuest | 6/04/2022 | Varies by indexed title |
| Political Science Database | ProQuest | 6/04/2022 | 1985-present |
| Public Health Database | ProQuest | 6/04/2022 | Varies by indexed title |
| PTSDpubs | ProQuest | 6/04/2022 | 1871-present |
| Social Sciences Database | ProQuest | 6/04/2022 | Varies by indexed title |
| Sociology Database | ProQuest | 6/04/2022 | 1985-present |
| Sociological Abstracts | ProQuest | 6/04/2022 | 1952-present |
| Social Services Abstracts | ProQuest | 6/04/2022 | 1979-present |
| International Bibliography of Social Sciences | ProQuest | 6/04/2022 | 1951-present |
| Dissertations and Theses Global | ProQuest | 6/04/2022 | 1637-present |
| PsycArticles | Ovid | 5/04/2022 | 1908 to March 14, 2022 |
| PsycExtra (grey literature) | Ovid | 5/04/2022 | 1908 to March 14, 2022 |
| PsycInfo | Ovid | 5/04/2022 | 1806 to March Week 4 2022 |
| Embase | Elsevier | 6/04/2022 | 1947-present |
| Social Sciences Citation Index (SSCI) | Web of Science | 6/04/2022 | 1956-present |
| Conference Proceedings Citation Index- Social Science & Humanities (CPCI-SSH) | Web of Science | 6/04/2022 | 1996-present |
| Book Citation Index - Social Sciences and Humanities (BKCI-SSH) | Web of Science | 6/04/2022 | 2005-present |
| Emerging Sources Citation Index (ESCI) | Web of Science | 6/04/2022 | 2017-present |
| Scopus | Elsevier | 6/04/2022 | 1788-present |
| PubMed | NIH Library of Medicine | 6/04/2022 | 1966-present |
| Criminal Justice Abstracts | EBSCOhost | 5/04/2022 | 1968-present |

### *ProQuest Databases*

| Set# | Syntax | Databases |
| --- | --- | --- |
| S1 | noft(worr* OR afraid OR fear* OR unhapp* OR "sad" OR sadness OR emotion* OR quiet* OR withdraw* OR guilt* OR worthless* OR suicid* OR harm* OR mood* OR affect* OR addict* OR neuro* OR intellect* OR autis* OR "ASD" OR ADHD OR motor OR schizo* OR bipolar OR bi-polar OR phobi* OR panic OR OCD OR obsess* OR trauma* OR PTSD OR attach* OR dissociat* OR somatic OR eating OR anorexi* OR sleep* OR insomni* OR compulsi* OR dysphori* OR oppositional OR conduct OR antisocial OR anxious* OR anxiet* OR alcoholi* OR depressed OR depression OR depressive OR stress OR stressor* OR stressful) OR noft(psych* NEAR/3 (disorder* OR symptom* OR illness* OR "ill")) OR noft(mental* NEAR/3 (disorder* OR symptom* OR illness* OR "ill")) OR noft(alcohol NEAR/3 (disorder* OR misuse OR "use" OR user OR users)) OR noft(drug* NEAR/3 (disorder* OR misuse OR "use" OR user OR users)) OR noft(substance* NEAR/3 (disorder* OR misuse OR "use" OR user OR users)) OR noft(learning NEAR/3 (disorder* OR impair*)) OR noft(attention* NEAR/5 (disorder* OR symptom* OR impair*)) OR noft(communication* NEAR/3 (disorder* OR symptom* OR impair*)) OR noft(cognitive* NEAR/3 (disorder* OR impair*)) OR noft(personality* NEAR/3 (disorder* OR symptom*)) | Criminal Justice Database, International Bibliography of the Social Sciences (IBSS), Political Science Database, ProQuest Dissertations & Theses Global, Psychology Database, PTSDpubs, Public Health Database, Social Science Database, Sociological Abstracts, Sociology Database |
| S2 | noft(terror* OR extremism OR extremist* OR "radical" OR radicals OR radicali* OR "political violence" or "lone actor*" or lone-actor* or Jihadi or Salafi or "right wing" or right-wing or separatist* or nationalist* or religio* or guerrilla* or paramilitary) | Criminal Justice Database, International Bibliography of the Social Sciences (IBSS), Political Science Database, ProQuest Dissertations & Theses Global, Psychology Database, PTSDpubs, Public Health Database, Social Science Database, Sociological Abstracts, Sociology Database |
| S3 | ab(case-control OR "case control" OR correlation OR coefficient* OR cohort* OR covariat* OR cross-section* OR "cross section*" OR empirical OR longitudinal OR multivariate OR odds OR paramet* OR predict* OR prevalen* OR prospective OR questionnaire* OR quantitative OR rate* OR regression OR retrospective OR risk* OR survey* OR sampl* OR "standard deviation*" OR statistic* OR variable* OR variance) OR su(case-control OR "case control" OR correlation OR coefficient* OR cohort* OR covariat* OR cross-section* OR "cross section*" OR empirical OR longitudinal OR multivariate OR odds OR paramet* OR predict* OR prevalen* OR prospective OR questionnaire* OR quantitative OR rate* OR regression OR retrospective OR risk* OR survey* OR sampl* OR "standard deviation*" OR statistic* OR variable* OR variance) | Criminal Justice Database, International Bibliography of the Social Sciences (IBSS), Political Science Database, ProQuest Dissertations & Theses Global, Psychology Database, PTSDpubs, Public Health Database, Social Science Database, Sociological Abstracts, Sociology Database |
| S4 | S1 AND S2 AND S3 | Criminal Justice Database, International Bibliography of the Social Sciences (IBSS), Political Science Database, ProQuest Dissertations & Theses Global, Psychology Database, PTSDpubs, Public Health Database, Social Science Database, Sociological Abstracts, Sociology Database  These databases are searched for part of your query. |
| S18 | (S1 AND S2 AND S3) NOT stype.exact("Newspapers" OR "Blogs, Podcasts, & Websites" OR "Wire Feeds") | Criminal Justice Database, International Bibliography of the Social Sciences (IBSS), Political Science Database, ProQuest Dissertations & Theses Global, Psychology Database, PTSDpubs, Public Health Database, Social Science Database, Sociological Abstracts, Sociology Database  These databases are searched for part of your query. |
| S19 | (S1 AND S2 AND S3) NOT (at.exact("News" OR "General Information" OR "Correspondence" OR "Interview" OR "Letter to the Editor" OR "Transcript" OR "Back Matter" OR "Fiction" OR "Market Research" OR "Obituary" OR "Recipe" OR "Advertisement" OR "Biography" OR "Blog" OR "Credit/Acknowledgement" OR "Prose") NOT stype.exact("Newspapers" OR "Blogs, Podcasts, & Websites" OR "Wire Feeds")) | Criminal Justice Database, International Bibliography of the Social Sciences (IBSS), Political Science Database, ProQuest Dissertations & Theses Global, Psychology Database, PTSDpubs, Public Health Database, Social Science Database, Sociological Abstracts, Sociology Database  These databases are searched for part of your query. |
| S20 | (S1 AND S2 AND S3) NOT (bdl(1008752 1008751 1008748 1008747) NOT at.exact("News" OR "General Information" OR "Correspondence" OR "Interview" OR "Letter to the Editor" OR "Transcript" OR "Back Matter" OR "Fiction" OR "Market Research" OR "Obituary" OR "Recipe" OR "Advertisement" OR "Biography" OR "Blog" OR "Credit/Acknowledgement" OR "Prose") NOT stype.exact("Newspapers" OR "Blogs, Podcasts, & Websites" OR "Wire Feeds")) | Criminal Justice Database, International Bibliography of the Social Sciences (IBSS), Political Science Database, ProQuest Dissertations & Theses Global, Psychology Database, PTSDpubs, Public Health Database, Social Science Database, Sociological Abstracts, Sociology Database  These databases are searched for part of your query. |

### *Ovid (PsychINFO, PsycEXTRA, PsycArticles)*

| APA PsycArticles Full Text | |
| --- | --- |
| APA PsycExtra <1908 to March 14, 2022> | |
| APA PsycInfo <1806 to March Week 4 2022> | |
|  | |
| 1 | (worr* or afraid or fear* or unhapp* or sad* or emotion* or quiet* or withdraw* or guilt* or worthless* or suicid* or harm* or mood* or affect* or addict* or neuro* or intellect* or autis* or ASD or ADHD or motor or schizo* or bipolar or bi-polar or phobi* or panic or OCD or obsess* or trauma* or PTSD or attach* or dissociat* or somatic or eating or anorexi* or sleep* or insomni* or compulsi* or dysphori* or oppositional or conduct or antisocial or stress or stressor* or stressful or anxiet* or anxious* or depressed or depression or depressive or alcoholi*).ab,hw,id,mh,ot,ti. |
| 2 | (psych* adj3 (disorder* or symptom* or illness* or "ill")).ab,hw,id,mh,ot,ti. |
| 3 | (mental* adj3 (disorder* or symptom* or illness* or "ill")).ab,hw,id,mh,ot,ti. |
| 4 | (learning adj3 (disorder* or impair*)).ab,hw,id,mh,ot,ti. |
| 5 | (alcohol adj3 (disorder* or misuse or "use" or user or users)).ab,hw,id,mh,ot,ti. |
| 6 | (drug* adj3 (disorder* or misuse or "use" or user or users)).ab,hw,id,mh,ot,ti. |
| 7 | (substance* adj3 (disorder* or misuse or "use" or user or users)).ab,hw,id,mh,ot,ti. |
| 8 | (attention* adj5 (disorder* or symptom* or impair*)).ab,hw,id,mh,ot,ti. |
| 9 | (cognitive* adj3 (disorder* or impair*)).ab,hw,id,mh,ot,ti. |
| 10 | (communication* adj3 (disorder* or symptom* or impair*)).ab,hw,id,mh,ot,ti. |
| 11 | (personality* adj3 (disorder* or symptom*)).ab,hw,id,mh,ot,ti. |
| 12 | 1 or 2 or 3 or 4 or 5 or 6 or 7 or 8 or 9 or 10 or 11 |
| 13 | (terror* or extremism or extremist* or "radical" or radicals or radicali* or "political violence" or "lone actor*" or lone-actor* or Jihadi or Salafi or "right wing" or right-wing or separatist* or nationalist* or religio* or guerrilla* or paramilitary).ab,hw,id,mh,ot,ti. |
| 14 | (case-control or "case control" or correlation or coefficient* or cohort* or covariat* or cross-section* or "cross section*" or empirical or longitudinal or multivariate or odds or paramet* or predict* or prevalen* or prospective or questionnaire* or quantitative or rate* or regression or retrospective or risk* or survey* or sampl* or "standard deviation*" or statistic* or variable* or variance).ab,hw,id,mh,ot,ti. |
| 15 | 12 and 13 and 14 |

### *Embase*

| No. | Query |
| --- | --- |
| #20 | #19 AND ('abnormal behavior'/dm OR 'abnormal dreaming'/dm OR 'abnormal thinking'/dm OR 'absence'/dm OR 'acquired brain injury'/dm OR 'acrophobia'/dm OR 'acute disease'/dm OR 'acute liver failure'/dm OR 'acute psychosis'/dm OR 'acute stress disorder'/dm OR 'acute toxicity'/dm OR 'addiction'/dm OR 'adjustment disorder'/dm OR 'adolescent depression'/dm OR 'adolescent disease'/dm OR 'adolescent obesity'/dm OR 'adult disease'/dm OR 'adverse drug reaction'/dm OR 'adverse event'/dm OR 'affective neurosis'/dm OR 'affective psychosis'/dm OR 'agnosia'/dm OR 'agoraphobia'/dm OR 'akathisia'/dm OR 'akinesia'/dm OR 'alcohol intoxication'/dm OR 'alcohol liver cirrhosis'/dm OR 'alcohol liver disease'/dm OR 'alcohol psychosis'/dm OR 'alcohol withdrawal syndrome'/dm OR 'alcoholic cardiomyopathy'/dm OR 'alcoholic fatty liver'/dm OR 'alcoholic pancreatitis'/dm OR 'alcoholism'/dm OR 'alexia without agraphia'/dm OR 'alexithymia'/dm OR 'altered state of consciousness'/dm OR 'amphetamine dependence'/dm OR 'anorexia'/dm OR 'anorexia nervosa'/dm OR 'anosognosia'/dm OR 'antenatal depression'/dm OR 'anxiety disorder'/dm OR 'anxiety neurosis'/dm OR 'aphasia'/dm OR 'appetite disorder'/dm OR 'apraxia'/dm OR 'apraxia of speech'/dm OR 'asperger syndrome'/dm OR 'ataxia'/dm OR 'ataxic aphasia'/dm OR 'attention deficit disorder'/dm OR 'attention disturbance'/dm OR 'auditory hallucination'/dm OR 'autism'/dm OR 'avoidant personality disorder'/dm OR 'avoidant restrictive food intake disorder'/dm OR 'balance disorder'/dm OR 'balance impairment'/dm OR 'battered child syndrome'/dm OR 'behavior disorder'/dm OR 'behavioral addiction'/dm OR 'benzodiazepine dependence'/dm OR 'binge eating disorder'/dm OR 'bipolar depression'/dm OR 'bipolar disorder'/dm OR 'bipolar i disorder'/dm OR 'bipolar ii disorder'/dm OR 'blood phobia'/dm OR 'blood toxicity'/dm OR 'body dysmorphic disorder'/dm OR 'body weight disorder'/dm OR 'borderline state'/dm OR 'brain abscess'/dm OR 'brain arteriovenous malformation'/dm OR 'brain atrophy'/dm OR 'brain cortex atrophy'/dm OR 'brain damage'/dm OR 'brain degeneration'/dm OR 'brain disease'/dm OR 'brain dysfunction'/dm OR 'brain edema'/dm OR 'brain hemorrhage'/dm OR 'brain injury'/dm OR 'brain ischemia'/dm OR 'brain malformation'/dm OR 'brain necrosis'/dm OR 'brain toxicity'/dm OR 'brief psychotic disorder'/dm OR 'bulimia'/dm OR 'cannabis addiction'/dm OR 'castration anxiety'/dm OR 'catatonia'/dm OR 'chest tightness'/dm OR 'childhood trauma'/dm OR 'chromosome deletion'/dm OR 'chromosome disorder'/dm OR 'chronic depression'/dm OR 'chronic disease'/dm OR 'chronic fatigue syndrome'/dm OR 'chronic liver disease'/dm OR 'chronic liver failure'/dm OR 'chronic pain'/dm OR 'claustrophobia'/dm OR 'cocaine dependence'/dm OR 'cognitive defect'/dm OR 'combat stress'/dm OR 'communication disorder'/dm OR 'complicated grief'/dm OR 'complication'/dm OR 'compulsion'/dm OR 'compulsive buying'/dm OR 'compulsive personality disorder'/dm OR 'concentration loss'/dm OR 'conduct disorder'/dm OR 'confusion'/dm OR 'congenital disorder'/dm OR 'consciousness disorder'/dm OR 'conversion disorder'/dm OR 'cumulative trauma disorder'/dm OR 'decompensated liver cirrhosis'/dm OR 'decreased appetite'/dm OR 'defecation disorder'/dm OR 'delayed sleep phase syndrome'/dm OR 'delirium'/dm OR 'delirium tremens'/dm OR 'delusion'/dm OR 'delusional disorder'/dm OR 'delusional parasitosis'/dm OR 'delusional pregnancy'/dm OR 'dependent personality disorder'/dm OR 'depersonalization'/dm OR 'depression'/dm OR 'developmental coordination disorder'/dm OR 'developmental disorder'/dm OR 'developmental toxicity'/dm OR 'diarrhea'/dm OR 'diffuse brain injury'/dm OR 'disability'/dm OR 'disease exacerbation'/dm OR 'diseases'/dm OR 'disorder of sex development'/dm OR 'disorientation'/dm OR 'disruptive behavior'/dm OR 'dissociative fugue'/dm OR 'distress syndrome'/dm OR 'diurnal enuresis'/dm OR 'down syndrome'/dm OR 'drowsiness'/dm OR 'drug abuse pattern'/dm OR 'drug craving'/dm OR 'drug dependence'/dm OR 'drug fatality'/dm OR 'drug hypersensitivity'/dm OR 'drug induced disease'/dm OR 'drug intoxication'/dm OR 'drug misuse'/dm OR 'drug overdose'/dm OR 'drug seeking behavior'/dm OR 'drug toxicity'/dm OR 'dyskinesia'/dm OR 'dyslexia'/dm OR 'eating disorder'/dm OR 'emotional disorder'/dm OR 'enuresis'/dm OR 'erotomania'/dm OR 'exhaustion'/dm OR 'factitious disease'/dm OR 'faintness'/dm OR 'familial disease'/dm OR 'fatal familial insomnia'/dm OR 'fatigue'/dm OR 'feeding difficulty'/dm OR 'feeding disorder'/dm OR 'fetal alcohol syndrome'/dm OR 'fetal malnutrition'/dm OR 'fibromyalgia'/dm OR 'flying phobia'/dm OR 'food aversion'/dm OR 'food refusal'/dm OR 'fragile x syndrome'/dm OR 'game addiction'/dm OR 'gender dysphoria'/dm OR 'generalized anxiety disorder'/dm OR 'genetic disorder'/dm OR 'glasgow coma scale'/dm OR 'glue sniffing'/dm OR 'grandiose delusion'/dm OR 'gunshot injury'/dm OR 'hallucination'/dm OR 'hangover'/dm OR 'happy puppet syndrome'/dm OR 'head injury'/dm OR 'headache'/dm OR 'heroin dependence'/dm OR 'historical trauma'/dm OR 'hoarding disorder'/dm OR 'hyperactivity'/dm OR 'hypersensitivity'/dm OR 'hypersexuality'/dm OR 'hypersomnia'/dm OR 'hyperventilation'/dm OR 'hypoactivity'/dm OR 'hypochondriasis'/dm OR 'hypomania'/dm OR 'hysteria'/dm OR 'impulse control disorder'/dm OR 'increased appetite'/dm OR 'injury'/dm OR 'injury severity'/dm OR 'insomnia'/dm OR 'internalizing disorder'/dm OR 'intoxication'/dm OR 'intractable pain'/dm OR 'jealous delusion'/dm OR 'language delay'/dm OR 'language disability'/dm OR 'late life depression'/dm OR 'learning disorder'/dm OR 'lethargy'/dm OR 'listlessness'/dm OR 'liver cell damage'/dm OR 'liver cirrhosis'/dm OR 'liver disease'/dm OR 'liver dysfunction'/dm OR 'liver failure'/dm OR 'liver necrosis'/dm OR 'liver toxicity'/dm OR 'loss of appetite'/dm OR 'major affective disorder'/dm OR 'major depression'/dm OR 'malaise'/dm OR 'mania'/dm OR 'melancholia'/dm OR 'memory disorder'/dm OR 'mental deficiency'/dm OR 'mental deterioration'/dm OR 'mental disease'/dm OR 'mental instability'/dm OR 'microcephaly'/dm OR 'mild cognitive impairment'/dm OR 'minimal brain dysfunction'/dm OR 'minimal residual disease'/dm OR 'mixed mania and depression'/dm OR 'mood disorder'/dm OR 'morbid obesity'/dm OR 'morphine addiction'/dm OR 'motor dysfunction'/dm OR 'multiple chronic conditions'/dm OR 'multiple drug abuse'/dm OR 'multiple personality'/dm OR 'multiple trauma'/dm OR 'mutism'/dm OR 'myopia'/dm OR 'narcissism'/dm OR 'narcotic dependence'/dm OR 'nausea'/dm OR 'near-death experience'/dm OR 'needle phobia'/dm OR 'negative syndrome'/dm OR 'neural tube defect'/dm OR 'neurodisability'/dm OR 'neurologic disease'/dm OR 'neuropathy'/dm OR 'neurosis'/dm OR 'neurotoxicity'/dm OR 'night sweat'/dm OR 'nightmare'/dm OR 'nocturnal enuresis'/dm OR 'nutritional disorder'/dm OR 'obsession'/dm OR 'obsessive compulsive disorder'/dm OR 'obsessive hoarding'/dm OR 'opiate addiction'/dm OR 'oppositional defiant disorder'/dm OR 'organic brain syndrome'/dm OR 'overnutrition'/dm OR 'pain'/dm OR 'panic'/dm OR 'paranoia'/dm OR 'paranoid psychosis'/dm OR 'paranoid schizophrenia'/dm OR 'paraphilic disorder'/dm OR 'parasomnia'/dm OR 'perception disorder'/dm OR 'perinatal depression'/dm OR 'persian gulf syndrome'/dm OR 'personality disorder'/dm OR 'phobia'/dm OR 'physical disability'/dm OR 'pica'/dm OR 'positive syndrome'/dm OR 'post-stroke depression'/dm OR 'postnatal depression'/dm OR 'postoperative cognitive dysfunction'/dm OR 'postoperative delirium'/dm OR 'postoperative depression'/dm OR 'posttraumatic complication'/dm OR 'posttraumatic stress disorder'/dm OR 'premenstrual dysphoric disorder'/dm OR 'prescription drug misuse'/dm OR 'primary insomnia'/dm OR 'problem behavior'/dm OR 'prosopagnosia'/dm OR 'pseudotumor'/dm OR 'psychiatric complication'/dm OR 'psychogenic pain'/dm OR 'psychomotor disorder'/dm OR 'psychomotor retardation'/dm OR 'psychopathy'/dm OR 'psychosexual disorder'/dm OR 'psychosis'/dm OR 'psychosocial disorder'/dm OR 'psychosomatic disorder'/dm OR 'psychotrauma'/dm OR 'puerperal psychosis'/dm OR 'rare disease'/dm OR 'recurrent disease'/dm OR 'relapse'/dm OR 'restless legs syndrome'/dm OR 'restlessness'/dm OR 'schizoaffective psychosis'/dm OR 'schizoidism'/dm OR 'schizophrenia'/dm OR 'schizophrenia spectrum disorder'/dm OR 'schizophreniform disorder'/dm OR 'seasonal affective disorder'/dm OR 'seizure'/dm OR 'separation anxiety'/dm OR 'sexual addiction'/dm OR 'sexual trauma'/dm OR 'shared psychotic disorder'/dm OR 'shock'/dm OR 'side effect'/dm OR 'sleep arousal disorder'/dm OR 'sleep disorder'/dm OR 'sleep disordered breathing'/dm OR 'sleep walking'/dm OR 'snoring'/dm OR 'social phobia'/dm OR 'somatization'/dm OR 'somatoform disorder'/dm OR 'speech delay'/dm OR 'speech disorder'/dm OR 'suicidal behavior'/dm OR 'suicidal ideation'/dm OR 'suicide'/dm OR 'suicide attempt'/dm OR 'syndrome'/dm OR 'tardive dyskinesia'/dm OR 'tension headache'/dm OR 'thought disorder'/dm OR 'tic'/dm OR 'tobacco dependence'/dm OR 'transsexualism'/dm OR 'traumatic brain injury'/dm OR 'traumatic shock'/dm OR 'treatment resistant depression'/dm OR 'trisomy'/dm OR 'trisomy 13'/dm OR 'trisomy 18'/dm OR 'trisomy 21'/dm OR 'unconsciousness'/dm OR 'underweight'/dm OR 'unspecified side effect'/dm OR 'visual hallucination'/dm OR 'vivid dream'/dm OR 'vomiting'/dm OR 'wernicke encephalopathy'/dm OR 'withdrawal syndrome'/dm OR 'work disability'/dm OR 'x chromosome linked disorder'/dm OR 'catalepsy'/dm OR 'knife cut'/dm OR 'mental retardation malformation syndrome'/dm OR 'minor depression'/dm OR 'mobile phone addiction'/dm OR 'munchausen syndrome'/dm OR 'munchausen syndrome by proxy'/dm OR 'necrophilia'/dm OR 'nymphomania'/dm OR 'paranoid personality disorder'/dm OR 'pediatric traumatic brain injury'/dm OR 'pervasive developmental disorder not otherwise specified'/dm OR 'progressive nonfluent aphasia'/dm OR 'pseudodementia'/dm OR 'rumination syndrome'/dm OR 'selective mutism'/dm OR 'slurred speech'/dm OR 'speech sound disorder'/dm OR 'stomach discomfort'/dm OR 'syndrome x'/dm OR 'trisomy 8'/dm OR 'wernicke korsakoff syndrome'/dm) |
| #19 | #13 AND #16 AND #17 AND [humans]/lim AND [embase]/lim |
| #18 | #13 AND #16 AND #17 |
| #17 | 'case control':ti,ab,kw OR correlation:ti,ab,kw OR coefficient*:ti,ab,kw OR cohort*:ti,ab,kw OR covariat*:ti,ab,kw OR 'cross section*':ti,ab,kw OR empirical:ti,ab,kw OR longitudinal:ti,ab,kw OR multivariate:ti,ab,kw OR odds:ti,ab,kw OR paramet*:ti,ab,kw OR predict*:ti,ab,kw OR prevalen*:ti,ab,kw OR prospective:ti,ab,kw OR questionnaire*:ti,ab,kw OR quantitative:ti,ab,kw OR rate*:ti,ab,kw OR regression:ti,ab,kw OR retrospective:ti,ab,kw OR risk*:ti,ab,kw OR survey*:ti,ab,kw OR sampl*:ti,ab,kw OR 'standard deviation*':ti,ab,kw OR statistic*:ti,ab,kw OR variable*:ti,ab,kw OR variance:ti,ab,kw |
| #16 | #14 OR #15 |
| #15 | 'terrorism'/exp |
| #14 | terror*:ti,ab,kw OR extremism:ti,ab,kw OR extremist*:ti,ab,kw OR 'radical':ti,ab,kw OR radicals:ti,ab,kw OR radicali*:ti,ab,kw OR 'political violence':ti,ab,kw OR 'lone actor*':ti,ab,kw OR jihadi:ti,ab,kw OR salafi:ti,ab,kw OR 'right wing':ti,ab,kw OR separatist*:ti,ab,kw OR nationalist*:ti,ab,kw OR religio*:ti,ab,kw OR guerrilla*:ti,ab,kw OR paramilitary:ti,ab,kw |
| #13 | #1 OR #2 OR #3 OR #4 OR #5 OR #6 OR #7 OR #8 OR #9 OR #10 OR #11 OR #12 |
| #12 | 'mental disease'/exp |
| #11 | (substance* NEAR/3 (disorder* OR misuse OR 'use' OR user OR users)):ti,ab,kw |
| #10 | (drug* NEAR/3 (disorder* OR misuse OR 'use' OR user OR users)):ti,ab,kw |
| #9 | (alcohol NEAR/3 (disorder* OR misuse OR 'use' OR user OR users)):ti,ab,kw |
| #8 | (personality* NEAR/3 (disorder* OR symptom*)):ti,ab,kw |
| #7 | (cognitive* NEAR/3 (disorder* OR impair*)):ti,ab,kw |
| #6 | (communication* NEAR/3 (disorder* OR symptom* OR impair*)):ti,ab,kw |
| #5 | (attention* NEAR/5 (disorder* OR symptom* OR impair*)):ti,ab,kw |
| #4 | (learning NEAR/3 (disorder* OR impair*)):ti,ab,kw |
| #3 | (mental* NEAR/3 (disorder* OR symptom* OR illness* OR 'ill')):ti,ab,kw |
| #2 | (psych* NEAR/3 (disorder* OR symptom* OR illness* OR 'ill')):ti,ab,kw |
| #1 | worr*:ti,ab,kw OR afraid:ti,ab,kw OR fear*:ti,ab,kw OR unhapp*:ti,ab,kw OR sad*:ti,ab,kw OR emotion*:ti,ab,kw OR quiet*:ti,ab,kw OR withdraw*:ti,ab,kw OR guilt*:ti,ab,kw OR worthless*:ti,ab,kw OR suicid*:ti,ab,kw OR harm*:ti,ab,kw OR mood*:ti,ab,kw OR affect*:ti,ab,kw OR addict*:ti,ab,kw OR neuro*:ti,ab,kw OR intellect*:ti,ab,kw OR autis*:ti,ab,kw OR asd:ti,ab,kw OR adhd:ti,ab,kw OR motor:ti,ab,kw OR schizo*:ti,ab,kw OR bipolar:ti,ab,kw OR 'bi polar':ti,ab,kw OR phobi*:ti,ab,kw OR panic:ti,ab,kw OR ocd:ti,ab,kw OR obsess*:ti,ab,kw OR trauma*:ti,ab,kw OR ptsd:ti,ab,kw OR attach*:ti,ab,kw OR dissociat*:ti,ab,kw OR somatic:ti,ab,kw OR eating:ti,ab,kw OR anorexi*:ti,ab,kw OR sleep*:ti,ab,kw OR insomni*:ti,ab,kw OR compulsi*:ti,ab,kw OR dysphori*:ti,ab,kw OR oppositional:ti,ab,kw OR conduct:ti,ab,kw OR antisocial:ti,ab,kw OR stress:ti,ab,kw OR stressor*:ti,ab,kw OR stressful:ti,ab,kw OR anxiet*:ti,ab,kw OR anxious*:ti,ab,kw OR depressed:ti,ab,kw OR depression:ti,ab,kw OR depressive:ti,ab,kw OR alcoholi*:ti,ab,kw |

### *Web of Science*

| **#14 AND #13 AND #12** and **Book Reviews** or **Notes** or **Letters** or **Biographical-Items** or **Reprints** or **Chronologies** or **Editorial Materials** (Exclude – Document Types) and **Oncology** or **Business Finance** or **Plant Sciences** or **Architecture** or **Energy Fuels** or **Orthopedics** or **Engineering Environmental** or **Agriculture Multidisciplinary** or **Veterinary Sciences** or **Gastroenterology Hepatology** or **Dermatology** or **Agricultural Economics Policy** or **Transplantation** or **Cardiac Cardiovascular Systems** or **Engineering Civil** or **Engineering Multidisciplinary** or **Meteorology Atmospheric Sciences** or **Respiratory System** or **Engineering Industrial** or **Food Science Technology** or **Otorhinolaryngology** or **Peripheral Vascular Disease** or **Biodiversity Conservation** or **Materials Science Multidisciplinary** or **Construction Building Technology** or **Rheumatology** or **Agriculture Dairy Animal Science** or **Engineering Electrical Electronic** or **Zoology** or **Engineering Chemical** or **Engineering Manufacturing** or **Forestry** or **Allergy** or **Geography Physical** or **Medieval Renaissance Studies** or **Materials Science Biomaterials** or **Engineering Geological** or **Geology** or **Marine Freshwater Biology** or **Polymer Science** or **Engineering Aerospace** or **Engineering Mechanical** or **Geochemistry Geophysics** or **Literature Romance** or **Mining Mineral Processing** or **Physics Condensed Matter** (Exclude – Web of Science Categories) | | | |
| --- | --- | --- | --- |
| 5:51 PM \| Exact search | |  |  |
| Web of Science Core CollectionHide editions | | | |
| Editions = BKCI-SSH , ESCI , CPCI-SSH , SSCI | | | |
|  |  |  |  |
| **#14 AND #13 AND #12** and **Book Reviews** or **Notes** or **Letters** or **Biographical-Items** or **Reprints** or **Chronologies** or **Editorial Materials** (Exclude – Document Types) | | | |
| 5:48 PM \| Exact search | |  |  |
| Web of Science Core CollectionHide editions | | | |
| Editions = BKCI-SSH , ESCI , CPCI-SSH , SSCI | | | |
|  |  |  |  |
| **#14 AND #13 AND #12** | | |  |
| 5:47 PM \| Exact search | |  |  |
| Web of Science Core CollectionHide editions | | | |
| Editions = BKCI-SSH , ESCI , CPCI-SSH , SSCI | | | |
|  |  |  |  |
| **TS=(case-control or "case control" or correlation or coefficient* or cohort* or covariat* or cross-section* or "cross section*" or empirical or longitudinal or multivariate or odds or paramet* or predict* or prevalen* or prospective or questionnaire* or quantitative or rate* or regression or retrospective or risk* or survey* or sampl* or "standard deviation*" or statistic* or variable* or variance)** | | | |
| 5:46 PM \| Exact search | |  |  |
| Web of Science Core CollectionHide editions | | | |
| Editions = BKCI-SSH , ESCI , CPCI-SSH , SSCI | | | |
|  |  |  |  |
| **TS=(terror* OR extremism OR extremist* OR "radical" OR radicals OR radicali* OR "political violence" or "lone actor*" or lone-actor* or Jihadi or Salafi or "right wing" or right-wing or separatist* or nationalist* or religio* or guerrilla* or paramilitary)** | | | |
| 5:46 PM \| Exact search | |  |  |
| Web of Science Core CollectionHide editions | | | |
| Editions = BKCI-SSH , ESCI , CPCI-SSH , SSCI | | | |
|  |  |  |  |
| **#11 OR #10 OR #9 OR #8 OR #7 OR #6 OR #5 OR #4 OR #3 OR #2 OR #1** | | | |
| 5:46 PM \| Exact search | |  |  |
| Web of Science Core CollectionHide editions | | | |
| Editions = BKCI-SSH , ESCI , CPCI-SSH , SSCI | | | |
|  |  |  |  |
| **TS=(personality* NEAR/3 (disorder* OR symptom*))** | | | |
| 5:45 PM \| Exact search | |  |  |
| Web of Science Core CollectionHide editions | | | |
| Editions = BKCI-SSH , ESCI , CPCI-SSH , SSCI | | | |
|  |  |  |  |
| **TS=(cognitive* NEAR/3 (disorder* OR impair*))** | | | |
| 5:45 PM \| Exact search | |  |  |
| Web of Science Core CollectionHide editions | | | |
| Editions = BKCI-SSH , ESCI , CPCI-SSH , SSCI | | | |
|  |  |  |  |
| **TS=(communication* NEAR/3 (disorder* OR symptom* OR impair*))** | | | |
| 5:45 PM \| Exact search | |  |  |
| Web of Science Core CollectionHide editions | | | |
| Editions = BKCI-SSH , ESCI , CPCI-SSH , SSCI | | | |
|  |  |  |  |
| **TS=(attention* NEAR/5 (disorder* OR symptom* OR impair*))** | | | |
| 5:45 PM \| Exact search | |  |  |
| Web of Science Core CollectionHide editions | | | |
| Editions = BKCI-SSH , ESCI , CPCI-SSH , SSCI | | | |
|  |  |  |  |
| **TS=(substance* NEAR/3 (disorder* OR misuse OR "use" OR user OR users))** | | | |
| 5:44 PM \| Exact search | |  |  |
| Web of Science Core CollectionHide editions | | | |
| Editions = BKCI-SSH , ESCI , CPCI-SSH , SSCI | | | |
|  |  |  |  |
| **TS=(drug* NEAR/3 (disorder* OR misuse OR "use" OR user OR users))** | | | |
| 5:44 PM \| Exact search | |  |  |
| Web of Science Core CollectionHide editions | | | |
| Editions = BKCI-SSH , ESCI , CPCI-SSH , SSCI | | | |
|  |  |  |  |
| **TS=(alcohol NEAR/3 (disorder* OR misuse OR "use" OR user OR users))** | | | |
| 5:44 PM \| Exact search | |  |  |
| Web of Science Core CollectionHide editions | | | |
| Editions = BKCI-SSH , ESCI , CPCI-SSH , SSCI | | | |
|  |  |  |  |
| **TS=(learning NEAR/3 (disorder* OR impair*))** | | | |
| 5:43 PM \| Exact search | |  |  |
| Web of Science Core CollectionHide editions | | | |
| Editions = BKCI-SSH , ESCI , CPCI-SSH , SSCI | | | |
|  |  |  |  |
| **TS=(mental* NEAR/3 (disorder* OR symptom* OR illness* OR "ill"))** | | | |
| 5:43 PM \| Exact search | |  |  |
| Web of Science Core CollectionHide editions | | | |
| Editions = BKCI-SSH , ESCI , CPCI-SSH , SSCI | | | |
|  |  |  |  |
| **TS=(psych* NEAR/3 (disorder* OR symptom* OR illness* OR "ill"))** | | | |
| 5:43 PM \| Exact search | |  |  |
| Web of Science Core CollectionHide editions | | | |
| Editions = BKCI-SSH , ESCI , CPCI-SSH , SSCI | | | |
|  |  |  |  |
| **TS=(worr* OR afraid OR fear* OR unhapp* OR "sad" OR sadness OR emotion* OR quiet* OR withdraw* OR guilt* OR worthless* OR suicid* OR harm* OR mood* OR affect* OR addict* OR neuro* OR intellect* OR autis* OR "ASD" OR ADHD OR motor OR schizo* OR bipolar OR bi-polar OR phobi* OR panic OR OCD OR obsess* OR trauma* OR PTSD OR attach* OR dissociat* OR somatic OR eating OR anorexi* OR sleep* OR insomni* OR compulsi* OR dysphori* OR oppositional OR conduct OR antisocial OR anxious* OR anxiet* OR alcoholi* OR depressed OR depression OR depressive OR stress OR stressor* OR stressful)** | | | |
| 5:42 PM \| Exact search | |  |  |
| Web of Science Core CollectionHide editions | | | |
| Editions = BKCI-SSH , ESCI , CPCI-SSH , SSCI | | | |

### *Scopus*

( TITLE-ABS-KEY ( case-control OR "case control" ORcorrelation OR coefficient* OR cohort* OR covariat*OR cross-section* OR "cross section*" OR empiricalOR longitudinal OR multivariate OR odds OR paramet* OR predict* OR prevalen* OR prospective ORquestionnaire* OR quantitative OR rate* OR regression OR retrospective OR risk* OR survey* OR sampl* OR "standard deviation*" OR statistic* OR variable* OR variance ) ) AND ( TITLE-ABS-KEY ( terror* ORextremism OR extremist* OR "radical" OR radicalsOR radicali* OR "political violence" OR "lone actor*"OR lone-actor* OR jihadi OR salafi OR "right wing"OR right-wing OR separatist* OR nationalist* OR religio* OR guerrilla* OR paramilitary ) ) AND ( ( TITLE-ABS-KEY ( worr* OR afraid OR fear* OR unhapp*OR "sad" OR sadness OR emotion* OR quiet* ORwithdraw* OR guilt* OR worthless* OR suicid* ORharm* OR mood* OR affect* OR addict* OR neuro* OR intellect* OR autis* OR "ASD" OR adhd ORmotor OR schizo* OR bipolar OR bi-polar OR phobi* OR panic OR ocd OR obsess* OR trauma* OR ptsd OR attach* OR dissociat* OR somatic OR eating OR anorexi* OR sleep* OR insomni* OR compulsi* OR dysphori* OR oppositional OR conduct ORantisocial OR anxious* OR anxiet* OR alcoholi* ORdepressed OR depression OR depressive OR stressOR stressor* OR stressful ) OR TITLE-ABS-KEY ( psych* W/3 ( disorder* OR symptom* OR illness* OR "ill" ) ) OR TITLE-ABS-KEY ( mental* W/3 ( disorder* OR symptom* OR illness* OR "ill" ) ) OR TITLE-ABSKEY( learning W/3 ( disorder* OR impair* ) ) OR TITLE-ABS-KEY ( alcohol W/3 ( disorder* OR misuse OR"use" OR user OR users ) ) OR TITLE-ABS-KEY ( drug* W/3 ( disorder* OR misuse OR "use" OR user ORusers ) ) OR TITLE-ABS-KEY ( substance* W/3 ( disorder* OR misuse OR "use" OR user OR users ) ) ORTITLE-ABS-KEY ( attention* W/5 ( disorder* OR symptom* OR impair* ) ) OR TITLE-ABS-KEY ( communication* W/3 ( disorder* OR symptom* OR impair* ) )OR TITLE-ABS-KEY ( cognitive* W/3 ( disorder* OR impair* ) ) OR TITLE-ABS-KEY ( personality* W/3 ( disorder* OR symptom* ) ) ) ) AND ( EXCLUDE ( DOCTYPE , "ed" ) OR EXCLUDE ( DOCTYPE , "no" ) OR EXCLUDE ( DOCTYPE , "le" ) ) AND ( EXCLUDE ( EXACTKEYWORD , "Nonhuman" ) OR EXCLUDE ( EXACTKEYWORD , "Animals" ) OR EXCLUDE ( EXACTKEYWORD ,"Animal Experiment" ) OR EXCLUDE ( EXACTKEYWORD , "Rat" ) OR EXCLUDE ( EXACTKEYWORD , "Animal" ) OR EXCLUDE ( EXACTKEYWORD , "Animal Tissue") OR EXCLUDE ( EXACTKEYWORD , "Animal Model" )OR EXCLUDE ( EXACTKEYWORD , "Rats" ) OR EXCLUDE ( EXACTKEYWORD , "Animal Cell" ) OR EXCLUDE (EXACTKEYWORD , "Mouse" ) OR EXCLUDE ( EXACTKEYWORD , "Cancer Surgery" ) OR EXCLUDE ( EXACTKEYWORD , "Prostate Cancer" ) OR EXCLUDE ( EXACTKEYWORD , "Mice" ) OR EXCLUDE ( EXACTKEYWORD, "Plant Extract" ) OR EXCLUDE ( EXACTKEYWORD ,"Cancer Radiotherapy" ) OR EXCLUDE ( EXACTKEYWORD , "Animalia" ) OR EXCLUDE ( EXACTKEYWORD ,"Rats, Wistar" ) OR EXCLUDE ( EXACTKEYWORD , "Iron" ) ) AND ( EXCLUDE ( SUBJAREA , "ENGI" ) OR EXCLUDE ( SUBJAREA , "PHYS" ) OR EXCLUDE ( SUBJAREA , "CENG" ) OR EXCLUDE ( SUBJAREA , "MATE" ) OR EXCLUDE ( SUBJAREA , "AGRI" ) OR EXCLUDE ( SUBJAREA , "ENVI" ) OR EXCLUDE ( SUBJAREA , "BUSI") OR EXCLUDE ( SUBJAREA , "ECON" ) OR EXCLUDE( SUBJAREA , "ENER" ) OR EXCLUDE ( SUBJAREA , "EART" ) OR EXCLUDE ( SUBJAREA , "DENT" ) OR EXCLUDE ( SUBJAREA , "VETE" ) ) AND ( EXCLUDE ( SUBJAREA , "BIOC" ) OR EXCLUDE ( SUBJAREA , "CHEM" )OR EXCLUDE ( SUBJAREA , "IMMU" ) ) AND ( EXCLUDE ( EXACTKEYWORD , "Prostatectomy" ) )

### *PubMed*

| Search number | Query |
| --- | --- |
| 21 | #13 AND #16 AND #17 |
| 19 | #13 AND #16 AND #17 |
| 17 | case-control[Title/Abstract] OR "case control"[Title/Abstract] OR correlation[Title/Abstract] OR coefficient*[Title/Abstract] OR cohort*[Title/Abstract] OR covariat*[Title/Abstract] OR cross-section*[Title/Abstract] OR "cross section*"[Title/Abstract] OR empirical[Title/Abstract] OR longitudinal[Title/Abstract] OR multivariate[Title/Abstract] OR odds[Title/Abstract] OR paramet*[Title/Abstract] OR predict*[Title/Abstract] OR prevalen*[Title/Abstract] OR prospective[Title/Abstract] OR questionnaire*[Title/Abstract] OR quantitative[Title/Abstract] OR rate*[Title/Abstract] OR regression[Title/Abstract] OR retrospective[Title/Abstract] OR risk*[Title/Abstract] OR survey*[Title/Abstract] OR sampl*[Title/Abstract] OR "standard deviation*"[Title/Abstract] OR statistic*[Title/Abstract] OR variable*[Title/Abstract] OR variance[Title/Abstract] |
| 16 | #14 OR #15 |
| 15 | terror*[Title/Abstract] OR extremist*[Title/Abstract] OR extremism[Title/Abstract] OR radicals[Title/Abstract] OR radicali*[Title/Abstract] OR "political violence"[Title/Abstract] OR "lone actor*"[Title/Abstract] OR jihadi[Title/Abstract] OR salafi IR "right wing"[Title/Abstract] OR separatist*[Title/Abstract] OR nationalist*[Title/Abstract] OR religio*[Title/Abstract] OR guerrilla*[Title/Abstract] OR paramilitary[Title/Abstract] |
| 14 | "terrorism"[MeSH Major Topic] |
| 13 | #12 OR #11 OR #10 OR #9 OR #8 OR #7 OR #6 OR #5 OR #4 OR #3 OR #2 OR #1 |
| 12 | (substance*[Title/Abstract]) AND (disorder*[Title/Abstract] OR misuse[Title/Abstract] OR "use"[Title/Abstract] OR "users"[Title/Abstract]) |
| 11 | (alcohol[Title/Abstract]) AND (disorder*[Title/Abstract] OR misuse[Title/Abstract] OR "use"[Title/Abstract] OR "users"[Title/Abstract]) |
| 10 | (drug*[Title/Abstract]) AND (disorder*[Title/Abstract] OR misuse[Title/Abstract] OR "use"[Title/Abstract] OR user[Title/Abstract] OR users[Title/Abstract]) |
| 9 | (learning[Title/Abstract]) AND (disorder*[Title/Abstract] OR impair*[Title/Abstract]) |
| 8 | (personality[Title/Abstract]) AND (disorder*[Title/Abstract] OR symptom*[Title/Abstract]) |
| 7 | (cognitive*[Title/Abstract]) AND (disorder*[Title/Abstract] OR impair*[Title/Abstract]) |
| 6 | (communication*[Title/Abstract]) AND (disorder*[Title/Abstract] OR symptom*[Title/Abstract] OR impair*[Title/Abstract]) |
| 5 | (attention*[Title/Abstract]) AND (disorder*[Title/Abstract] OR symptom*[Title/Abstract] OR impair*[Title/Abstract]) |
| 4 | (mental*[Title/Abstract]) AND (disorder*[Title/Abstract] OR symptom*[Title/Abstract] OR illness*[Title/Abstract] OR "ill"[Title/Abstract]) |
| 3 | (psych*[Title/Abstract]) AND (disorder*[Title/Abstract] OR symptom*[Title/Abstract] OR illness*[Title/Abstract] OR "ill"[Title/Abstract]) |
| 2 | worr*[Title/Abstract] OR afraid[Title/Abstract] OR fear*[Title/Abstract] OR unhapp*[Title/Abstract] OR sad[Title/Abstract] OR sadness[Title/Abstract] OR emotion*[Title/Abstract] OR quiet*[Title/Abstract] OR withdraw*[Title/Abstract] OR guilt*[Title/Abstract] OR worthless*[Title/Abstract] OR suicid*[Title/Abstract] OR harm*[Title/Abstract] OR mood*[Title/Abstract] OR affect*[Title/Abstract] OR addict*[Title/Abstract] OR neuro*[Title/Abstract] OR intellect*[Title/Abstract] OR autis*[Title/Abstract] OR ASD[Title/Abstract] OR ADHD[Title/Abstract] OR motor[Title/Abstract] OR schizo*[Title/Abstract] OR bipolar[Title/Abstract] OR bi-polar[Title/Abstract] OR phobi*[Title/Abstract] OR panic[Title/Abstract] OR OCD[Title/Abstract] OR obsess*[Title/Abstract] OR trauma*[Title/Abstract] OR PTSD[Title/Abstract] OR attach*[Title/Abstract] OR dissociat*[Title/Abstract] OR somatic[Title/Abstract] OR eating[Title/Abstract] OR anorexi*[Title/Abstract] OR sleep*[Title/Abstract] OR insomni*[Title/Abstract] OR compulsi*[Title/Abstract] OR dysphori*[Title/Abstract] OR oppositional[Title/Abstract] OR conduct[Title/Abstract] OR antisocial[Title/Abstract] OR stress[Title/Abstract] OR stressor*[Title/Abstract] OR stressful[Title/Abstract] OR depressed[Title/Abstract] OR depression[Title/Abstract] OR depressive[Title/Abstract] OR alcoholi*[Title/Abstract] OR anxious*[Title/Abstract] OR anxiet*[Title/Abstract] |
| 1 | "mental disorders"[MeSH Major Topic] |

### *Criminal Justice Abstracts*

| Search number | Query |
| --- | --- |
| 1 | TI (worr* or afraid or fear* or unhapp* or sad* or emotion* or quiet* or withdraw* or guilt* or worthless* or suicid* or harm* or mood* or affect* or addict* or neuro* or intellect* or autis* or ASD or ADHD or motor or schizo* or bipolar or bi-polar or phobi* or panic or OCD or obsess* or trauma* or PTSD or attach* or dissociat* or somatic or eating or anorexi* or sleep* or insomni* or compulsi* or dysphori* or oppositional or conduct or antisocial or stress or stressor* or stressful or anxiet* or anxious* or depressed or depression or depressive or alcoholi*) OR AB (worr* or afraid or fear* or unhapp* or sad* or emotion* or quiet* or withdraw* or guilt* or worthless* or suicid* or harm* or mood* or affect* or addict* or neuro* or intellect* or autis* or ASD or ADHD or motor or schizo* or bipolar or bi-polar or phobi* or panic or OCD or obsess* or trauma* or PTSD or attach* or dissociat* or somatic or eating or anorexi* or sleep* or insomni* or compulsi* or dysphori* or oppositional or conduct or antisocial or stress or stressor* or stressful or anxiet* or anxious* or depressed or depression or depressive or alcoholi*) OR KW (worr* or afraid or fear* or unhapp* or sad* or emotion* or quiet* or withdraw* or guilt* or worthless* or suicid* or harm* or mood* or affect* or addict* or neuro* or intellect* or autis* or ASD or ADHD or motor or schizo* or bipolar or bi-polar or phobi* or panic or OCD or obsess* or trauma* or PTSD or attach* or dissociat* or somatic or eating or anorexi* or sleep* or insomni* or compulsi* or dysphori* or oppositional or conduct or antisocial or stress or stressor* or stressful or anxiet* or anxious* or depressed or depression or depressive or alcoholi*) OR SU (worr* or afraid or fear* or unhapp* or sad* or emotion* or quiet* or withdraw* or guilt* or worthless* or suicid* or harm* or mood* or affect* or addict* or neuro* or intellect* or autis* or ASD or ADHD or motor or schizo* or bipolar or bi-polar or phobi* or panic or OCD or obsess* or trauma* or PTSD or attach* or dissociat* or somatic or eating or anorexi* or sleep* or insomni* or compulsi* or dysphori* or oppositional or conduct or antisocial or stress or stressor* or stressful or anxiet* or anxious* or depressed or depression or depressive or alcoholi*) |
| 2 | TI (psych* N3 (disorder* or symptom* or illness* or "ill")) OR AB (psych* N3 (disorder* or symptom* or illness* or "ill")) OR KW (psych* N3 (disorder* or symptom* or illness* or "ill")) OR SU (psych* N3 (disorder* or symptom* or illness* or "ill")) |
| 3 | TI (mental* N3 (disorder* or symptom* or illness* or "ill")) OR AB (mental* N3 (disorder* or symptom* or illness* or "ill")) OR KW (mental* N3 (disorder* or symptom* or illness* or "ill")) OR SU (mental* N3 (disorder* or symptom* or illness* or "ill")) |
| 4 | TI (learning N3 (disorder* or impair*)) OR AB (learning N3 (disorder* or impair*)) OR KW (learning N3 (disorder* or impair*)) OR SU (learning N3 (disorder* or impair*)) |
| 5 | TI (alcohol N3 (disorder* or misuse or "use" or user or users)) OR AB (alcohol N3 (disorder* or misuse or "use" or user or users)) OR KW (alcohol N3 (disorder* or misuse or "use" or user or users)) OR SU (alcohol N3 (disorder* or misuse or "use" or user or users)) |
| 6 | TI (drug* N3 (disorder* or misuse or "use" or user or users)) OR AB (drug* N3 (disorder* or misuse or "use" or user or users)) OR KW (drug* N3 (disorder* or misuse or "use" or user or users)) OR SU (drug* N3 (disorder* or misuse or "use" or user or users)) |
| 7 | TI (substance* N3 (disorder* or misuse or "use" or user or users)) OR AB (substance* N3 (disorder* or misuse or "use" or user or users)) OR KW (substance* N3 (disorder* or misuse or "use" or user or users)) OR SU (substance* N3 (disorder* or misuse or "use" or user or users)) |
| 8 | TI (attention* adj5 (disorder* or symptom* or impair*)) OR AB (attention* adj5 (disorder* or symptom* or impair*)) OR KW (attention* adj5 (disorder* or symptom* or impair*)) OR SU (attention* adj5 (disorder* or symptom* or impair*)) |
| 9 | TI (cognitive* N3 (disorder* or impair*)) OR AB (cognitive* N3 (disorder* or impair*)) OR KW (cognitive* N3 (disorder* or impair*)) OR SU (cognitive* N3 (disorder* or impair*)) |
| 10 | TI (communication* N3 (disorder* or symptom* or impair*)) OR AB (communication* N3 (disorder* or symptom* or impair*)) OR KW (communication* N3 (disorder* or symptom* or impair*)) OR SU (communication* N3 (disorder* or symptom* or impair*)) |
| 11 | TI (personality* N3 (disorder* or symptom*)) OR AB (personality* N3 (disorder* or symptom*)) OR KW (personality* N3 (disorder* or symptom*)) OR AB (personality* N3 (disorder* or symptom*)) |
| 12 | 1 or 2 or 3 or 4 or 5 or 6 or 7 or 8 or 9 or 10 or 11 |
| 13 | TI (terror* or extremism or extremist* or "radical" or radicals or radicali* or "political violence" or "lone actor*" or lone-actor* or Jihadi or Salafi or "right wing" or right-wing or separatist* or nationalist* or religio* or guerrilla* or paramilitary) OR AB (terror* or extremism or extremist* or "radical" or radicals or radicali* or "political violence" or "lone actor*" or lone-actor* or Jihadi or Salafi or "right wing" or right-wing or separatist* or nationalist* or religio* or guerrilla* or paramilitary) OR KW (terror* or extremism or extremist* or "radical" or radicals or radicali* or "political violence" or "lone actor*" or lone-actor* or Jihadi or Salafi or "right wing" or right-wing or separatist* or nationalist* or religio* or guerrilla* or paramilitary) OR SU (terror* or extremism or extremist* or "radical" or radicals or radicali* or "political violence" or "lone actor*" or lone-actor* or Jihadi or Salafi or "right wing" or right-wing or separatist* or nationalist* or religio* or guerrilla* or paramilitary) |
| 14 | TI (case-control or "case control" or correlation or coefficient* or cohort* or covariat* or cross-section* or "cross section*" or empirical or longitudinal or multivariate or odds or paramet* or predict* or prevalen* or prospective or questionnaire* or quantitative or rate* or regression or retrospective or risk* or survey* or sampl* or "standard deviation*" or statistic* or variable* or variance) OR AB (case-control or "case control" or correlation or coefficient* or cohort* or covariat* or cross-section* or "cross section*" or empirical or longitudinal or multivariate or odds or paramet* or predict* or prevalen* or prospective or questionnaire* or quantitative or rate* or regression or retrospective or risk* or survey* or sampl* or "standard deviation*" or statistic* or variable* or variance) OR KW (case-control or "case control" or correlation or coefficient* or cohort* or covariat* or cross-section* or "cross section*" or empirical or longitudinal or multivariate or odds or paramet* or predict* or prevalen* or prospective or questionnaire* or quantitative or rate* or regression or retrospective or risk* or survey* or sampl* or "standard deviation*" or statistic* or variable* or variance) OR SU (case-control or "case control" or correlation or coefficient* or cohort* or covariat* or cross-section* or "cross section*" or empirical or longitudinal or multivariate or odds or paramet* or predict* or prevalen* or prospective or questionnaire* or quantitative or rate* or regression or retrospective or risk* or survey* or sampl* or "standard deviation*" or statistic* or variable* or variance) |
| 15 | 12 and 13 and 14 |

**APPENDIX B. Determination of independent findings**

We excluded Simi et al. (2015) and Simi et al. (2016) from the data synthesis as the corresponding author confirmed that the data in those studies were expanded upon in their later paper (Bubolz & Simi, 2019) which was included.

In 2014 Gill et al. published their paper *Bombing alone* based on a dataset of 119 lone-actor terrorists from the US and EU. This sample included lone-actors who acted as individuals or as isolated dyads. In that paper, they report prevalence rates of mental illness for 11 isolated dyads, 87 individuals who acted alone without command and control links, and 21 individuals with command and control links. In 2015, Corner and Gill’s paper, *A false dichotomy*, drew on the same sample and compared the rates of disorder to a sample of group-based actors. The sample of group-based actors is unique to papers from this team of researchers and so is included in the data synthesis here.

The sample of lone-actors is not-unique as some are used in Gill’s subsequent (2015) book (*Lone-actor terrorist: A behavioural analysis*), and Corner et al.’s (2019) paper *The multi-finality of vulnerability indicators in lone actor terrorism.* In their 2015 book Gill drew on a sample of 111 lone-actors and that included some of the 2014 lone-actor sample but excluded ‘isolated dyads’ and individuals who acted alone ‘but in facilitative roles’ (p. 20). As such the 2014 *Bombing alone* paper and 2015 *Lone-actor terrorist* lone-actor samples are not unique. However, Gill is clear that the ‘Isolated Dyads’ sample was omitted from the 2014 paper in this book (and thus also *A false dichotomy*) and that sub-sample of 11 individuals is unique and is included in the data synthesis.

In 2019 Corner, Bouhana and Gill’s paper *The multi-finality of vulnerability indicators in lone actor terrorism,* took Gill’s 111 lone-actors and expanded this dataset to 125 individuals. Clemmow, Bouhana and Gill’s (2020) *Analyzing person-exposure patterns in lone-actor terrorism* used the same 125 individuals. As the 125 individuals included in Corner, Bouhana and Gill (2019) includes all of the Lone-Actor Terrorist (2015) data, we excluded that book from the meta-analysis. We also excluded Clemmow, Bouhana and Gill (2020) in favour of the Corner, Bouhana and Gill (2019) paper. The latter provided prevalence data for the full sample and the most complete data on mental health difficulties. We also excluded Gill, Silver, Horgan, Corner and Bouhana’s (2021) *Similar crimes, similar behaviors?* and Horgan and colleagues (2016) comparison of lone actors and mass murderers (*Across the Universe?*) from the data synthesis (though *Across the universe?* is considered under Objective 3 as it is a comparative study) as the data is included in *Bombing alone* and overlaps with the aforementioned included papers.

In conclusion, for the data synthesis for Objective 1 and (where relevant) Objective 2, we retained 11 individuals who operated in isolated dyads as a sample of lone-actors from Gill, Horgan and Deckert (2014), a sample of group-based terrorists from Corner and Gill’s (2015) paper, and a sample of 125 lone-actors from Corner et al.’s (2019) paper. We also include Horgan et al. (2016) under Objective 3.

Corner and Gill’s 2020 paper, *Psychological distress, terrorist involvement and disengagement from terrorism*, is not on lone-actor terrorism and presents a unique dataset based on 91 autobiographies of terrorists or former terrorists. However, this is the same dataset as their 2021 paper, *Psychological distress and terrorist engagement*, and the former is used here as it reports prevalence rates of mental illness/mental disorder. Decisions in relation to treatment of the Gill and colleagues papers were recommended by KS and SC concurred. Paul Gill also concurred when asked to advise on treatment of the papers.

Weenink produced two papers on Jihadis who travelled, or attempted to travel, to Syria. In their 2015 study of 140 Jihadis, they reported that 6% of the sample had a clinically diagnosed disorder. In this subsequent paper of 319 Jihadis, the reported rates of psychological problems as 28%, noting that the increase from 6% in the earlier paper was due to changes in coding and where a diagnosis was not required. For this reason, we include the 2015 paper in our data synthesis of diagnosed disorder and the 2019 paper for data synthesis of psychological problems.

Candilis et al. (2021) and Dhumad et al. (2020) used the same dataset of 120 terrorist offenders, both lone-actor and group-based, who had been incarcerated in Bagdad, Iraq. We used the latter in the data synthesis as it contained more detail on the mental health difficulties of the sample. Merari (2021) summarises their experiences and findings from two previous studies of Palestinian terrorists. We included the original studies in the data synthesis (Merari & Ganor, 2020; Merari et al., 2010).

Three papers on lone actor terrorism (ideological mass shooters) by Capellan were eligible for inclusion (Capellan, 2015; Capellan & Anisin, 2017; Capellan et al., 2019). Capellan confirmed that 40 participants in the 2015 paper and 45 participants in the 2017 paper are included in their 2019 paper and the authors provided data for positive cases of diagnosed disorder and suspected problems based on the *n* = 47. We excluded the two earlier papers in the data synthesis.

Four papers reporting on ten samples used the Profiles of Individual Radicalisation in the United States (PIRUS) dataset. LaMontange (2019) reports prevalence of psychological problems for 4 different samples, Jihadi/Islamist (*n* = 455), Far-Right (*n* = 746), Far-Left (*n* = 324) and Single-Issue (*n* = 340). In an earlier paper, La Free et al. (2018) also use PIRUS, reporting on 1,473 terrorists covering the period 1948-2013. The sample was a mixed sample of Islamist, far-right, far-left and Single-Issue terrorists and based on the information provided, we have assumed that all of the data in this study also appears in LaMontange’s (2019) sample. However, LaMontange reports levels of psychological problems and La Free et al. reports mental disorders, so each is used in the separate analyses (i.e., neither are used in the same analysis).

Jensen and Kane (2021) also use PIRUS, reporting on 1,715 far-right, far-left and single-issue offenders (reported as one-group), and comparing their profile with the profile of QAnon-inspired offenders. The QAnon dataset is unique and so is retained for the data-synthesis. However, there is an overlap in the rest of the Jensen and Kane (2021) and LaMontange (2019) datasets as both report levels of psychological problems. We have excluded the Jensen and Kane dataset on far-right, far-left and single-issue offenders from the data synthesis (retained for the narrative synthesis) in favour of the LaMontange dataset as the latter disaggregates difficulties for the different forms of terrorism.

Pendergast (2020) reports only on far-right terrorists from the PIRUS dataset. It is more up-to-date (1948-2017, *n* = 922) than the LaMontange (2019) study (1948-2016, *n* = 746) and so that sample of far-right terrorists displaces the LaMontagne sub-sample of far-right terrorists from the PIRUS dataset. Håugstvedt and Koehler (2021) isolated a sub-sample of extremists with a history of military from the PIRUS dataset, and also compared far-right extremists with military backgrounds with far-right extremists with no military backgrounds in terms of life-time prevalence of psychological problems. Again, we assessed that Pendergast’s (2020) and LaMontagne’s (2021) studies included these participants and the Håugstvedt and Koehler (2021) study was therefore excluded from the data synthesis.

From the overall PIRUS dataset, then, all papers are included in the narrative synthesis but Pendergast’s (2020) far-right sample, LaMontange’s (2019) far-left, single-issue and Jihadi samples, and Jensen and Kane’s (2021) QAnon sample are retained for the data synthesis.

Similar overlap arose with papers using the Extremist Crime Database (ECDB), which is a second open-source database of incidents ‘committed by domestic extremists in the United States (Chermak & Gruenwald, 2015). Chermak and Gruenewald report on levels of mental disorder among right-wing (*n* = 637), left-wing (*n* = 182) and Jihadi (*n* = 155) terrorists (1990-2011). They also report rates for the full sample. Freilich et al. (2019) also report prevalence rates based on the ECDB. Their sample is mixed and comprised of right-wing and Al Qaeda/associates (1990-2013) (*n* = 447). As the central objective of that paper was to compare suicide and non-suicide attackers, and Chermak & Gruenewald (2015) report for three different forms of terrorism, we excluded Freilich et al. (2019) from the data synthesis of prevalence rates of mental disorder (it is included in the analyses of psychological problems, also reported in the paper). We also excluded Lucas’s (2021) sample of right-wing suicide attackers in the US (*n* = 16), drawn from the ECDB, as at least some of that sample will be captured in the larger n studies. Gruenewald et al. (2013) use the ECDB and report rates of suspected disorder for right-wing lone actors and right-wing group actors. Where the data synthesis combines both diagnosed disorders and suspected disorder, we exclude Gruenewald et al. (2013) in favour of Chermak and Gruenewald (2015) as the latter reported mental disorders rather than suspected disorders. We do this on the assumption that the sample in Gruenewald et al. (2013) is included in Chermak and Gruenewald’s sample of right-wing terrorists.

**APPENDIX C. Full-Text Coding Form**

**General Study Details**

| Field | Response Options | TYPE |
| --- | --- | --- |
| Document ID |  | Numeric |
| Coder | - - - 1. SC       2. KC       3. KS | Categorical |
| Date Coded |  | Numeric |
| Study Author |  | Text |
| Study Title |  | Text |
| Publication Type | 1. Book  2. Book Chapter  3. Journal Article (peer reviewed)  4. Government Report (not police/security)  5. Police/Security Service Report  6. Academic Thesis Doctoral Level  7. Academic Thesis Other Level  8. Report of Expert Network  9. Conference Paper  10. Other | Categorical |
| Publication Other Specify |  | Text |
| Journal Name |  | Text |
| Journal Volume |  | Numeric |
| Journal Issue |  | Numeric |
| Jurisdiction Data Gathered |  | Text |
| Date Range Data Gathered | Commence; Finish | Numeric |
| Funding Source |  | Text |
| Data Source | - - - 1. Police Database       2. Self-Report Questionnaire       3. Case Notes       4. Media Reports       5. Court Reports       6. Other | Categorical |
| Data Source Other Specifcy |  | Text |
| Data reported in other study | 1.Yes  2.No | Categorical |
| Data reported in other study Yes | Author, Year | Text |

**Sample**

| Field | Response Options | TYPE |
| --- | --- | --- |
| Population Size |  | Numeric |
| Description of Sample | 1. Prisoners  2. Former Terrorists  3. Active Terrorists  4. Other | Categorical |
| Description of Sample Other |  | Text |
| Gender | - - - 1. Male       2. Female       3. Both       4. Other | Categorical |
| Gender Other Specify |  | Text |
| Gender breakdown | % Male, % Female, % Other | Text |
| Ethnicity |  | Text |
| Age | Mean; SD; Range | Numerical |
| Developmental age | 1. 0-17 Child/Adolescent  2. 18+ Adult |  |

**Outcome**

| Field | Response Options | TYPE |
| --- | --- | --- |
| Definition of Terrorism |  | Text |
| Type of Terrorism | Lone actor  Islamic  Right Wing   - - - 1. Separatist       2. Mixed       3. Other | Categorical |
| Type Mixed Specify |  | Text |
| Type Lone Actor Specify |  | Text |
| Type Other Specify |  | Text |
| Role | (e.g., bombing; logistics; finance; propaganda; unknown; mixed). | Text |

**Risk Factor**

| Field | Response Options | TYPE |
| --- | --- | --- |
| Diagnosed Mental Disorders | Yes  No  Unclear | Categorical |
| Yes Disorders Specify | 1. Neurodevelopmental Disorder  2. Intellectual Disability  3. Communication Disorder  4. Autism Spectrum Disorder  5. Attention-Deficit/Hyperactivity Disorder  6. Specific Learning Disorder  7. Motor Disorder  8. Other Neurodevelopmental Disorder  9. Schizophrenia or Psychotic Disorder  10. Bipolar and Related Disorder  11. Depressive Disorder  12. Anxiety Disorder  13. Obsessive-Compulsive and Related Disorders  14. Trauma and Stressor-Related Disorders  15. Dissociative Disorder  16. Somatic Symptom and Related Disorders  17. Feeding and Eating Disorders  18. Elimination Disorders  19. Sleep-Wake Disorders  20. Gender Dysphoria  21. Disruptive, Impulse-Control and Conduct Disorders  22. Substance-Related and Addictive Disorders  23. Neurocognitive Disorders  24. Cluster A Personality Disorder  25. Cluster B Personality Disorder  26. Cluster C Personality Disorder  27. Other Personality Disorder  28. Paraphilic Disorders  29. Other | Categorical (select all that apply) |
| Other Specify |  | Text |
| Diagnosed by mental health professional | 1. Yes  2. No | Categorical |
| Diagnosed by prof. Yes Describe | e.g., Clinical interview, case note review, testing | Text |
| Diagnosed by prof No Describe | Justify inclusion as disorder | Text |
| Co-Morbidity |  | Text |
| Psychological Problems | 1. Worried/Afraid  2. Unhappy/Sad  3. Emotional  4. Quiet/Withdrawn  5. Guilty/Worthless  6. Suicide  7. Suicidal Behaviour  8. Self-harm  9. Mood  10. Affective  11. Addiction  12. Other | Categorical (select all that apply) |
| Problems Other Specify |  | Text |
| Problems vs. Clinical Psychological Problems? |  | Text |
| How problems determined in study | - - - 1. 1.Open Source       2. 2.Offical Data (including police reports; court reports etc.)   3.Family/Peer reports   - - - 1. 4.Mental Health records   5.Other | Categorical (select all that apply) |
| Problems determined other specify |  | Text |
| Onset of disorder relative to terrorism involvement | 1.Pre  2.During  3.Post  4.Not clear  5.Mixed |  |
| Onset Disorder not clear or mixed Specify |  | Text |
| Onset of problems relative to terrorism involvement | 1.Pre  2.During  3.Post  4.Not clear  5.Mixed | Categorical |
| Onset problems not clear or mixed Specify |  | Text |

**Study Design**

| Field | Response Options | TYPE |
| --- | --- | --- |
| Design | 1.Cross-Sectional  2.Cohort Retrospective  3.Cohort Prospective  4.Case-Control  5.Other | Categorical |
| Design Description |  | Text |
| Design Other |  | Text |
| Relevant Objective 1. | 1. Study estimates the proportion of sample terrorist population that has a mental health difficulty/disorder (i.e., Include in Objective 1). | Categorical |
| Relevant Objective 2. | 1. Study estimates the association between mental health difficulties disorder and terrorist behaviour (i.e., Include in Objective 2). | Categorical |
| Time Points Retrospective |  | Text |
| Time Points Prospective |  | Text |
| Control Sample Type |  | Text |
| Control Sample Size |  | Text |
| Control Sample Data Source |  | Text |
| Control Sample Data Type | (e.g., prevalence, point-prevalence etc.) | Text |
| Control Sample Diagnostic Approach | (e.g., Clinical interview) | Text |
| Control Sample diagnosis(es) |  | Text |
| Control Sample problems |  | Text |
| Control Sample Temporal onset | (e.g., prior to incarceration; childhood etc.) | Text |
| Control Sample duration of disorder |  | Text |
| Control Sample duration of problems |  | Text |

**Findings**

| Field | Response Options | TYPE |
| --- | --- | --- |
| If case control, was difference significant | 1.Yes  2.No  3.Unclear  4.Not tested | Categorical |
| If multiple tests, describe all |  | Text |
| If case control, was standardised effect size reported? | 1.Yes  2.No | Categorical |
| Significance Tests reported | 1.t-value  2.F-value  3. Chi-Square value |  |
| If multiple tests, describe all |  | Text |
| If ‘Yes’ case control effect what measure | 1.r  2.b  3.OR  4.Other | Categorical |
| Effect size | (e.g., Test 1; Test 2; Test 3 etc.) | Numerical |
| Standard error of effect | (e.g., SE1; SE2; SE3 etc.) | Numerical |
| Page number(s) effects reported |  | Numerical |
| If no effect, does data allow effects to be calculated? | 1.Yes  2.No | Numerical |
| Point Prevalence Rate Reported for all Disorders + Confidence Intervals |  | Numerical |
| Point Prevalence Rate Reported for specific Disorders + Confidence Intervals |  | Numerical |
| Point Prevalence Rate Reported for all Problems + Confidence Intervals |  | Numerical |
| Point Prevalence Rate Reported for specific Problems + Confidence Intervals |  | Numerical |
| Period Prevalence Rate Reported for all Disorders + Confidence Intervals |  | Numerical |
| Period Prevalence Rate Reported for specific Disorders + Confidence Intervals |  | Numerical |
| Period Prevalence Rate Reported for all Problems + Confidence Intervals |  | Numerical |
| Period Prevalence Rate Reported for specific Problems + Confidence Intervals |  | Numerical |
| Plausibility arguments | 1.Presents theoretical link  2.Does not present theoretical link  3.Unclear |  |
| Plausibility arguments describe | Summarise author(s)’ argument on link between mental health difficulties and terrorism with reference to a) predisposing, b) precipitating, c) perpetuating and d) protective processes. | Text |
| Findings describe | Summarise key findings | Text |
| Author(s)’ conclusions | 1.Mental health difficulties confers risk  2.Mental health difficulties does not confer risk  3.Mental heath difficulties reduces risk  4.Evidence insufficient  5.No conclusions | Categorical |
| Author(s)’ conclusions expanded | Summarise key conclusions | Text |

**Risk of Bias (Prevalence)**

| Field | Response Options | TYPE |
| --- | --- | --- |
| Was the sample representative of the target population? | 1.Yes  2.No  3.Unclear  4.Not Applicable | Categorical |
| Were study participants recruited in an appropriate way? | 1.Yes  2.No  3.Unclear  4.Not Applicable | Categorical |
| Was the sample size adequate? | 1.Yes  2.No  3.Unclear  4.Not Applicable | Categorical |
| Were the study subjects and the setting described in detail? | 1.Yes  2.No  3.Unclear  4.Not Applicable | Categorical |
| Was the data analysis conducted with sufficient coverage of the identified sample? | 1.Yes  2.No  3.Unclear  4.Not Applicable | Categorical |
| Were objective, standard criteria used for the measurement of the condition? | 1.Yes  2.No  3.Unclear  4.Not Applicable | Categorical |
| Was the condition measured reliably? | 1.Yes  2.No  3.Unclear  4.Not Applicable | Categorical |
| Was there appropriate statistical analysis? | 1.Yes  2.No  3.Unclear  4.Not Applicable | Categorical |
| Are all important confounding factors/subgroups/differences identified and accounted for? | 1.Yes  2.No  3.Unclear  4.Not Applicable | Categorical |
| Were subpopulations identfied using objective criteria? | 1.Yes  2.No  3.Unclear  4.Not Applicable | Categorical |
| Did the authors present a plausible argument linking the condition with the outcome | 1.Yes  2.No  3.Unclear  4.Not Applicable | Categorical |

**Risk of Bias – Cohort**

| Field | Response Options | TYPE |
| --- | --- | --- |
| Were the two groups similar and recruited from the same population? | 1.Yes  2.No  3.Unclear  4.Not Applicable | Categorical |
| Were the exposures measured similarly to assign people to both exposed and unexposed groups? | 1.Yes  2.No  3.Unclear  4.Not Applicable | Categorical |
| Was the exposure measured in a valid and reliable way? | 1.Yes  2.No  3.Unclear  4.Not Applicable | Categorical |
| Were confounding factors identified? | 1.Yes  2.No  3.Unclear  4.Not Applicable | Categorical |
| Were strategies to deal with confounding factors stated? | 1.Yes  2.No  3.Unclear  4.Not Applicable | Categorical |
| Were the groups/participants free of the outcome at the start of the study (or at the moment of exposure)? | 1.Yes  2.No  3.Unclear  4.Not Applicable | Categorical |
| Were the outcomes measured in a valid and reliable way? | 1.Yes  2.No  3.Unclear  4.Not Applicable | Categorical |
| Was the follow up time reported and sufficient to be long enough for outcomes to occur? | 1.Yes  2.No  3.Unclear  4.Not Applicable | Categorical |
| Was follow up complete, and if not, were the reasons to loss to follow up described and explored? | 1.Yes  2.No  3.Unclear  4.Not Applicable | Categorical |
| Were strategies to address incomplete follow up utilized? | 1.Yes  2.No  3.Unclear  4.Not Applicable | Categorical |
| Was appropriate statistical analysis used? | 1.Yes  2.No  3.Unclear  4.Not Applicable | Categorical |

**Risk of Bias – Cross Sectional**

| Field | Response Options | TYPE |
| --- | --- | --- |
| Were the criteria for inclusion in the sample clearly defined? | 1.Yes  2.No  3.Unclear  4.Not Applicable | Categorical |
| Were ere the study subjects and the setting described in detail? | 1.Yes  2.No  3.Unclear  4.Not Applicable | Categorical |
| Was the exposure measured in a valid and reliable way? | 1.Yes  2.No  3.Unclear  4.Not Applicable | Categorical |
| Were objective, standard criteria used for measurement of the condition? | 1.Yes  2.No  3.Unclear  4.Not Applicable | Categorical |
| Were confounding factors identified? | 1.Yes  2.No  3.Unclear  4.Not Applicable | Categorical |
| Were strategies to deal with confounding factors stated? | 1.Yes  2.No  3.Unclear  4.Not Applicable | Categorical |
| Were the outcomes measured in a valid and reliable way? | 1.Yes  2.No  3.Unclear  4.Not Applicable | Categorical |
| Was appropriate statistical analysis used? | 1.Yes  2.No  3.Unclear  4.Not Applicable | Categorical |

**Risk of Bias – Case Control**

| Field | Response Options | TYPE |
| --- | --- | --- |
| Were the groups comparable other than the presence of disease in cases or the absence of disease in controls? | 1.Yes  2.No  3.Unclear  4.Not Applicable | Categorical |
| Were cases and controls matched appropriately? | 1.Yes  2.No  3.Unclear  4.Not Applicable | Categorical |
| Were the same criteria used for identification of cases and controls? | 1.Yes  2.No  3.Unclear  4.Not Applicable | Categorical |
| Was exposure measured in a standard, valid and reliable way? | 1.Yes  2.No  3.Unclear  4.Not Applicable | Categorical |
| Was exposure measured in the same way for cases and controls? | 1.Yes  2.No  3.Unclear  4.Not Applicable | Categorical |
| Were confounding factors identified? | 1.Yes  2.No  3.Unclear  4.Not Applicable | Categorical |
| Were strategies to deal with confounding factors stated? | 1.Yes  2.No  3.Unclear  4.Not Applicable | Categorical |
| Were outcomes assessed in a standard, valid and reliable way for cases and controls? | 1.Yes  2.No  3.Unclear  4.Not Applicable | Categorical |
| Was the exposure period of interest long enough to be meaningful? | 1.Yes  2.No  3.Unclear  4.Not Applicable | Categorical |
| Was appropriate statistical analysis used? | 1.Yes  2.No  3.Unclear  4.Not Applicable | Categorical |
